# Redox-inactive CC-type glutaredoxins interfere with TGA transcription factor–dependent repression of target promoters in roots

**DOI:** 10.1093/plcell/koaf038

**Published:** 2025-03-07

**Authors:** Corinna Thurow, Anja Maren Pelizaeus, Pascal Mrozek, Ben Moritz Hoßbach, Jelena Budimir, Kerstin Schmitt, Oliver Valerius, Gerhard Braus, Christiane Gatz

**Affiliations:** Albrecht-von-Haller-Institut für Pflanzenwissenschaften, Georg-August-Universität Göttingen, Julia-Lermontowa-Weg 3, D-37077 Göttingen, Germany; Albrecht-von-Haller-Institut für Pflanzenwissenschaften, Georg-August-Universität Göttingen, Julia-Lermontowa-Weg 3, D-37077 Göttingen, Germany; Albrecht-von-Haller-Institut für Pflanzenwissenschaften, Georg-August-Universität Göttingen, Julia-Lermontowa-Weg 3, D-37077 Göttingen, Germany; Albrecht-von-Haller-Institut für Pflanzenwissenschaften, Georg-August-Universität Göttingen, Julia-Lermontowa-Weg 3, D-37077 Göttingen, Germany; Albrecht-von-Haller-Institut für Pflanzenwissenschaften, Georg-August-Universität Göttingen, Julia-Lermontowa-Weg 3, D-37077 Göttingen, Germany; Institut für Mikrobiologie und Genetik, Serviceeinheit LCMS Proteinanalytik, Georg-August-Universität Göttingen, Grisebachstraße 8, D-37077 Göttingen, Germany; Institut für Mikrobiologie und Genetik, Serviceeinheit LCMS Proteinanalytik, Georg-August-Universität Göttingen, Grisebachstraße 8, D-37077 Göttingen, Germany; Institut für Mikrobiologie und Genetik, Serviceeinheit LCMS Proteinanalytik, Georg-August-Universität Göttingen, Grisebachstraße 8, D-37077 Göttingen, Germany; Albrecht-von-Haller-Institut für Pflanzenwissenschaften, Georg-August-Universität Göttingen, Julia-Lermontowa-Weg 3, D-37077 Göttingen, Germany

## Abstract

Changes in nitrogen (N) availability in the soil trigger transcriptional responses in plants to optimize N acquisition, allocation, and remobilization. In roots of N-starved Arabidopsis (*Arabidopsis thaliana*) plants, transcriptional activation of genes encoding, for example, low-affinity nitrate transporters, depends on 4 related C-TERMINALLY ENCODED PEPTIDE DOWNSTREAM (CEPD) proteins, also known as ROXY6, ROXY7, ROXY8, and ROXY9. All 21 ROXYs found in *A. thaliana* interact with members of the TGACG-binding (TGA) family of transcription factors. Here, we demonstrate that 2 Clade I TGAs (TGA1, TGA4) serve as molecular links between CEPDs and their target promoters in roots. In the *roxy6 roxy7 roxy8 roxy9* quadruple mutant (named *cepd* in this manuscript), transcriptional activation of N-starvation-inducible genes is impaired, most likely due to the association of Clade I TGAs with a repressive complex at their target promoters. In wild-type plants, this repressive complex is nonfunctional, and gene expression may be regulated by the N supply-regulated ratio of CEPDs over opposing ROXYs containing the TOPLESS-interacting ALWL motif. Although CEPDs resemble glutaredoxins with glutathione-dependent oxidoreductase activity, a ROXY9 variant with a mutation in the catalytic cysteine in its putative active site can confer wild-type-like regulation of target genes. This finding demonstrates that ROXY9 does not function through redox-dependent mechanisms.

## Teaser

C-TERMINALLY ENCODED PEPTIDE DOWNSTREAM (CEPD) proteins activate gene expression in roots by revoking a repressive mechanism mediated by the TGACG-binding transcription factors TGA1 and TGA4.

## Introduction

As autotrophic organisms, plants depend on the uptake of inorganic molecules. Nitrogen (N) is an essential macronutrient that is taken up by plants as ammonium (${\mathrm{NH}}_{4}^{+}$) or nitrate (${\mathrm{NO}}_{3}^{-}$) (Maathuis 2009). The ability of plants to adapt N uptake and assimilation to N availability (Fredes et al. 2019) constitutes an important trait of crop species. In the model plant Arabidopsis (*Arabidopsis thaliana*), the expression of high-affinity ${\mathrm{NO}}_{3}^{-}$ transporter genes can be induced either by N starvation (Ohkubo et al. 2017) or by the addition of ${\mathrm{NO}}_{3}^{-}$ to the roots of N-starved plants (Krouk et al. 2006). One of the underlying regulatory mechanisms operating under N starvation involves the action of C-TERMINALLY ENCODED PEPTIDE DOWNSTREAM (CEPD) proteins, which belong to the family of plant-specific glutaredoxin-like proteins (Ohkubo et al. 2017, 2021; Ota et al. 2020).

Glutaredoxins (GRXs) are small proteins (12 to 15 kDa) found in all domains of life. Class I glutaredoxins, which contain variants of the CPYC motif in their active sites at the beginning of α helix 2, are catalytically active oxidoreductases that use glutathione (GSH) as a co-factor. Class II glutaredoxins, which contain a highly conserved CGFS motif in the corresponding position, bind labile [2Fe–2S] clusters for transfer to client proteins (Rodriguez-Manzaneque et al. 2002; Supplementary Fig. S1).

According to sequence alignments and phylogenetic analysis, land plants contain a third group of glutaredoxins. These are called CC-type glutaredoxin-like proteins because of their conserved CCM/LC or CCM/LS motif instead of the CPYC or CGFS signatures mentioned above (Rouhier et al. 2008; Supplementary Fig. S1). The few available biochemical studies of these proteins have reported either weak (Couturier et al. 2010) or no oxidoreductase activity in enzymatic assays using the standard substrates bis(2-hydroxyethyl)disulfide or the redox-sensitive green fluorescent protein variant roGFP (Xu et al. 2022; Mrozek et al. 2025). The family of CC-type glutaredoxin-like proteins has expanded during land plant evolution and is represented by 21 members (called ROXYs) in Arabidopsis (Ziemann et al. 2009). They are located in the cytosol and the nucleus (Li et al. 2009; Ohkubo et al. 2017; Xu et al. 2022) and biochemically interact with TGACG-binding transcription factors (TGAs; Ndamukong et al. 2007; Li et al. 2009; Yang et al. 2021). Epistasis analysis has provided evidence that the interaction between CC-type glutaredoxin-like proteins and TGAs has functional relevance: for instance, Arabidopsis ROXY1 alleviates the repressive activity of the TGA transcription factor PERIANTHIA (PAN) on petal primordia initiation (Li et al. 2009). The maize (*Zea mays*) CC-type glutaredoxin MALE STERILE CONVERTED ANTHER1 (MSCA1) and its 2 orthologs ZmGRX2 and ZmGRX5 negatively regulate the activity of the TGA factor FASCIATED EAR 4 (FEA4) to control meristem size (Yang et al. 2021).

Recently, members of a subclade of highly related ROXYs (ROXY6, ROXY7, ROXY8, and ROXY9, collectively termed ROXYs 6 to 9) were found to be important for nitrate acquisition on medium with high, moderate, or low N supply (Ota et al. 2020). These ROXYs were designated CEPD or CEPD-like (CEPDL), because 2 act DOWNSTREAM (D) of the C-TERMINALLY ENCODED PEPTIDE (CEP; Ohkubo et al. 2017). CEPs are N starvation–induced 15-amino acid peptide hormones that act as a root-derived N demand signal. CEPs are transported to the shoot, where they activate signaling through the leucine-rich receptor kinases CEP RECEPTOR 1 (CEPR1) and CEPR2 (Tabata et al. 2014), leading to the upregulated expression of *CEPD1* (previously named *ROXY6*) and *CEPD2* (previously named *ROXY9*; Ohkubo et al. 2017). Transcription of *CEPDL2* (previously named *ROXY8*) is induced upon sensing of the N status of the shoot (Ota et al. 2020). CEPDs travel through the phloem to the roots and activate the expression of genes involved in nitrate uptake, such as the nitrate transporter gene *NRT2.1* and the phosphatase gene *CEPD-INDUCED PHOSPHATASE* (*CEPH*; Ohkubo et al. 2017, 2021; Ota et al. 2020), whose protein product enhances NRT2.1 transport activity via dephosphorylation (Ohkubo et al. 2021).

Analysis of publicly available transcriptome data previously led to the hypothesis that Clade I TGAs play a role in the nitrate response of Arabidopsis, although nitrate uptake is not impaired in the *tga1 tga4* mutant (Alvarez et al. 2014). TGA1 and/or TGA4 are part of a nitrate-induced signaling cascade leading to increased initiation of lateral roots (Alvarez et al. 2014), higher root hair density (Canales et al. 2017), and enhanced N dose-responsive gene expression (Swift et al. 2020). Here, we tested whether CEPDs regulate Clade I TGA activity in the context of the N starvation response. We show that Clade I TGAs transcriptionally repress a set of N transport–related genes. CEPDs are required to relieve this repression, most likely by interfering with the activity of a TGA1/TGA4-interacting repressive complex.

## Results

### CEPDs interfere with the repressive action of TGA1 and TGA4 on shoot fresh weight and nitrate accumulation

To investigate whether CEPDs regulate TGA1 and/or TGA4 activity, we performed an epistasis analysis. Since defects in nitrate acquisition and growth of Arabidopsis (Nossen accession) seedlings are most pronounced when all 4 *CEPD* genes are mutated (Ota et al. 2020), we focused on the analysis of a *roxy6 roxy7 roxy8 roxy9* quadruple mutant, which we generated in the Col-0 background through clustered regularly interspaced short palindromic repeat (CRISPR)-associated nuclease 9 (Cas9)-based genome editing. We subsequently crossed this quadruple mutant, referred to hereafter as *cepd*, to the *tga1 tga4* double mutant (Shearer et al. 2012) to obtain the *cepd tga1 tga4* hextuple mutant.

**Figure 1.**
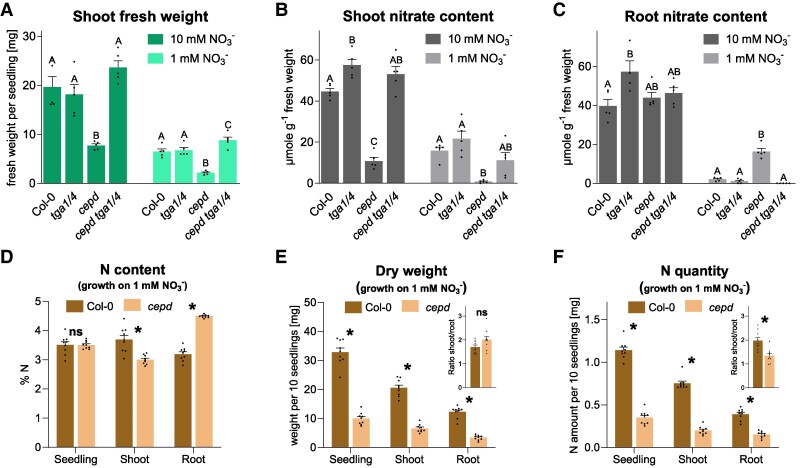
CEPDs counteract the repressive effect of TGA1/4. The indicated genotypes (Col-0, *tga1 tga4*, *cepd*, and *cepd tga1 tga4*) were grown for 21 d **(A** to **C)** or 19 d **(D** to **F)** under constant light (70 *µ*mol photons s^−1^ m^−2^) on vertically oriented MS plates with either 10 mm  ${\mathrm{NO}}_{3}^{-}$ or 1 mm  ${\mathrm{NO}}_{3}^{-}$ as N source. Fresh and dry weight, nitrate, and N were measured in shoots and roots as indicated. Mean values of 5 **(A** to **C)** or 9 **(D** to **F)** biological replicates are shown, with 1 replicate originating from 1 plate with 10 seedlings. Inserts in **E)** and **F)** indicate the shoot/root ratios. Error bars in **A)** to **F)** represent the standard error of the mean. In **A)** to **C)**, statistical analyses were performed separately for each growth condition using 1-way ANOVA and Tukey's multiple comparisons test. Letters indicate significant differences (*P* adj. < 0.05) between the genotypes. In **D)** and **F)**, statistical analyses were performed using multiple unpaired *t*-tests. Stars indicate significant differences between Col-0 and *cepd*. ns, not significant (*P* < 0.05).

The fresh weight of *cepd* shoots was lower than that of Col-0, regardless of nitrate supply, provided as 10 or 1 mm  ${\mathrm{NO}}_{3}^{-}$ in Murashige and Skoog (MS) medium (Fig. 1A). It was also lower when plants were grown on fertilized soil (Supplementary Fig. S2). Notably, the *cepd tga1 tga4* hextuple mutant produced the same amount of fresh biomass as Col-0, indicating that the poor growth of *cepd* is due to the repressive effect of TGA1 and/or TGA4 (from here on referred to as TGA1/4) on genes required for wild-type-like growth. The nitrate content was also lower in *cepd* shoots than in Col-0 regardless of the amount of nitrate in the medium. This phenotype also returned to Col-0 levels in *cepd tga1 tga4* (Fig. 1B). In the shoots of *tga1 tga4* seedlings grown on MS medium containing 10 mm  ${\mathrm{NO}}_{3}^{-}$, the nitrate content was slightly higher than that in Col-0 shoots, confirming the negative role of TGA1/4. In the roots of *cepd* seedlings grown in the presence of 10 mm  ${\mathrm{NO}}_{3}^{-}$, the nitrate content was similar to that of Col-0, while it was slightly higher in *tga1 tga4*. Unexpectedly, nitrate content was 7.6-fold higher in *cepd* roots than in wild-type roots in the presence of only 1 mm  ${\mathrm{NO}}_{3}^{-}$. Nitrate content also returned to Col-0 levels in *cepd tga1 tga4* (Fig. 1C).

The elevated nitrate level in the roots of *cepd* seedlings grown on 1 mm  ${\mathrm{NO}}_{3}^{-}$ suggested that root-to-shoot allocation of N compounds was altered in this mutant. We confirmed this hypothesis when measuring total N per dry weight: the roots of *cepd* had a higher N content than Col-0, while the shoots of *cepd* had lower N levels than Col-0 shoots (Fig. 1D). However, this did not result in relatively better root growth of *cepd* (Fig. 1E). The N quantity per seedling was 2.5-fold lower in *cepd* than in Col-0, demonstrating that not only N allocation, but also nitrate uptake, was impaired (Fig. 1F).

### CEPDs are required for the expression of genes related to transport of inorganic ions including nitrate

To elucidate the molecular mechanism of CEPD action, we looked for marker genes that are stringently controlled by CEPDs. To this aim, we first searched for conditions with strong differential expression of *CEPD*s. According to publicly available expression data (www.genevestigator.de; Scheible et al. 2004; Supplementary Fig. S3A), *ROXY8* and *ROXY9* are more highly expressed in seedlings cultivated for 2 d in liquid MS medium containing 0.15 mm  ${\mathrm{NO}}_{3}^{-}$ and 0.05 mm NH_4_^+^ (corresponding to 0.2 mm N, low nitrogen [LN]) than in control seedlings grown in the presence of 3 mm  ${\mathrm{NO}}_{3}^{-}$, 1 mm NH_4_^+^, and 1 mm glutamine (corresponding to 6 mm N, full nutrition; Scheible et al. 2004). To investigate gene expression in shoots and roots separately, we grew seedlings on solid FN medium in square Petri plates. After 7 d of growth under continuous light, we transferred seedlings to either FN or LN medium. Two days later, we harvested shoots and roots for expression analysis.

As described for Nossen (Ohkubo et al. 2017; Ota et al. 2020), *CEPD* transcript levels were higher in Col-0 shoots than in roots, as determined by RT-qPCR analysis (Supplementary Fig. S3B). Upon transfer of seedlings to fresh medium, the transcript levels of *ROXY8* and *ROXY9* rose strongly in the shoots of seedlings transferred to LN medium when compared with those transferred to FN medium. In contrast, *ROXY6* expression was only slightly induced on LN medium, while *ROXY7* expression was independent of the N supply.

Since CEPDs are shoot-to-root mobile proteins that activate gene expression in the roots of N-starved plants, we performed transcriptome deep sequencing (RNA-seq) analysis of total RNA from roots from Col-0 and *cepd* seedlings subjected to the FN-to-LN transfer. We collected samples from 4 independent experiments, each consisting of roots from 50 seedlings grown on 5 separate plates for sequencing. This analysis revealed 350 genes as being downregulated and 212 genes as being upregulated in *cepd* compared with Col-0 (log_2_ fold change [FC] > 1 or <−1, adjusted *P*-value [*P* adj.] < 0.05, Supplementary Data Set 1).

According to a Gene Ontology (GO)-term enrichment analysis, genes involved in the transport of inorganic ions, including nitrate, were overrepresented among the 350 genes that were less expressed in *cepd* than in wild-type plants (Supplementary Fig. S4A). This finding is consistent with the established role of CEPDs in enhancing nitrate acquisition in Arabidopsis (Ota et al. 2020; Ohkubo et al. 2021). Among the 10 most highly differentially expressed genes (log_2_FC > 3.19), 5 encode transporters, including the high-affinity nitrate transporters NRT2.2 and NRT2.4 (Zhuo et al. 1999; Kiba et al. 2012), the amino acid transporter USUALLY MULTIPLE ACIDS MOVE IN AND OUT TRANSPORTERS 35 (UMAMIT35) (Zhao et al. 2021), and the ammonium transporter AMT1-5 (Yuan et al. 2007; Supplementary Fig. S4B). One gene encodes for the nitrate-sensitive transcription factor NIN-LIKE PROTEIN 3 (NLP3; Liu et al. 2022). The expression of *NRT2.1*, which plays a major role in high-affinity ${\mathrm{NO}}_{3}^{-}$ uptake in the root (Cerezo et al. 2001; Filleur et al. 2001), was less affected by the loss of CEPDs (log_2_FC 2.04) than was the expression of *NRT2.2* or *NRT2.4*. *CEPH*, encoding the phosphatase that activates high-affinity nitrate uptake by dephosphorylating NRT2.1 (Ohkubo et al. 2021), was also among the 10 most highly differentially regulated genes (log_2_ FC 3.22).

Finally, a motif mapper analysis (Berendzen et al. 2012) revealed the enrichment of the TGA factor–binding motifs TGACG, TGACGTCA, and TACGTA (Izawa et al. 1993; Wang et al. 2019) in the promoter regions of genes positively regulated by CEPDs (Supplementary Fig. S4C).

### CEPDs interfere with the repressive effect of TGA1/4 on the expression of N starvation–induced genes

**Figure 2.**
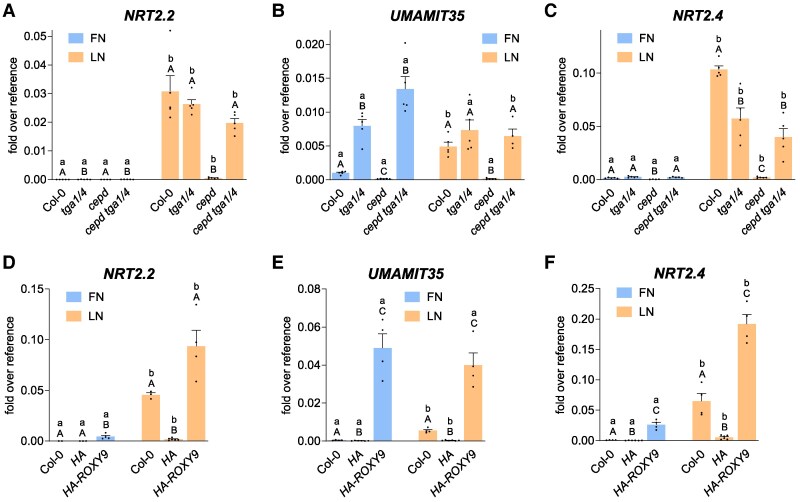
Impaired gene expression in *cepd* is due to the repressive effect of TGA1/4. Seven-day-old seedlings grown on full nitrogen (FN) medium under constant light (70 *µ*mol photons s^−1^ m^−2^) were transferred to either FN or LN plates. Two days later, roots were collected for RNA isolation. Expression of the indicated genes was analyzed by RT-qPCR, and *UBQ5* was used as a reference gene. **A)** to **C)** Mean values of 4 to 5 biological replicates are shown, with 1 replicate originating from 1 plate with 10 seedlings. **D)** to **F)** Mean values of 4 to 5 independent transgenic lines are shown, with each replicate comprising roots from 20 seedlings. **A)** to **F)** Error bars represent the standard error of the mean. Lowercase letters indicate statistically significant differences within the genotype between the treatments, and uppercase letters indicate significant differences within treatment between the genotypes. Statistical analyses were performed with the logarithmic values by using 2-way ANOVA and Bonferroni's multiple comparisons test (*P* adj. < 0.05). *HA*, empty vector transformed into *cepd*; *HA-ROXY9*, HA-tagged ROXY9 was expressed under the control of the *CaMV 35S* promoter in *cepd* plants.

We examined the expression pattern of the 3 most highly differentially expressed genes (Col-0 vs. *cepd*, log_2_FC > 4.26; Supplementary Fig. S4B) in the roots of *tga1 tga4*, *cepd*, and *cepd tga1 tga4* seedlings (Fig. 2, A to C). Under LN conditions, *NRT2.2* was highly induced in Col-0 and *tga1 tga4*, but not in *cepd* (Fig. 2A). The expression of *NRT2.2* in *cepd* reverted to wild-type levels in *cepd tga1 tga4*, suggesting that TGA1/4 strongly interfere with LN-induced *NRT2.2* expression and that this repressive effect is counteracted by CEPDs. Clearly, a CEPD- and TGA1/4-independent mechanism still conferred strong upregulation of *NRT2.2* expression after transfer from FN to LN conditions. LN-induced *NRT2.2* expression in *cepd* was rescued in seedlings constitutively expressing a HA-tagged *ROXY9* construct under the control of the *Cauliflower Mosaic Virus* (*CaMV*) *35S* promoter (Fig. 2D).

Similar *to NRT2.2*, *UMAMIT35* expression was induced under N starvation in Col-0, and this induction was severely compromised in *cepd* (Fig. 2B). Under FN conditions, *UMAMIT35* expression reached levels even higher than those in Col-0 in *tga1 tga4* and *cepd tga1 tga4*, whereas under LN conditions, *UMAMIT35* expression levels in Col-0 were similar to those in *tga1 tga4* and *cepd tga1 tga4*. The expression pattern of *UMAMIT35* can be explained by TGA1/4 repressing the transcriptional activation of this gene under FN conditions, while LN-induced CEPDs (Supplementary Fig. S3) counteract TGA1/4-mediated repression under LN conditions. In *cepd 35S:HA-ROXY9* seedlings, we observed high and N supply-independent *UMAMIT35* expression, supporting the notion that this promoter is regulated by the amount of CEPDs (Fig. 2E). This result is in contrast to the expression pattern of *NRT2.2*, which can be regulated by N starvation even in the absence of CEPD-regulated TGA1/4 activity and despite constitutive expression of *ROXY9* (Fig. 2, A and D).

The regulation of *NRT2.4* expression was similar to that of *NRT2.2*, showing the strongest differences among genotypes under LN conditions (Fig. 2C). *NRT2.4* expression was lower in *tga1 tga4* than in Col-0, indicating that TGA1/4 contributed to its transcriptional activation under LN conditions. TGA1/4 repressed *NRT2.4* expression in *cepd*, as revealed by the low *NRT2.4* expression levels in *cepd* and similarly high *NRT2.4* expression levels in *cepd tga1 tga4* and *tga1 tga4*. Constitutive expression of *ROYX9* under FN conditions was sufficient to activate the *NRT2.4* promoter, with a further induction under LN conditions (Fig. 2F).

We also asked whether the CEPD-TGA1/4-independent induction of *NRT2.2* after transfer from FN to LN medium might be due to glutamine and ammonium present in the FN medium, which have been reported to repress *NRT2.2* expression (Krouk et al. 2006). When we mimicked N sufficient conditions by growing seedlings on 10 mm  ${\mathrm{NO}}_{3}^{-}$, *NRT2.2* expression was 300-fold higher than in Col-0 plants grown on FN medium (Supplementary Fig. S5, A and B). Strikingly, expression levels in *tga1 tga4* seedlings grown on 10 mm  ${\mathrm{NO}}_{3}^{-}$ were elevated when compared with Col-0, reaching similar levels as in Col-0 grown on 1 mm  ${\mathrm{NO}}_{3}^{-}$ (Supplementary Fig. S5A). Thus, in the absence of the repressive effects of glutamine and ammonium, *NRT2.2* expression was repressed by TGA1/4 on sufficient N supply and this repression was counteracted by increased levels of CEPDs under N starvation conditions (Supplementary Fig. S5C).

Since *ROXY6* (also named *CEPD1*) and *ROXY9* (also named *CEPD2*) act downstream of CEPR1 in the Nossen Arabidopsis accession (Ohkubo et al. 2017), we investigated the expression of *CEPD* genes and the target genes of their encoded peptides in *cepr1-3*, a mutant in the Col-0 background. Although the N starvation–induced expression of *NRT2.2*, *UMAMIT35*, and *NRT2.4* was compromised in *cepr1-3* (Supplementary Fig. S6A), relative *ROXY8* and *ROXY9* transcript levels were even slightly elevated in *cepr1-3* compared with Col-0. Only *ROXY6* expression levels were slightly reduced (Supplementary Fig. S6B). Although ROXY7, ROXY8, and ROXY9 do not appear to act downstream of CEPR1 in Col-0 under our growth conditions, we will continue to refer to ROXYs 6 to 9 as CEPDs.

### TGA1/4 amplify the expression of target genes upon treatment of N-starved seedlings with nitrate

**Figure 3.**
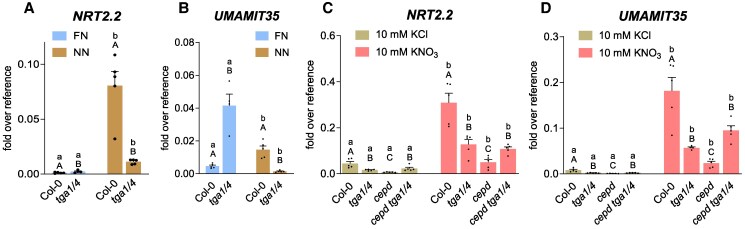
TGA1/4 can switch from a repressive into an amplifying mode. Seven-day-old seedlings grown on FN medium were transferred to NN plates. Two days later, seedlings were harvested **(A** and **B)** or treated with either 10 mm KCl or 10 mm KNO_3_ for 3 h **(C** and **D)**. Roots were collected for RNA isolation. Expression of the indicated genes was analyzed by RT-qPCR, *UBQ5* was used as a reference gene. Mean values of 4 to 5 biological replicates are shown, with 1 replicate originating from 1 plate with 10 seedlings. Error bars represent the standard error of the mean. Lowercase letters indicate statistically significant differences within the genotype between the treatments, and uppercase letters indicate significant differences within treatment between the genotypes. Statistical analyses were performed with the logarithmic values by using 2-way ANOVA and Fisher's least significant difference test (*P* < 0.05) **(A** and **B)** or Bonferroni's multiple comparisons test (*P* adj. < 0.05) **(C** and **D)**.

The strong repressive activity of Clade I TGAs was unexpected, since at least TGA1 contributes to transcriptional activation of *NRT2.2* upon binding to the respective promoter in the roots of seedlings that had been first starved for N compounds and subsequently fed with nitrate (Alvarez et al. 2014). We therefore dissected the effects of Clade I TGAs on transcription in a similar experimental set up (Fig. 3). Seedlings were first transferred from FN to no nitrogen (NN) medium, which—in contrast to the LN medium—does not contain any added N source. *NRT2.2* expression was induced substantially in Col-0. While TGA1/4 were dispensable for full *NRT2.2* induction upon transfer from FN to LN medium (Fig. 2A), *NRT2.2* was induced to much lower levels in *tga1 tga4* after transfer from FN to NN medium (Fig. 3A). *UMAMIT35* showed a different pattern: *UMAMIT35* expression was repressed by TGA1/4 in seedlings grown on FN medium (Figs. 2B and 3B), but required these transcription factors for induced expression upon transfer of seedlings to NN medium (Fig. 3B). In contrast, *UMAMIT35* expression was independent from TGA1/4 when seedlings were transferred from FN to LN medium (Fig. 2B). Thus, the composition of the medium has a profound impact on whether TGA1/4 function as activators or repressors at their target promoters.

The transcript levels of *NRT2.2* and *UMAMIT35* rose further in Col-0, *tga1 tga4*, *cepd*, and *cepd tga1 tga4* when seedlings that had been cultivated for 2 d on NN medium were treated for 3 h with 10 mm  ${\mathrm{NO}}_{3}^{-}$. This result indicates that TGA1/4- and CEPD-independent induction mechanisms mediate the response to nitrate (Fig. 3, C and D). Nitrate-induced expression levels were lower in *tga1 tga4* and *cepd tga1 tga4* than in Col-0, demonstrating that TGA1/4 amplify nitrate-responsive transcription. A comparison of transcript levels between *cepd* and *cepd tga1 tga4* seedlings suggests that CEPDs are still required to alleviate the repressive effect of TGA1/4.

### CEPDs can interfere with activating effects of TGA1/4 on gene expression

**Figure 4.**
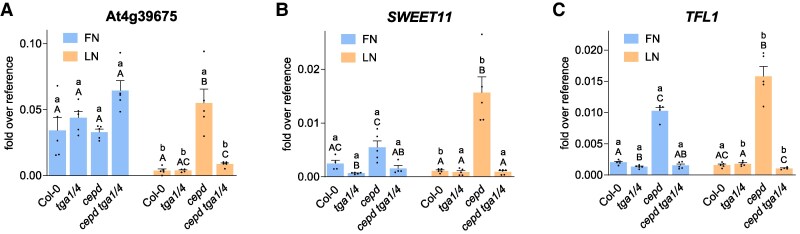
Enhanced expression in *cepd* is due to the activating effect of TGA1/4. Seven-day-old seedlings grown on FN medium were transferred to either FN or LN plates. Two days later, roots were collected for RNA isolation. Expression of **A)** At4g39675, **B)**  *SWEET11*, and **C)**  *TFL1* was analyzed by RT-qPCR, *UBQ5* was used as a reference gene. Mean values of 4 to 5 biological replicates are shown, with 1 replicate originating from 1 plate with 10 seedlings. Error bars represent the standard error of the mean. Lowercase letters indicate statistically significant differences within the genotype between the treatments, and uppercase letters indicate significant differences within treatment between the genotypes. Statistical analyses were performed by using 2-way ANOVA and Bonferroni's multiple comparisons test (*P* adj. < 0.05).

Our transcriptome analysis identified 212 genes that were negatively regulated by CEPDs (Supplementary Fig. S7). The gene with the strongest CEPD-mediated repression was At4g39675, encoding a hypothetical protein of 70 amino acids. The transcript levels of this gene were strongly downregulated under LN conditions (Fig. 4A). Under FN conditions, At4g39675 expression was not affected by the CEPD-TGA1/4 regulatory module. However, under LN conditions, the gene was highly expressed only in *cepd*, as its expression was similarly low in *cepd tga1 tga4*, Col-0, and *tga1 tga4*. Thus, LN-induced CEPDs can repress the transcriptional activation function mediated by TGA1/4, leading to lower gene expression under LN conditions.

The sucrose efflux transporter gene *SUGARS WILL EVENTUALLY BE TRANSPORTED 11* (*SWEET11*) was also expressed at lower levels upon transfer of Col-0 seedlings from FN medium to LN medium, although the effect was not nearly as strong as for At4g39675 (Fig. 4B). Based on *SWEET11* transcript levels in *tga1 tga4*, *cepd*, and *cepd tga1 tga4*, it is concluded that TGA1/4 activates *SWEET11* expression, whereas CEPDs interfere with this activation. Consequently, higher *CEPD* levels under LN conditions lead to lower *SWEET11* expression.

*TERMINAL FLOWER1* (*TFL1*) expression was strongly repressed by CEPDs under both FN and LN conditions (Fig. 4C). We detected the activating effect of TGA1/4 only in the absence of CEPDs. Due to the strong repression of *TFL1* expression by CEPDs already in place under FN conditions, LN conditions only minimally diminished its expression.

As in *cepd*, all 3 genes were more highly expressed in *cepr1-3* than in Col-0 (Supplementary Fig. S8), indicating the loss of a repressive mechanism that interferes with TGA1/4-mediated transcriptional activation of these genes in this mutant. Since overall *CEPD* transcript levels were largely unaffected in *cepr1-3* (Supplementary Fig. S6B), other gene products must be responsible for the elevated transcriptional activation of At4g39675*, SWEET11*, and *TFL1* in *cepr1-3*.

A motif mapper analysis revealed enrichment of the TACGTA motif, which is bound in vitro and in vivo by TGA1 and TGA4 (Izawa et al. 1993; Wang et al. 2019), in the promoter sequences of those genes that are upregulated in *cepd*, while the TGACG motif was slightly but significantly less frequent than expected (Supplementary Fig. S4C). The 10 most strongly upregulated genes in *cepd* relative to Col-0 all contain 1 to 3 TGA-binding sites in their promoters (Supplementary Fig. S7B), suggesting direct regulation by TGA1/4. The genes repressed by CEPDs in seedlings transferred to LN medium were enriched for the GO terms “photosynthesis,” “glucose metabolic process,” “hexose metabolic process,” “monosaccharide metabolic process,” and “generation of precursor metabolites and energy,” among others (Supplementary Fig. S7A). These GO terms are also associated with a group of genes that are more highly expressed in the roots of transgenic *35S:TGA1* seedlings (Swift et al. 2020), providing supportive evidence that TGA1 acts as an activator of these genes.

### The transcriptomes of *tga1 tga4*, *cepd*, and *cepd tga1 tga4* indicate that CEPDs act mainly through TGA1/4

We wished to explore whether TGA1, TGA4, and CEPDs act exclusively together or whether they can also control gene expression on their own. To this end, we performed RNA-seq of total RNA from the roots of wild-type, *tga1 tga4*, *cepd*, and *cepd tga1 tga4* seedlings grown on FN conditions followed by transfer to either FN or LN conditions as described above. Values are from 1 experiment with 4 independent RNAs, each originating from 1 plate with 10 seedlings.

**Figure 5.**
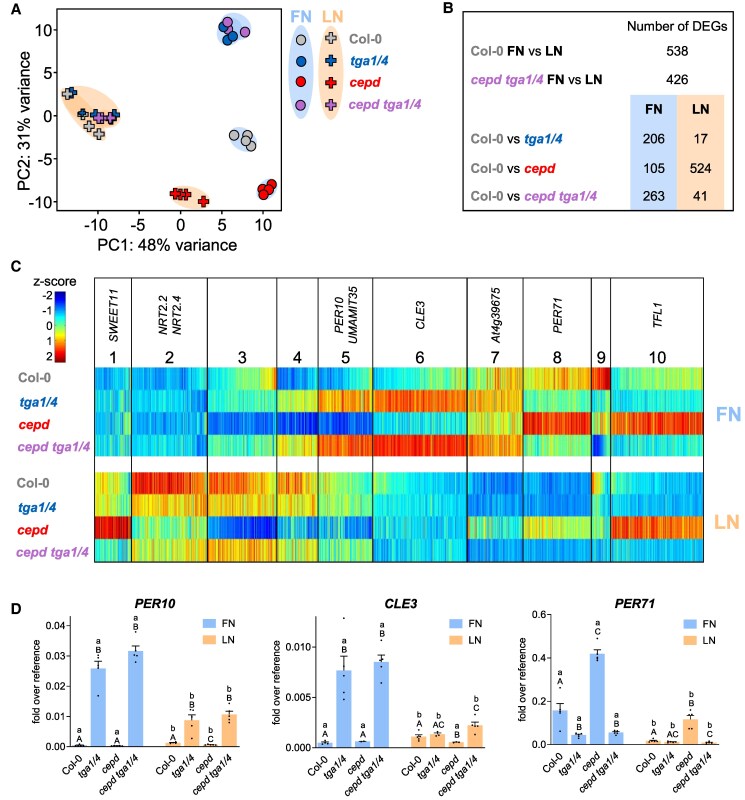
CEPDs regulate target genes mainly through TGA1/4. **A)** PCA of the normalized transcriptome data obtained by RNA-seq analysis. Symbols represent 4 biological replicates of 1 experiment with *A. thaliana* Col-0, *tga1 tga4*, *cepd*, and *cepd tga1 tga4* grown under the FN/LN regime as described in Fig. 2. **B)** Number of differentially expressed genes (log_2_FC <−1 or >1, *P* adj. < 0.05) between the indicated conditions/genotypes. **C)** Visualization of the average expression levels of 1,658 transcripts that are differentially expressed in at least 1 of the 8 samples after grouping them into 10 clusters using the MarVis software. The width of each column is proportional to the number of transcripts assigned to this cluster. Data normalization was performed using a *z*-score transformation (see color scale). **D)** Expression pattern of genes from Clusters 5, 6, and 8 obtained by RT-qPCR analysis as described in Fig. 2. Mean values of 4 to 5 biological replicates are shown, with 1 replicate originating from 1 plate with 10 seedlings. Error bars represent the standard error of the mean. Statistical analyses were performed with the logarithmic values by using 2-way ANOVA and Bonferroni's multiple comparisons test (*P* adj. < 0.05). Lowercase letters indicate statistically significant differences within the genotype between the treatments, and uppercase letters indicate significant differences within treatment between the genotypes.

A principal component analysis (PCA) of the RNA-seq data (Fig. 5A) revealed clustering of the *tga1 tga4* and *cepd tga1 tga4* samples together, under both FN and LN conditions. In fact, only 3 genes were differentially expressed between *tga1 tga4* and *cepd tga1 tga4* (log_2_FC > 1 or <−1, *P* adj. < 0.05; Supplementary Data Set 2), strongly suggesting that CEPDs act mainly, if not exclusively, through TGA1/4. Samples from Col-0, *tga1 tga4*, and *cepd tga1 tga4* clustered together under LN conditions, which is consistent with the idea that LN-induced CEPDs efficiently interfere with TGA1/4-mediated effects. Under LN conditions, only 17 genes were differentially expressed between Col-0 and *tga1 tga4* (log_2_FC > 1 or <−1, *P* adj. < 0.05), while 206 genes were differentially expressed in *tga1 tga4* compared with Col-0 under FN conditions (Fig. 5B, Supplementary Data Set 2). TGA1/4 acts as negative regulators of 80% of these genes. Even in the absence of the CEPD-TGA1/4 regulatory module in the hextuple mutant, 426 genes were differentially expressed between the 2 growth conditions, compared with 538 genes in Col-0 (Fig. 5B). Thus, CEPD-TGA1/4-independent regulatory mechanisms allow the adjustment of the transcriptome when seedlings are transferred from FN medium to LN medium.

We compiled a list of genes comprising those that are differentially expressed in Col-0 seedlings under FN vs. LN conditions and those that are differentially expressed when compared with Col-0 in at least 1 of the other 3 genotypes (log_2_FC > 1 or <−1, *P* adj. < 0.05). We subjected the resulting 1,658 genes to the MarVis suite tools (Kaever et al. 2009) to group genes with similar expression patterns into clusters (Fig. 5C, Supplementary Data Set 3)

Most of the genes belonging to Clusters 1 to 5 were induced after transfer from FN to LN conditions, with genes from Cluster 2 being most highly upregulated. Under FN conditions, expression levels of genes belonging to Cluster 2 were very similar in all 4 genotypes, most likely due to the repressive effect of ammonium and glutamine present in the FN medium (Krouk et al. 2006), which masks the influence of the CEPD-TGA1/4 regulatory module (Supplementary Fig. S5, A and B). Induction of gene expression upon transfer of seedlings to LN conditions was severely impaired in *cepd* and to a lesser extent in *tga1 tga4*, as already observed for *NRT2.4* (Fig. 2C).

Genes grouped in Cluster 3 were strongly downregulated in *cepd* under both FN and LN conditions. We only observed a small difference in the expression of these genes between Col-0 and *tga1 tga4*, suggesting efficient CEPD activity on TGA1/4-mediated repression at basal (FN) and induced (LN) CEPD levels.

Clusters 4 to 6 contained genes that were only moderately influenced by CEPDs under FN conditions and were upregulated in *tga1 tga4*, while the expression levels were similar in Col-0 and *tga1 tga4* under LN conditions. We interpret this pattern as follows: In Col-0, TGA1/4 associates with a repressive mechanism under FN conditions; as soon as the abundance of CEPDs increases upon transfer of seedlings to LN, the influence of TGA1/4 on gene expression decreases. *UMAMIT35* and *PEROXIDASE 10* (*PER10*) were among the genes in Cluster 5. The expression patterns of *PER10* (Cluster 5) and *CLAVATA3/ESR-RELATED 3* (*CLE3*, Cluster 6) demonstrated the strong repressive effect of TGA1/4 under FN conditions (Fig. 5D). Moreover, both genes were downregulated under LN conditions in *tga1 tga4*, indicating that N-regulated mechanisms can act on these promoters in the absence of TGA1/4.

Clusters 7 to 10 contained genes that are downregulated upon transfer from FN to LN conditions. Except for the genes in Cluster 9, these genes were upregulated in *cepd* due to the activating capacity of TGA1/4. A representative expression pattern of these groups of genes is illustrated by *PER71* (Cluster 8; Fig. 5D).

### Binding of TGA1 to its target promoters is independent of N supply

Since the repressive and activating functions of TGA1/4 are counteracted by increased abundance of CEPDs upon transfer of seedlings from FN to LN conditions, we tested whether inhibition of TGA1/4 activity might be due to changes in their transcript or protein levels. TGA1 protein levels were independent from the N supply and the presence of CEPDs (Supplementary Fig. S9A). These results aligned well with *TGA1* transcript levels, which were not affected by the N supply in roots (Supplementary Fig. S9B). *TGA4* transcript levels also remained unaffected by the growth conditions. The TPM values from the RNA-seq data support the notion that *TGA4* is expressed at lower levels in roots than *TGA1* (Supplementary Data Set 2).

**Figure 6.**
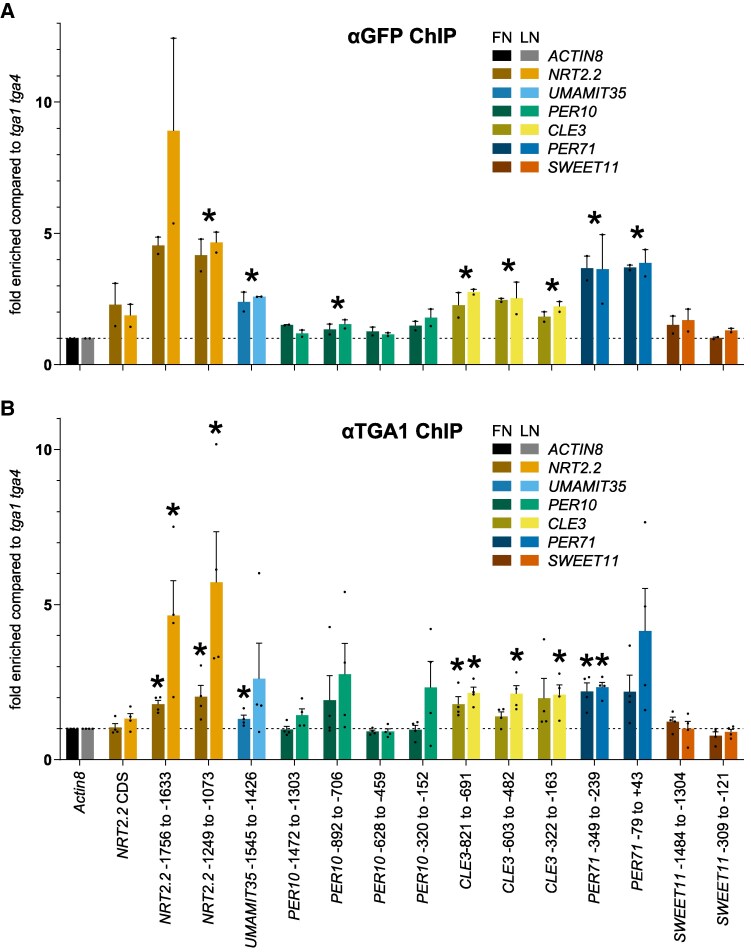
ChIP-qPCR assay documents in vivo binding of TGA1 to selected target promoters. Seedlings of *TGA1_pro_:TGA1-GFP/tga1 tga4* and *tga1 tga4*  **(A)** or Col-0 and *tga1 tga4*  **(B)** were grown under the FN/LN regime as described in Fig. 2. Roots were harvested for ChIP experiments using antibodies recognizing GFP **(A)** or TGA1 **(B)**. qPCR was performed to calculate the relative abundance of specific regions of immunoprecipitated genomic DNA. Fold enrichment when compared with *tga1 tga4* was normalized to *ACTIN8.* Numbers indicate the amplified regions with respect to the transcriptional start site. Data shown in **A)** are from 2 biological replicates, and data shown in **B)** are from 4 biological replicates. For each biological replicate, 40 plates with 10 seedlings each were harvested. Asterisks denote significant differences compared with *ACTIN8* (unpaired *t* test *P* < 0.01 in **A)**, *P* < 0.05 in **B))**. The mean of all 4 values (FN and LN) was used for the statistics of data in **A)**, and mean values from LN and FN plates were compared separately with the respective *ACTIN8* values for statistical analysis in **B)**.

To test whether TGA1 was enriched at promoters of those genes that are differentially expressed in *cepd* and *tga1 tga4*, we performed chromatin immunoprecipitation followed by quantitative PCR (ChIP-qPCR) assays using a transgenic line expressing *TGA1-GFP* under the control of the *TGA1* promoter (Wang et al. 2019; Fig. 6A). We extracted chromatin from the roots of seedlings cultivated under FN-to-FN or FN-to-LN conditions as described above with *tga1 tga4* serving as control. As reported before, when performing ChIP-qPCR analysis with an antibody directed against the N terminus of TGA1 (Alvarez et al. 2014), we detected an enrichment of *NRT2.2* promoter regions containing the TGACG sequence. We obtained similar results for the *UMAMIT35* and *CLE3* promoters. Only one fragment of the *PER10* promoter, which is strongly de-repressed in *tga1 tga4* (Fig. 5D), was slightly enriched. *PER71*, which belongs to the group of genes that is subject to CEPD-imposed repression of TGA1/4-mediated transcriptional activation (Fig. 5D), was also a direct target of TGA1; the 2 *SWEET11* promoter fragments with TGA-binding sites were not enriched in the chromatin precipitated with the anti-GFP antibody, indicating that *SWEET11* may not be a direct target of TGA1. Enrichment was independent of the growth conditions, suggesting that TGA1-GFP binds constitutively to the promoters of its target genes.

In a second series of experiments, we used a commercially available antibody generated against a TGA1 peptide for ChIP-qPCR experiments with Col-0 and *tga1 tga4* (Fig. 6B). We observed a preferential enrichment of TGA1 to target genes under LN conditions. This finding might indicate that complex formation under FN conditions leads to lower accessibility of the α TGA1 antibody to its antigen, while alteration of the complex in the presence of LN-induced CEPDs facilitates accessibility. This effect was observed not only for those promoters that are activated by growth on LN medium, but also for *PER71*, which is downregulated on LN medium due to CEPD-mediated repression. In the case of *CLE3*, which is not as strongly regulated by N supply as the other genes (Fig. 5D), the composition of the complex might be only slightly altered, resulting in similar enrichment values with chromatin from seedlings cultivated on either FN or LN medium.

In summary, our data suggest that differential expression of CEPD-TGA1/4-dependent genes under FN vs. LN conditions is most likely not due to altered TGA1/4 binding to their target promoters. Instead, we obtained evidence suggesting changes in the composition or topology of the enhanceosome in the vicinity of TGA1/4.

### The proxiome of ROXY9 includes TGAs, transcriptional co-repressors, and components of chromatin remodeling complexes

To identify ROXY9-proximal proteins, we generated transgenic Arabidopsis plants expressing the biotin ligase gene *TurboID* cloned in-frame with *ROXY9* from the *UBIQUITIN 10* (*UBQ10*) promoter in the *cepd* background. The shoot growth phenotype of the *cepd* mutant was rescued by the *TurboID-ROXY9* transgene but not by *TurboID* alone (Supplementary Fig. S10A). We worked with 2 lines with similar levels of TurboID-ROXY9 when compared with TurboID in roots (Supplementary Fig. S10B). TurboID protein abundance was not influenced by the N content of the medium in any of these transgenic lines.

We extracted proteins from the roots of biotin-treated seedlings grown first on FN medium and then transferred to either FN or LN medium. After removal of free biotin, we affinity purified biotinylated proteins using streptavidin beads (Kim et al. 2023). After tryptic in-gel digestions, peptides were fractionated and analyzed by liquid chromatography–tandem MS. The experiment was performed 4 times with independently grown seedlings. As revealed by a PCA (Supplementary Fig. S11), samples from seedlings expressing *TurboID-ROXY9* clustered away from the control line. As samples from the first replicate did not cluster with those from the other 3 replicates, we excluded this replicate from statistical analysis. In roots, we identified 187 (FN) and 247 (LN) proteins, respectively, as being enriched in the *TurboID-ROXY9* line compared with the *TurboID* control (log_2_FC >1, *P* < 0.05; Supplementary Data Set 4).

**Figure 7.**
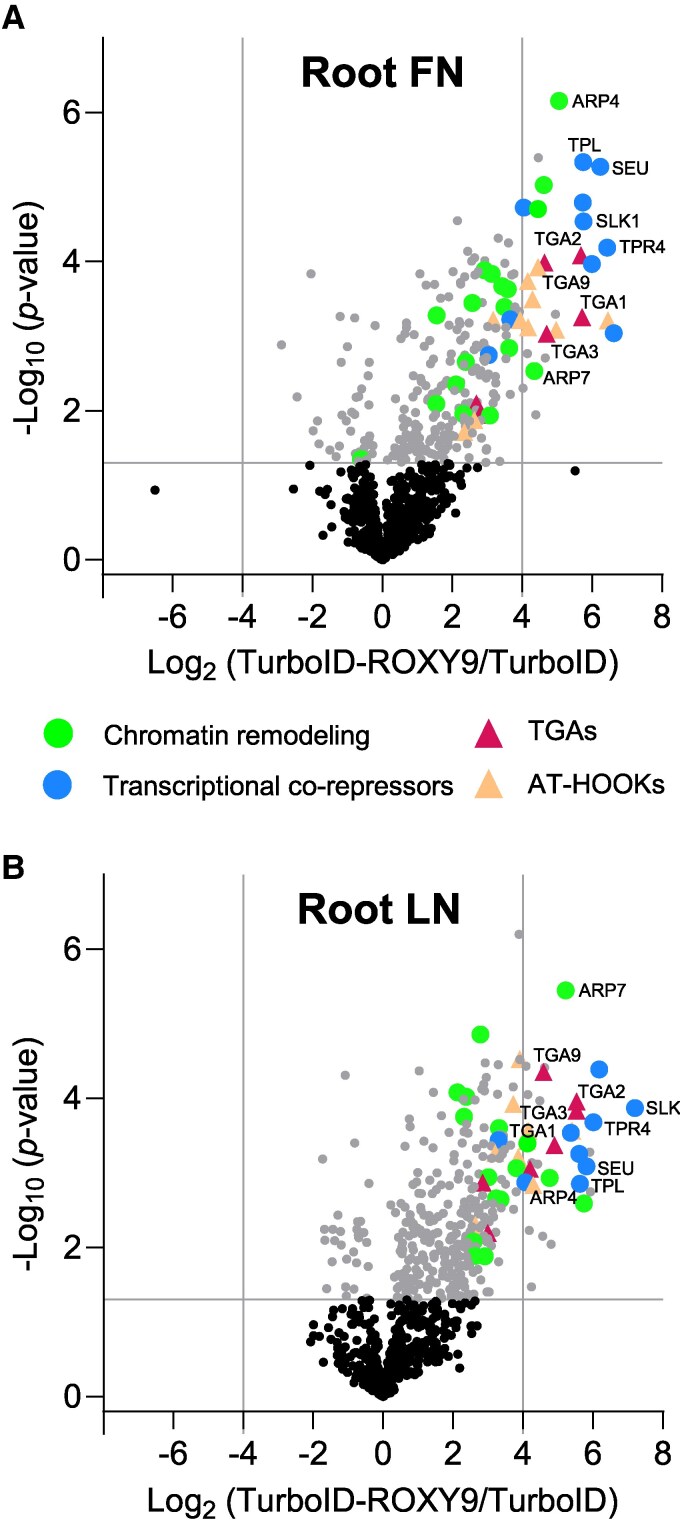
The ROXY9 proxiome is dominated by TGAs, transcriptional repressors, and components of chromatin remodeling complexes. Roots of transgenic *UBQ10:HA_2_-TurboID-ROXY9* and *UBQ10:HA_2_-TurboID* seedlings grown under the FN/FN **(A)** or FN/LN **(B)** nutrient regime were subjected to the biotin labeling protocol. Volcano plots visualize enrichment of proteins in TurboID-ROXY9 samples compared with TurboID controls. Data are derived from 3 independent experiments. Statistical analyses were performed with unpaired 2-sided Student's *t*-tests for each of the growth conditions separately. The color code in **A)** also applies to **B)**. AT-HOOKs, AT-HOOK MOTIF-CONTAINING NUCLEAR LOCALIZED (AHL) family proteins; SEU, SEUSS; SLK, SEUSS-LIKE; TPL, TOPLESS; TPR, TOPLESS-RELATED; ARP, ACTIN-RELATED PROTEIN.

Volcano plots of the FCs in protein abundance between the 2 sets of samples highlight the most highly enriched proteins (Fig. 7). In addition to the expected enrichment of peptides derived from TGA1 (Clade I), we also detected the enrichment of peptides from TGA2 (Clade II), TGA3 (Clade III), and TGA9 (Clade IV) in the *TurboID-ROXY9* lines. Moreover, the Groucho/Tup1-type co-repressors TOPLESS (TPL) and TPL-RELATED proteins TPR2, TPR4, LEUNIG (LUG), and LEUNIG-HOMOLOG (LUH) (Liu and Karmarkar 2008) were part of the ROXY9 proxiome. SEUSS (SEU) and SEU-LIKE (SLK1), which interact with LEU and LUH (Stahle et al. 2009; Shrestha et al. 2014), were detected as well. In addition to transcriptional repressors, we identified conserved components of Switch defective/sucrose nonfermentable (SWI/SNF) chromatin remodeling complexes, namely ACTIN-RELATED PROTEIN 4 (ARP4) and ARP7 (Olave et al. 2002). Conspicuously, the DNA-binding proteins AT-HOOK MOTIF-CONTAINING NUCLEAR LOCALIZED 20 (AHL20), AHL21, AHL23, and AHL27 were >16-fold more abundant in the *TurboID-ROXY9* lines compared with the control line. This analysis confirms the hypothesis that ROXY9 is recruited to chromatin through DNA-bound TGAs. There, it resides in the vicinity of transcriptional repressors and protein complexes required for chromatin opening or re-organization.

### ALWL-containing CC-type glutaredoxin-like proteins ROXYs 10 to 15 and CEPDs operate in a common regulatory network

To obtain more insight into the molecular mechanism of CEPD action, we investigated the contribution of ROXY10, ROXY11, ROXY12, ROXY13, ROXY14, and ROXY15 (ROXYs 10 to 15) in the regulation of gene expression as a function of N supply. Like *CEPD* genes, these *ROXY* genes belong to the B-γ subfamily (El Baidouri et al. 2024; Supplementary Fig. S1). They were more highly expressed in seedlings grown under FN conditions than under LN conditions (www.genevestigator.de; Scheible et al. 2004; Supplementary Fig. S3A), thus showing an opposite expression pattern to *CEPD* genes. ROXYs 11 to 15 interacted with TGA1 and TGA4 in a yeast 2-hybrid assay (Supplementary Fig. S12). They also interact with TPL and TPR2 through their C-terminal ALWL motif, and a ternary complex comprising TGA, ROXY^ALWL^, and TPL can be formed (Uhrig et al. 2017). Thus, ROXYs 10 to 15 might contribute to the regulation of N starvation–responsive genes by recruiting TPL and/or TPRs to the corresponding promoters, particularly under FN conditions. This hypothesis is attractive because ectopic expression of *ROXY14* or *ROXY15* leads to repression of *NRT2.1* and *NRT2.2* (Jung et al. 2018; Ehrary et al. 2020).

Like the *CEPD* genes, *ROXYs 10-15* were predominantly expressed in shoots, but with greater expression levels in seedlings grown on FN medium than on LN medium (Supplementary Fig. S13). *ROXYs 11-15* are arranged in tandem on Chromosome 4 within a 14 kb region, allowing the deletion of all 5 genes by CRISPR/Cas9-mediated genome editing. We also generated the *roxy10* mutant and crossed it to the *roxy11 roxy12 roxy13 roxy14 roxy15* quintuple mutant to generate the *roxy10-15* hextuple mutant.

**Figure 8.**
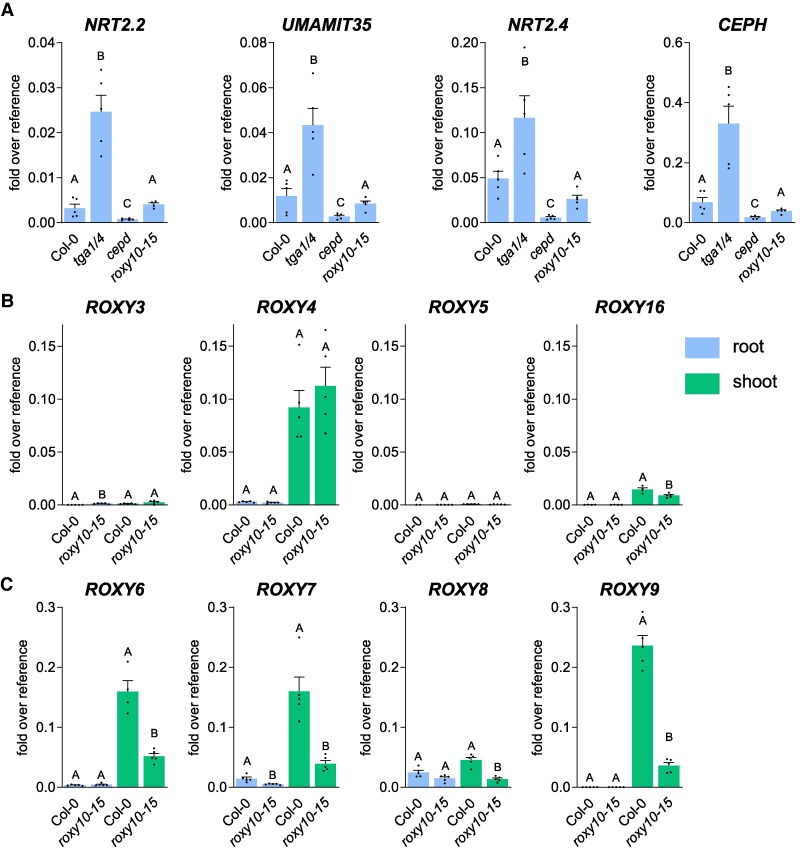
Loss of ALWL motif-containing ROXYs 10 to 15 affects *CEPD* expression in shoots but not CEPD target gene expression in roots. RNA was isolated from roots and shoots of 9-d-old seedlings grown on 10 mm  ${\mathrm{NO}}_{3}^{-}$. Expression of the indicated genes was analyzed by RT-qPCR, *UBQ5* was used as a reference gene. Mean values of 5 biological replicates are shown with 1 replicate originating from 1 plate with 10 seedlings. Error bars represent the standard error of the mean. Letters indicate statistically significant differences between the genotypes. Statistical analysis was performed with logarithmic values by 1-way ANOVA and Tukey's multiple comparisons test (*P* adj. < 0.05) in **A)** or by unpaired *t*-test (*P* < 0.05) in **B)** and **C)**.

We expected to see de-repression essentially of those genes that showed enhanced expression in *tga1 tga4.* In our first experiment, we observed a slight negative effect of ROXYs 10 to 15 on *UMAMIT35* expression, although not as pronounced as the negative effect imposed by TGA1/4 on *UMAMIT35* expression (Supplementary Fig. S14A). We observed no effect on *PER10* or *CLE3* expression, which are highly de-repressed in *tga1 tga4* (Fig. 5D). However, the effect on *UMAMIT35* expression was not robust in a second experiment (Supplementary Fig. S14B). We therefore used seedlings grown in the absence of ammonium and glutamine, which mask the TGA1/4-mediated repressive effect on *NRT2.2* and *NRT2.4* (Fig. 2, A and C, Supplementary Fig. S5, A and B). In seedlings grown on 10 mm  ${\mathrm{NO}}_{3}^{-}$, *NRT2.2*, *UMAMIT35*, *NRT2.4*, and *CEPH* expression levels were higher in *tga1 tga4* and lower in *cepd* than in Col-0. Contrary to our hypothesis, their expression levels were not affected in the *roxy10-15* hextuple mutant (Fig. 8A).

We next asked whether the function of ROXYs 10 to 15 might be compensated by potentially redundant ALWL-containing ROXYs of the B-γ group (Fig. 8B). Only *ROXY3*, which is weakly expressed, showed elevated expression levels in the roots of the *roxy10-15* hextuple mutant compared with Col-0. Notably, *CEPD* genes were less expressed in *roxy10-15* (Fig. 8C). Thus, potentially elevated expression of target genes in roots of *roxy10-15* might be compensated for by lower amounts of activating CEPDs. Auto-regulation of the ratio of CEPDs over ALWL-containing ROXYs might interfere with loss-of-function analysis. As shown for *ROXY9*, *CEPD* expression was only slightly lower in the *roxy10-15* mutant when seedlings were grown on 1 mm  ${\mathrm{NO}}_{3}^{-}$ (Supplementary Fig. S15). Consequently, expression of the target gene *UMAMIT35* was also not altered.

To obtain independent evidence for a potential function of ALWL-containing ROXYs, we analyzed the expression of their encoding genes in *cepr1-3*, the rationale being that impaired N starvation–induced expression of target genes in *cepr1-3* (Supplementary Fig. S6A) might be due to increased levels of putative repressive ROXYs. However, none of the ALWL-containing ROXYs was more highly expressed in *cepr1-3* than in Col-0 (Supplementary Fig. S16). Thus, the *cepr1-3* phenotype is not due to the upregulation of putatively repressing ALWL-containing ROXYs.

Notably, the expression of B-γ-type *ROXY* genes encoding proteins with the ALWL motif was strongly downregulated in *cepd* relative to Col-0 (Supplementary Fig. S17), suggesting that these genes are not responsible for the strong TGA1/4-mediated repression observed in *cepd*. Whether impaired expression of these B-γ-type ALWL-containing *ROXY* genes is due to the missing function of CEPDs as direct activators and/or due to N starvation in *cepd* shoots (Fig. 1) remains to be explored.

### A ROXY9 variant with a mutation in the potentially catalytic cysteine retains its function as a shoot-to-root mobile regulator of gene expression

Considering the high-sequence similarity between CEPDs and Class I glutaredoxins with oxidoreductase activity, CEPDs may function by regulating the redox state of TGA1 and TGA4 or other target proteins. According to AlphaFold predictions, the conserved catalytic CCM/LC/S motif in CEPDs is found in a position equivalent to that of the CPYC/S site in Class I glutaredoxins (Supplementary Fig. S18). In Class I glutaredoxins, only the first cysteine is crucial for enzymatic activity (Ukuwela et al. 2018). The strong phenotype of *cepd* enabled us to perform complementation assays with variants of ROXY9 in its active site. We tested protein variants with the first 2 conserved cysteines mutated to serines (CCLC to SCLC or CSLC). These mutations do not affect the interaction of ROXY9 with TGA1 or TGA4 (Li et al. 2019). In addition, we replaced the CCLC sequence with a CPYC motif. This ROXY9 variant also interacted with TGA1 and TGA4 (Supplementary Fig. S19). We cloned each coding sequence in-frame and downstream of the sequence encoding a 3xHA tag and a linker sequence, placed the entire coding sequence under the control of the *35S* promoter, and introduced each construct in *cepd*.

**Figure 9.**
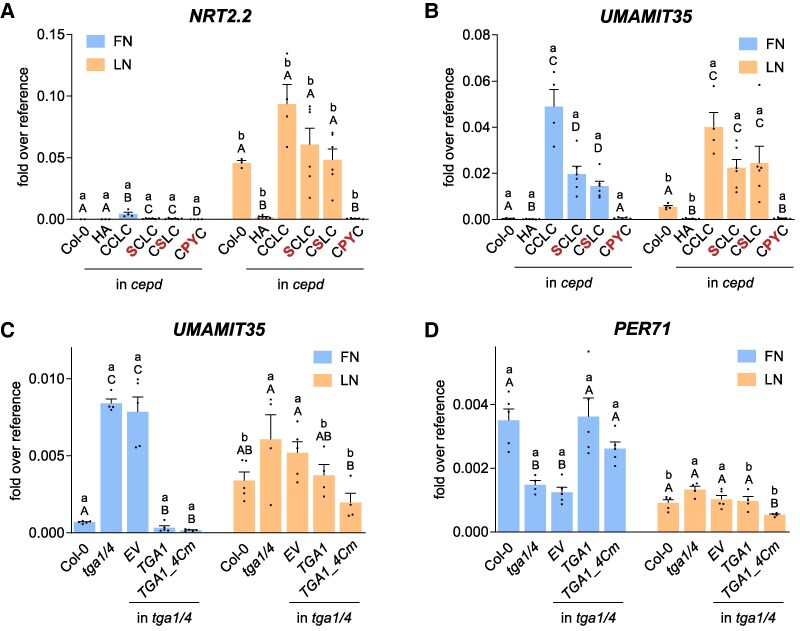
ROXY9-mediated activation of target genes is not conferred by a potential oxidoreductase activity. **A)** and **B)** Seedlings representing 4 to 5 independent lines (T2 generation) per construct, each expressing the indicated ROXY9 variants under the *CaMV 35S* promoter, were grown for 7 d on FN plates and subsequently transferred to either to FN or to LN plates. **C)** and **D)** Transgenic seedlings expressing TGA1 or a TGA1 variant with mutations in all 4 cysteines (*TGA1_4Cm*) were grown as described in **A)** and **B)**. Two days later, roots were collected for RNA isolation. Expression of the indicated genes was analyzed by RT-qPCR, and *UBQ5* was used as a reference gene. Mean values of 4 to 5 independent transgenic lines, with 1 replicate originating from 2 plates **(A** and **B)** or mean values of 4 to 5 biological replicates with 1 replicate originating from 1 plate **(C** and **D)** with 10 seedlings each are shown. **A)** to **D)** Error bars represent the standard error of the mean. Lowercase letters indicate statistically significant differences within the genotype between the treatments, and uppercase letters indicate significant differences within treatment between the genotypes. Statistical analyses were performed with the logarithmic values by using 2-way ANOVA and Bonferroni's multiple comparisons test (*P* adj. < 0.05). *NRT2.2* and *UMAMIT35* expression data for Col-0, *HA* and *ROXY9* wild-type complementation lines in **A)** and **B)** are the same as in Fig. 2, D and E.

We grew seedlings from 4 or 5 independent T2 lines per construct with similar HA-ROXY9 protein levels in their roots (Supplementary Fig. S20) under FN conditions before transferring them to FN or LN conditions and measured the expression of *NRT2.2* and *UMAMIT35* (Fig. 9A). Notably, *ROXY9^SCLC^* activated the expression of these genes, despite its encoded protein lacking the putative catalytically active cysteine. This finding strongly indicates that CEPDs do not act by altering the redox state of a potential target. ROXY9 also tolerated a cysteine-to-serine exchange at the second position (ROXY9^CSLC^), albeit this cysteine is strongly conserved in CC-type glutaredoxin-like proteins. However, changing the CCLC motif into a CPYC motif rendered the protein nonfunctional, as the corresponding transgenic lines failed to induce the expression of *NRT2.2* or *UMAMIT35*. In Class I glutaredoxins, the catalytic cysteine at the beginning of α helix 2 faces the glutathione-binding grove, while the PY sequence is localized at the surface of the protein (Supplementary Fig. S18). The PY sequence might thus disturb interactions between ROXY9 and other proteins within the enhanceosome. In contrast, mutating cysteines to serines only slightly impairs ROXY9 function.

To investigate whether the CCLC motif might be important for the transport of ROXY9 from the shoot to the root, we generated transgenic lines expressing each *ROXY9* variant under the control of the *ROXY9* promoter. We omitted a tag in order to rule out any potential artifacts. We chose primary transformants with *ROXY9* transcript levels similar to those found in Col-0 shoots for analysis. RT-qPCR analysis of expression levels of *UMAMIT35* in roots revealed that cysteine-to-serine mutations in the putative active site of ROXY9 diminished, but did not abolish, its activity (Supplementary Fig. S21), similar to our results with the *35S:HA_3_-L-ROXY9* lines (Fig. 9A). Again, ROXY9^CPYC^ was not functional. In this series of constructs, we also included a ROXY9^CCLCA^ variant, in which the conserved tyrosine flanking the active site was replaced by an alanine. Similar to the CL dipeptide, Y25 is located on the surface of the protein (Supplementary Fig. S18) and is not required for the interaction with TGA1 or TGA4 (Supplementary Fig. S19). Consistent with the notion that this part of the protein might be important for essential protein–protein interactions, the function of the ROXY9^CCLCA^ variant was severely compromised (Supplementary Fig. S21).

### Reactive cysteines in TGA1 are not involved in the regulation of CEPD-dependent genes

TGA1 and TGA4 contain redox-active cysteines (Després et al. 2003; Lindermayr et al. 2010), which might be regulated by ROXYs. To assess their functional relevance, we analyzed the induction of N starvation response genes in previously described *tga1 tga4* complementation lines with equivalent amounts of either TGA1 or a TGA1_4Cm variant in which the 4 cysteines of the protein were mutated into amino acids found in TGA2 at the equivalent position (Budimir et al. 2021; Supplementary Fig. S22). The *TGA1* constructs contain the endogenous *TGA1* promoter, 5′ and 3′ UTRs, all introns, and a sequence encoding a HA tag fused to the 5′ end of the coding region.

HA-TGA1 and HA-TGA1_4Cm were almost equally effective in repressing *UMAMIT35* expression under FN conditions (Fig. 9B), suggesting that TGA1 can function as a repressor even in its pseudo-reduced form. If inactivation of TGA1 required oxidation of cysteine residues, *UMAMIT35* induction would be impaired in the transgenic line producing the mutated protein. However, we detected clear induction, indicating that the repressive activity of TGA1 is not compromised by changes in its redox state. *PER71* belongs to the group of genes activated by TGA1/4 (Fig. 5D). HA-TGA1 and HA-TGA1_4Cm activated *PER71* expression to similar levels, and expression levels were similarly lower in the 2 complementation lines when grown under LN conditions (Fig. 9D). Thus, CEPD-mediated inactivation of TGA1 as a transcriptional activator is not due to the redox modulation of cysteines in TGA1.

## Discussion

### CEPDs act upstream of TGA1/4, but not necessarily downstream of CEPR1

Epistasis analysis helps investigate whether proteins act in a single pathway and defines their hierarchy. Here, we determined that the *cepd* phenotypes, like lower shoot weight, altered nitrate content (Fig. 1) and impaired or enhanced gene expression (Figs. 2, 4, and 5) depended on the presence of the transcription factors TGA1 and/or TGA4, demonstrating that the *tga1* and *tga4* alleles are epistatic to *cepd*. Similar studies were previously performed for ROXY1 and the TGA transcription factor PERIANTHIA (PAN; Li et al. 2009). The *roxy1* mutant produces, on average 2.5 petals, instead of the 4 petals characteristic of wild-type Arabidopsis flowers, while *pan* flowers have 5 petals (Chuang et al. 1999). Importantly, the *roxy1 pan* double mutant shows a *pan*-like phenotype with pentameric whirls. This observation indicates that the *roxy1* phenotype is due to a repressive function imposed by PAN on the initiation of petal primordia, which is counteracted by appropriate amounts of ROXY1. Likewise, antagonistic roles of the 3 redundant CC-type glutaredoxin-like proteins MSCA1, ZmGRX2, and ZmGRX5 and the TGA factor FEA4 were observed in the shoot apical meristem of maize: while the shoot apical meristems of *msca1 grx2 grx5* are smaller than wild-type meristems, those of *fea4* and *msca1 grx2 grx5 fea4* are larger. In contrast to *pan* (Chuang et al. 1999) and *fea4* (Pautler et al. 2015), both of which have aberrant meristem phenotypes, the *tga1 tga4* mutant is very similar to wild type in its nitrate content and shoot fresh weight regardless of whether plants are grown on sufficient or limiting N supply (Fig. 1). Thus, even basal levels of CEPDs in plants grown either on 10 mm  ${\mathrm{NO}}_{3}^{-}$ or on fertilized soil (Supplementary Fig. S2) are sufficient to interfere with the negative effect of TGA1/4 on the pathways controlling nitrate content and shoot fresh weight. If ROXY1 completely suppressed PAN activity, Arabidopsis plants would have pentameric flowers; likewise, if MSCA1, ZmGRX2, and ZmGRX5 completely suppressed FEA4 function, maize meristems would be larger.

CEPDs (CEPD1 [also called ROXY6] and CEPD2 [also called ROXY9]) were originally described as acting downstream of CEPR1, since induction of *ROXY6* and *ROXY9* was severely compromised in the shoots of N-starved *cepr1-1* (in Nossen; Ohkubo et al. 2017). In contrast, N starvation–induced expression of *CEPD*s was either slightly elevated (*ROXY8*, *ROXY9*) or only slightly compromised (*ROXY6*) in *cepr1-3* (in Col-0) under our growth conditions. Since expression of CEPD target genes required the CEP–CEPR1 signaling cascade (Supplementary Fig. S6), we postulate that as yet unknown gene products downstream of CEPR1 are involved in N starvation–regulated gene expression. It remains unknown whether these are synthesized in shoots or roots and whether they positively or negatively affect gene expression.

### CEPDs act at the promoters of TGA1/4 target genes

The *cepd* phenotype is associated with changes in the root transcriptome that depend on TGA1/4 (Fig. 5). ChIP-qPCR experiments (Fig. 6) suggest that TGA1/4 bind constitutively to their target promoters, independent of N supply. A very recent study provided a list of 1,105 TGA1 target loci in roots, as determined by ChIP followed by sequencing (ChIP-seq) analysis of chromatin extracted from *TGA1-GFP* seedlings grown on medium containing 10 mm  ${\mathrm{NO}}_{3}^{-}$ (Kobayashi et al. 2024). This list includes 41 of the 350 CEPD-activated genes we identified here (marked in yellow in Supplementary Data Set 1), among them *NRT2.2* and *PER71*, but not *UMAMIT35* or *CLE3*. CEPD-regulated genes without TGA1/4-binding sites might be regulated by other transcription factors, the promoters of their encoding genes being direct targets of TGA1/4. Candidates for this second wave of transcription factors are the nitrate-responsive transcription factor NIN-LIKE PROTEIN 2 (NLP2), and the transcription factors ROOT HAIR DEFECTIVE 6 (RHD6), WRKY38, and MYB121. Thirteen of the 212 genes repressed by CEPDs are present in the list of 1,105 TGA1 loci identified by Kobayashi et al. (2024) (marked in yellow in Supplementary Data Set 1), including the transcription factor gene *ETHYLENE-RESPONSE FACTOR 107* (*ERF107*). CEPDs may therefore also exert their repressive function directly at target promoters bound by TGA1/4.

For confirmation of the direct function of TGA1/4 at target promoters, promoter variants lacking the TGACG motifs should be characterized. Genome editing is preferred over promoter reporter constructs because it avoids potential position effects associated with transgene insertion across the genome. However, unexpected results may arise: for example, although in vivo binding of TGA2 to the *PATHOGENESIS RELATED 1* (*PR1*) promoter was shown by ChIP experiments (Jin et al. 2018), only deletion of a single TGA-binding site compromised promoter activity, while deletion of the 2 TGA-binding sites had no effect (Pape et al. 2010).

Constitutive expression of TurboID-ROXY9 confirmed that ROXY9 is localized in close proximity to TGA-type transcription factors. Experimental evidence for an interaction between TGA1 and ROXY9 was previously provided in yeast 2-hybrid assays and co-immunoprecipitation experiments in protoplasts (Li et al. 2019). Thus, TGA1 most likely recruits ROXY9 to target promoters. Indeed, previous super-resolution microscopy experiments have detected the strong co-localization of ROXY1 with the active form of RNA Polymerase II at euchromatin (Gutsche et al. 2017; Mass et al. 2020). In contrast to the TurboID control protein, TurboID-ROXY9 presumably resides in specific subnuclear compartments, where the relative concentration of transcriptional core components like chromatin remodeling complexes is higher than in the remaining nucleoplasm. In agreement with this idea, conserved members of the SWI/SNF chromatin remodeling complex (Huang et al. 2021; Fu et al. 2023) were significantly enriched among the peptides obtained from *TurboID-ROXY9* root extracts (Fig. 7, Supplementary Data Set 4). Proteins of the SWI/SNF complex have also been found in other proximity labeling experiments with the transcription factor BRASSINOSTEROID-INSENSITIVE 2 (BIN2; Kim et al. 2023) and the transcriptional co-activator NONEXPRESSER OF PR GENES 1 (NPR1; Powers et al. 2024). In contrast to these earlier proxiome datasets, 4 AT-hook DNA-binding proteins were enriched in *TurboID-ROXY9* root extracts. AHL family proteins bind to the minor groove of AT-rich regions, resulting in altered positioning of nucleosomes and bringing distal DNA sequences into closer proximity by forming heterotrimers and homotrimers (Zhao et al. 2013). Hence, they might contribute to CEPD-dependent gene expression.

A striking observation was the high enrichment of peptides from transcriptional co-repressors of the Groucho/Tup1 category in the proximity labeling experiment (Fig. 7, Supplementary Data Set 4). This result was unexpected, since ROXY9 is a positive regulator of the expression of 350 genes that are activated upon N starvation. TPL and related proteins have also been identified in the proximity of transcriptional activators such a BIN2 (Kim et al. 2023), FAMA (Mair et al. 2019), and NPR1 (Powers et al. 2024). These findings can be explained by the assumption that transcriptional regulation is not necessarily due to the dissociation and association of co-repressors and co-activators from and to transcription factors at promoters, but rather to dynamic processes that alter the topology of the enhanceosome. This hypothesis is supported by the observation that *Drosophila melanogaster* Groucho interacts with components of chromatin remodeling complexes (Kwong et al. 2015). In our study, co-repressors that are recruited to the promoter in a TGA1/4-dependent manner would stay within the enhanceosome even after ROXY9-TurboID-mediated transcriptional activation. An alternative explanation is that ROXY9 resides in the vicinity of transcriptional co-repressors at promoters that are repressed by CEPDs (Fig. 4), albeit ROXY9 does not contain the TPL-interacting ALWL motif (Uhrig et al. 2017).

We did not detect any major differences between the ROXY9 proxiome under FN vs. LN conditions. This result was expected, since constitutively expressed TurboID-ROXY9 most likely leads to constitutive expression of a subset of target genes under FN conditions (Fig. 2).

### Regulation of gene expression by CEPDs does not involve redox modifications

As discussed above, CEPDs most likely alter the interaction between TGA1/4 and transcriptional co-repressors or co-activators at target promoters. Since CEPDs are highly related to Class I glutaredoxins with oxidoreductase activity (Couturier et al. 2009), it seemed likely that they would regulate gene expression by controlling the redox state of reactive cysteines in their target TGAs. In maize, biochemical evidence suggests that the CC-type glutaredoxin-like proteins MSCA1, ZmGRXC2, and ZmGRXC5 negatively control the in vivo DNA-binding activity of FEA4 through keeping Cys-321 in a reduced state, thus interfering with the formation of a dimer-promoting intermolecular disulfide bridge (Yang et al. 2021). The Arabidopsis TGA factor PAN has Cys-340 in the corresponding position. Cys-340 is important for PAN function in vivo (Li et al. 2009), but does not influence the DNA-binding activity of the recombinant protein (Gutsche et al. 2017). This is different from FEA4^C321S^, which fails to bind to DNA. In *Marchantia polymorpha* TGA, Cys-231 is at the position equivalent to FEA4 Cys-321 and to PAN Cys-340. The MpTGA^C231S^ variant shows enhanced DNA-binding activity, which is less redox-sensitive than that of the wild-type protein (Gutsche et al. 2017).

In TGA1 and TGA4, Cys-260 corresponds to the cysteines discussed above. Diamide treatment of recombinant TGA1 leads to the formation of an intramolecular disulfide bridge between Cys-260 and Cys-266, which does not directly affect its DNA-binding activity (Després et al. 2003). Mutating these cysteines has no effect on in vivo TGA1 activity with respect to its activating function in the context of hyponasty and defense responses (Li et al. 2019; Budimir et al. 2021) or with respect to its regulatory function within the N signaling network (Fig. 9, C and D). The function of the conserved cysteine in different TGA factors may thus vary, suggesting that the mechanism of action of CC-type glutaredoxin-like proteins might also vary.

Still, the strict conservation of the CCM/LC/S motif (Supplementary Fig. S1) suggests a common function for maybe all CC-type glutaredoxin-like proteins. In Class I glutaredoxins, the first cysteine is strictly required for enzymatic activity (Ukuwela et al. 2018), while the last cysteine is dispensable, allowing mutations from Cys to Ser (Supplementary Fig. S1). The Cys-to-Ser exchange of the first cysteine in the active site of ROXY9 did not strongly interfere with its function, independently of the promoter driving its expression (*35S* or the *ROXY9* promoter; Fig. 9, A and B, Supplementary Fig. S21). We take this observation as strong evidence that ROXY9 does not function as an oxidoreductase when activating the expression of N starvation–induced genes. The question does remain, however, as to why Cys-to-Ser exchanges are only tolerated for the last cysteine of the CCLC motif, as in ROXY3 and ROXYs 10-17 (Supplementary Fig. S1). ROXY9^CPYC^ and ROXY^CCLCA^ were far less active than wild-type ROXY9^CCLCY^. Therefore, we postulate that essential protein–protein interactions with components of the enhanceosome are mediated by amino acids located opposite from the glutathione-binding groove at the beginning of α helix 2 (Supplementary Fig. S18). Why the first 2 cysteines are so conserved remains an open question.

### The strong TGA1/4-mediated repressive activity observed in *cepd* is not due to potentially competing ALWL-containing ROXYs 10 to 15

Since the genes encoding the TOPLESS-interacting ROXYs 10 to 15 are downregulated under limiting N supply (Supplementary Figs. S3A and S13), ROXYs 10 to 15 might modulate gene expression by competing with CEPDs, especially under sufficient N supply. However, we did not detect the same de-repression in *roxy10-15* as was observed in *tga1 tga4* (Fig. 8A). Rather, we observed lower expression levels of *CEPD* genes in the shoots of *roxy10-15* seedlings (Fig. 8C), demonstrating that ROXYs 10 to 15 are part of a regulatory network that influences *CEPD* expression in the shoot. Due to this autoregulatory mechanism, the ratio of CEPDs over potentially competing repressive ROXYs in roots (i.e. ROXYs 3 to 5, ROXY16, and ROXY17) might be similar to that in wild type, resulting in wild-type-like target gene expression. Thus, loss-of-function genetic evidence for the contribution of ALWL-containing ROXYs to the expression of CEPD-regulated genes is missing. Still, ectopic expression of *ROXY14* or *ROXY15* leads to repression of *NRT2.1* and *NRT2.2* (Jung et al. 2018; Ehrary et al. 2020).

**Figure 10.**
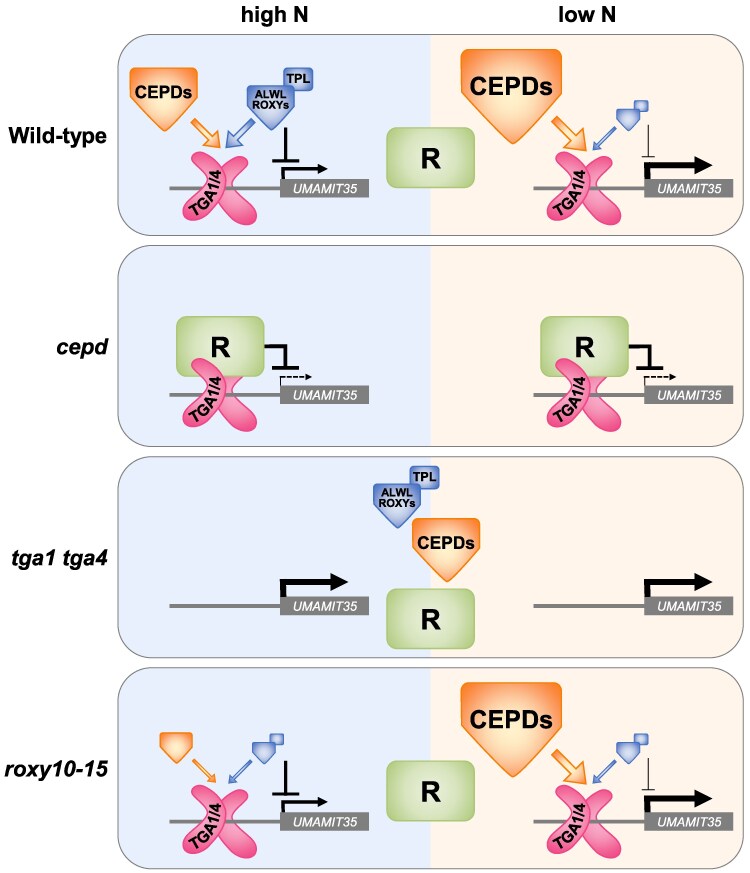
Working model illustrating the regulation of *UMAMIT35* expression by CEPDs and ALWL-containing ROXYs. In wild-type plants, CEPDs (orange symbol) and repressive ALWL-containing ROXYs associated with TOPLESS (TPL) compete for binding to TGA1/4. Their relative abundance is in favor of CEPDs under N starvation conditions leading to increased *UMAMIT35* expression. In *cepd*, expression of ALWL-containing ROXYs is severely reduced. Due to the lack of both types of ROXY proteins, an unknown repressive mechanism R is recruited to TGA1/4. Deletion of TGA1/4 leads to de-repression of the *UMAMIT35* promoter. In *roxy10-15*, expression of *CEPDs* is reduced under high N conditions maintaining the ratio of the 2 antagonistic ROXY groups which results in wild-type-like *UMAMIT35* expression. The total amount of ROXYs is still sufficient to interfere with R. Note that in the absence of any N, *UMAMIT35* expression is lower in *tga1 tga4* than in the wild type (Fig. 3).

Based on the expression pattern of *UMAMIT35*, we propose the working model displayed in Fig. 10. In the wild type, *UMAMIT3*5 expression is regulated by a combination of transcriptional regulators, among them TGA1/4. Under sufficient N supply, ALWL-containing ROXYs recruit transcriptional co-repressors like TPL and TPRs, thus dampening promoter activity. N starvation–induced CEPDs compete with N starvation–repressed ALWL-containing ROXYs for binding to TGA1/4, thereby activating promoter activity. The ratio, not the amount, of the 2 antagonistic groups of ROXYs might determine the level of gene expression, as suggested by the phenotype of the *roxy10-15* mutant. In *tga1 tga4,* regulation by ROXYs does not occur, and the remaining transcription factors activate transcription when seedlings are grown on FN medium and, slightly less strongly, when seedlings are grown on LN medium. Strikingly, this set of transcription factors is unable to maintain high transcriptional activation in the absence of TGA1/4 when seedlings are transferred to N-free medium (Fig. 3B). In *cepd*, but not in *cepd tga1 tga4*, the expression of N-responsive genes is strongly compromised. Since the expression of ALWL-containing *B-γ-*type *ROXY*s is also severely reduced in *cepd*, repression is most likely due to a yet uncharacterized repressive complex (R) that is recruited to TGA1/4. R strongly diminishes the expression of target genes under sufficient and limiting N supply, and upon N repletion (Figs. 2 and 3).

To explain the expression pattern of genes like *PER71*, whose transcriptional activation by TGA1 and TGA4 is repressed by CEPDs (Fig. 5D), we postulate that TGA1/4 are not targeted by ALWL-containing ROXYs under FN conditions and can thus function as transcriptional activators. LN-induced CEPDs would then recruit transcriptional co-repressors to TGA1 and TGA4, the interaction with these most likely being mediated by adaptor proteins.

## Materials and methods

### Plant material

All plants are in the *A. thaliana* Col-0 ecotype background, which was used as a wild-type control. References for published mutants and complementation lines are: *tga1 tga4* (Shearer et al. 2012); *TGA1_4Cm* in *tga1 tga4* (Budimir et al. 2021); *TGA1-GFP* in *tga1 tga4* (Wang et al. 2019); and *cepr1-3* (Chapman et al. 2019). Mutant alleles of *ROXY6*, *ROXY7*, *ROXY8*, *ROXY9*, and *ROXY10* and deletion of the gene cluster encoding *ROXY11*, *ROXY12*, *ROXY13*, *ROXY14*, and *ROXY15* were constructed using a CRISPR/Cas9 system as described below. To obtain the *cepd* (*roxy6-9*) quadruple mutant, *roxy6* and *roxy9* were created first as single mutants in the Col-0 background. The *roxy9* plants were transformed with the particular CRISPR/Cas9 constructs to generate *roxy7,9* and *roxy8,9* double mutants, respectively. After introducing the *roxy6* mutant allele into the *roxy7,9* plants by crossing, the resulting *roxy6,7,9* triple mutant was further crossed with *roxy8,9* enabling the identification of a *roxy6-9* (*cepd*) quadruple mutant. The *cepd tga1 tga4* hextuple mutant was obtained by crossing the *cepd* quadruple mutant with the *tga1 tga4* double mutant. For verification of all 6 mutant alleles, see Supplementary Fig. S23. Homozygous *roxy10* and *roxy11-15* plants were crossed to create the *roxy10-15* hextuple mutant. PCR analyses with primers listed in Supplementary Table S1 confirmed the genotypes of all mutants.

### Generation and analysis of transgenic plants expressing different ROXY9 variants

ROXY9 variants ROXY9_SCLC, ROXY9_CSLC, and ROXY9_CCLS have been described before (Li et al. 2019), ROXY9_CPYC and ROXY9_CCLCA were generated accordingly in pDONR207. Primers for cloning are listed in Supplementary Table S2. A linker sequence (L) coding for GGGGSGGGGSGGGGSNA was inserted upstream of the start codon of each variant. For the generation of transgenic plants expressing these L-ROXY9 variants under the control of the *CaMV 35S* promoter, the Gateway-compatible pB2HAGW7 vector was used (Li et al. 2019), resulting in *CaMV 35S*:*HA_3_-L-ROXY9* constructs (Supplementary Fig. S24). Constructs were transformed into *cepd* plants. RNA from shoots from 38 plants of the T1 generation grown on soil were tested for similar expression of the different transgenes. To avoid selection of plants with abnormally high expression of the transgenes, we exploited the fact that endogenous *ROXY9* transcript levels are elevated in *cepd*. Thus, we chose plants expressing just sufficient levels of the transgene to revert endogenous mRNA levels back to the wild-type level. Six thus selected lines of each construct were used for western blot and gene expression analysis of roots from plants harvested from FN and LN plates. Two plates with 10 plants each were used as 1 replicate.

For complementation analysis with untagged *ROXY9* variants under the control of the endogenous promoter, the genomic region from −1,135 to +2,340 bp with respect to the transcriptional start site was amplified with specific primers extended by GATEWAY© attachment sites and inserted into pDONR207 (Supplementary Table S2). Alterations in the presumed active-site motif were introduced by exchanging suitable restriction fragments derived from the respective pDONR207_ROXY9 derivatives. Sequences were introduced into pBGW (Karimi et al. 2002).

The *ROXY9* coding region was fused in frame to *HA_2_-TurboID* (Tian et al. 2021) and cloned behind the *UBQ10* promoter in the backbone of pUBQ10GW7-HA (Uhrig et al. 2017). For the sequence of the protein fusion, see Supplementary Fig. S25. *HA_2_-TurboID* cloned into the same vector context was used as control. Analysis was done with 2 homozygous *UBQ10:HA_2_-TurboID-ROXY9* lines and 1 homozygous *UBQ10:HA_2_-TurboID* control line, all of them expressing similar amounts of protein (Supplementary Fig. S10).

### Plant growth conditions

Surface-sterilized seeds were grown in vertically oriented 10 × 10 × 2 cm square plastic Petri dishes (10 seeds per plate). The basal composition of all media was 3 mm KH_2_PO_4_/K_2_HPO_4_, pH 5.8, 4 mm CaCl_2_, 1 mm MgSO_4_, 2 mm K_2_SO_4_, 3 mm MES, pH 5.8, 0.5% (w/v) sucrose, and microelements (i.e. 40 *μ*m Na_2_FeEDTA, 60 *μ*m H_3_BO_3_, 14 *μ*m MnSO_4_, 1 *μ*m ZnSO_4_, 0.6 *μ*m CuSO_4_, 0.4 *μ*m NiCl_2_, 0.3 *μ*m Na_2_MoO_4_, 20 nm CoCl_2_). For growth on full nutrition (FN), 2 mm KNO_3_, 1 mm NH_4_NO_3_, 1 mM l-glutamine, was added, for growth on low N (LN) 0.1 mm KNO_3_, 50 *μ*m NH_4_NO_3_, and 3 mm KCl was added (Scheible et al. 2004). Growth without any reduced N was done in basal media supplemented with either 10 mm KCl (NN), 1 mm KNO_3_/9 mm KCl, 3 mm KNO_3_/7 mm KCl, or 10 mm KNO_3_. Petri dishes were stored at 4 °C for 24 to 48 h with the lid facing upwards. For gene expression analysis in shoots or roots under FN and LN doses, plants were first grown for 7 days on FN medium. Subsequently, plants were either transferred to FN or to LN medium and incubated for 2 d before harvest. For gene expression analysis of plants grown on ${\mathrm{NO}}_{3}^{-}$, determination of nitrate content and shoot fresh weight, plants were grown for 21 days. Plants were cultivated at 22 °C with continuous light at a photon flux of 70 to 80 *µ*mol m^−2^ s^−1^. In Supplementary Fig. S2, *A. thaliana* seeds were cultivated on individual pots containing soil (Fruhstorfer Topferde Typ T Struktur 1 Fein) which was soaked once with 0.2% Wuxal Super (Manna, Ammerbuch-Pfäffingen, Germany). The seeds were stratified at 4 °C for 2 d after which the plants were grown in either 8 h light/16 h dark or 16 h light/8 h dark photoperiods in controlled climate chambers at 22 °C with a photon flux intensity of 100 to 120 *µ*mol m^−2^ s^−1^ during the day and 60% relative humidity.

### Genome editing

The *roxy* mutants (Supplementary Fig. S26) were generated via the CRISPR/CRISPR-associated protein 9 (CRISPR/Cas9) genome editing system. The sgRNA targeting sequences for *ROXY6*, *7*, *8*, and *9* (Supplementary Table S3) were cloned into the vector pBCsGFPEE (Nair et al. 2021) for *Agrobacterium*-mediated transformation of *Arabidopsis* plants. If the sgRNA starts with a G, no additional guanine nucleotide serving as RNA polymerase III transcription initiation site was added to the sequences. Homozygous mutants without the CRISPR/Cas9 construct to ensure genetic stability were identified as described in Nair et al. (2021).

The deletions at the *ROXY10* and *ROXY11-15* loci were obtained with T-DNA constructs encoding 2 sgRNA expression cassettes (Xing et al. 2014), respectively. The particular target sequences (Supplementary Table S3) were integrated into a PCR product by primers flanking an sgRNA scaffold, the *A. thaliana U6-26* snRNA terminator and the *U6-29* promoter sequence as shown in Supplementary Fig. S27. The PCR products were cut with *Bpi*I and *Bsa*I, respectively, and cloned into the pBCsGFPEE vector (Nair et al. 2021) cut with *Bsa*I. Since the target sequence 1 is present in both, the *ROXY11* as well as the *ROXY15* locus, sequencing results indicated that the CRISPR/Cas9 editing effect was only mediated by target 1 without modifications at the *ROXY11*-specific Target 2 site (Supplementary Fig. S26).

### Western blot, yeast 2-hybrid analysis, RNA extraction, RT-qPCR, and RNA-seq analysis

Protein extraction and western blot analysis (Budimir et al. 2021), yeast 2-hybrid analysis (Li et al. 2019), RNA extraction, cDNA preparation, and RT-qPCR analyses (Fode et al. 2008) were done as described in the respective references. Calculations were done according to the 2^−ΔCT^ method (Livak and Schmittgen 2001) using the *UBQ5* transcript as a reference (Kesarwani et al. 2007). Primers used to analyze transcript abundance are listed in Supplementary Table S4. RNA-seq and bioinformatic analyses were done as described (Ulrich et al. 2021). Please note that after the initial PCA of our first RNA-seq, we had to exclude 1 data set from Col-0 since the corresponding sample had been contaminated with leaf material. Enrichment analysis of TGA factor binding motifs within the 1,000 bp upstream regions was performed, as described previously (Berendzen et al. 2012; Zander et al. 2014) using the Cluster Analysis Real Randomization algorithm incorporated into Motif Mapper version 5.2.4.0. GO enrichment analysis was performed using the Gene Ontology Consortium web site (Ashburner et al. 2000) and the PANTHER Classification system version 17.0 (Mi et al. 2013). RNA-seq original data have been deposited in NCBI's Gene Expression Omnibus (Edgar et al. 2002) and are accessible through GEO Series accession numbers GSE282627 and GSE282484 (https://www.ncbi.nlm.nih.gov/geo/query/acc.cgi?acc=GSE282627; https://www.ncbi.nlm.nih.gov/geo/query/acc.cgi?acc=GSE282484).

### Chromatin immunoprecipitation

ChIP-qPCR was performed according to the previously published protocol (Cortijo et al. 2018). For each biological replicate, roots were harvested from ∼500 to ∼600 seedlings (520 to 900 mg), corresponding to 50 to 60 plates per sample. Sonication of the chromatin was done with the BioruptorPlus device (Diagenode) using 14 × 30 s on/off cycles with a 10 min interruption after 7 cycles. Chromatin immunoprecipitation was done on Dynabeads (50% ProteinA, 50% ProteinG; Invitrogen) loaded with either antiGFPab290 (Abcam) or anti-TGA1 (Agrisera). Quantification of enriched DNA was done by qPCR (for primers, see Supplementary File 1). Fold enrichment when compared with *tga1 tga4* was normalized to *ACTIN8*.

### Proximity labeling

Roots were obtained from transgenic *UBQ10:HA_2_-TurboID-ROXY9* and *UBQ10:HA_2_-TurboID* plants grown on vertically oriented agar plates under the FN/FN or FN/LN regimes described above. Plates were oriented horizontally and 20 mL of 150 *µ*m biotin in 10 mm MgCl_2_ was added to each plate so that seedlings were submerged four 1 h. For 1 biological replicate, roots from 13 plates (10 seedlings per plate) were harvested into a 50 mL Falcon tube filled with water. Two experiments were performed with *TurboID-ROXY9* #14-7 and 2 experiments were performed with *TurboID-ROXY9* #19-4. *TurboID #*6-3 was used in all 4 replications. Seedlings were washed 2 times with water, residual water was removed with a paper towel, and the material was frozen in liquid nitrogen and stored at −70 °C. Further processing was done according to Kim et al. (2023) using Streptavidin Dynabeads M-280 (Invitrogen; 50 *µ*L beads/1 g ground material). Biotinylated proteins were eluted twice at 99 °C for 5 min in 30 *µ*L rapid protein extraction buffer (RPB) (100 mm Tris, pH 6.8, 4% SDS, 20% glycerol, 0.02% bromophenol blue, 200 mm DTT). Subsequent analysis was done according to Schmitt et al. (2021) with minor modifications. Samples were subjected to SDS–PAGE for in-gel digest of proteins with trypsin. Peptides were purified using C18 (#2215, 3 m) stop and go extraction (stage) tips. Peptides were dried, dissolved in 20 *µ*L sample buffer (2% acetonitrile, 0.1% formic acid), and analyzed with LC–MS/MS using an UltiMate 3000 RSLCnano system coupled to a Q Exactive HF mass spectrometer (all Thermo Fisher Scientific; Schmitt et al. 2021). Full scans were acquired within a mass range of 300 to 1,650 *m*/*z* at a resolution of 30,000 followed by data-dependent top 10 HCD fragmentation at a resolution of 15,000. Dynamic exclusion was enabled. The XCalibur 4.0 software was used for method programming and data acquisition (Thermo Fisher Scientific). The MS proteomics data have been deposited to the ProteomeXchange Consortium via the PRIDE partner repository with the dataset identifier PXD058335 (Perez-Riverol et al. 2022).

### MS data analysis

Protein identification and label-free quantification (LFQ) were performed with MaxQuant version 2.5.2.0 (Tyanova et al. 2016a) using default settings. Peptides were searched against the Araport 11 protein database containing a total of 27,562 entries (Araport11_pep_20220914_representative_gene_model) with the addition of the HA_2_-TurboID protein. Filtering and statistical analyses were done with Perseus (Tyanova et al. 2016b). The “proteinGroups.txt” output file from MaxQuant was imported into Perseus, and LFQ intensities were selected as main columns. Proteins categorized as “contaminants,” “reverse,” and “only identified by site” were filtered out. The PCA was performed with log_2_-transformed LFQ values after imputation of missing values from normal distribution using Perseus default parameters. The exported projections of PC1 and PC2 were used to generate a PCA plot with GraphPad Prism 10 (GraphPad Software, Inc., San Diego, CA, USA). To identify proteins enriched in TurboID-ROXY9 samples compared with TurboID controls, an unpaired 2-sided Student's *t*-test was performed in Perseus for each of the analyzed tissues/growth conditions. LFQ values were log_2_-transformed and proteins that were not identified or quantified in all replicates of at least 1 genotype were removed. Missing values were imputed from normal distribution using Perseus default parameters. The obtained *t*-test −log_10_  *P*-values were plotted vs. the differences to generate volcano plots with GraphPad Prism 10.

### Determination of N and nitrate content

Nitrate content determination was based on a spectroscopic method as described (Hachiya and Okamoto 2017). Briefly, 20 to 100 mg material per replicate was frozen in liquid N_2_ and homogenized in a ball mill. Samples were separated. For nitrate content determination, 10 volumes of water were added, and samples were incubated for 20 min in a 100 °C water bath. Cooled tubes were centrifuged for 10 min at 20,400*×g* and 10 *µ*L of the supernatants were incubated with 80 *µ*L of 0.05% (w/v) salicylic acid in sulfuric acid for 20 min at room temperature. One milliliter of 8% NaOH in water was added, and the samples were vortexed until the contents became clear. Absorbance was measured at 410 nm along with a calibration curve. For total N determination, samples were lyophilized and analyzed by an elemental analyzer (EA-IRMS).

### Statistical analysis

GraphPad Prism 10 was used to conduct statistical analysis. Detailed results are shown in Supplementary Data Set 5.

### Accession numbers

Sequence data from this article can be found in the Arabidopsis Genome Initiative or GenBank/EMBL databases under the following accession numbers: *CEPH* (At4g32950); *CLE3* (At1g06225); *NRT2.2* (At1g08100); *NRT2.4* (At5g60770); *PER10* (At1g49570); *PER71* (At5g64120); *ROXY3/GRXS10* (AT3g21460); *ROXY4/GRXC11* (AT3g62950); *ROXY5/GRXC12* (AT2g47870); *ROXY6/GRXS11/CEPD1* (AT1g06830); *ROXY7/GRXS9/CEPDL2* (At2g30540); *ROXY8/GRXC14/CEPDL1* (At3g62960); *ROXY9/GRXC13/CEPD2* (At2g47880); *ROXY10/GRXS2* (At5g18600); *ROXY11/GRXS3* (At4g15700); *ROXY12/GRXS5* (At4g15690); *ROXY13/GRXS4* (At4g15680); *ROXY14/GRXS7* (At4g15670); *ROXY15/GRXS8* (At4g15660); *ROXY16/GRXS1* (At1g03020); *SWEET11* (At3g48740); *TFL1* (At5g03840); *TGA1* (At5g65210); *TGA4* (At5g10030); *UBQ5* (At3g62250); and *UMAMIT35* (At1g60050).

## Supplementary Material

koaf038_Supplementary_Data

## Data Availability

Data are incorporated into the article and its online supplementary material. Data derived from transcriptomics and MS analyses are available in the respective repositories as indicated in the Material and Method section.

## Supplementary data

The following materials are available in the online version of this article.

**Supplementary Figure S1.** Alignment of active-site motifs in glutaredoxin and glutaredoxin-like proteins in *A. thaliana*.

**Supplementary Figure S2.** Shoot fresh weight of soil-grown Col-0, *tga1 tga4*, *cepd*, and *cepd tga1 tga4* plants.

**Supplementary Figure S3.** Transcript levels of *CEPDs* in shoots and roots of Col-0 grown under full and limiting N supply.

**Supplementary Figure S4.** GO-term and motif mapper analysis of CEPD-regulated genes.

**Supplementary Figure S5.** Effect of ammonium and glutamine on the expression pattern of *NRT2.2*.

**Supplementary Figure S6.** Reduced expression of N starvation–induced genes in *cepr1-3* is not associated with reduced expression of *CEPDs*.

**Supplementary Figure S7.** GO-term analysis of 212 genes that are higher expressed in *cepd*.

**Supplementary Figure S8.** Transcript levels of CEPD-repressed genes in roots of Col-0 and *cepr1-3* grown under full and limiting N supply.

**Supplementary Figure S9.** TGA1 protein and *TGA1* and *TGA4* transcript levels in plants grown under full and limiting N supply.

**Supplementary Figure S10.** Characterization of TurboID-ROXY9 lines.

**Supplementary Figure S11.** PCA of proteins in TurboID-ROXY9 samples compared with TurboID controls.

**Supplementary Figure S12.** Yeast 2-hybrid analysis with TGAs and ROXYs.

**Supplementary Figure S13.** Transcript levels of *ROXYs 10-15* in roots and shoots of Col-0 grown under full and limiting N supply.

**Supplementary Figure S14.**  *UMAMIT35, PER10,* and *CLE3* transcript levels in roots of Col-0 and *roxy10-15* plants grown under full nitrogen supply.

**Supplementary Figure S15.** Transcript levels of *ROXY9* and *UMAMIT35* in shoots and roots of plants cultivated under sufficient or limiting N conditions.

**Supplementary Figure S16.** Expression of *ROXYs 1-21* in shoots of Col-0 and *cepr1-3* after transfer from FN to LN medium.

**Supplementary Figure S17.**  *B-γ ALWL ROXY* expression in *cepd*.

**Supplementary Figure S18.** Structural analysis of ROXY9 and GRXC2.

**Supplementary Figure S19.** Yeast 2-hybrid analysis with TGA1/4 and ROXY9 active-site mutants.

**Supplementary Figure S20.** Western blot analysis of transgenic *cepd* plants expressing *ROXY9* or *ROXY9* active-site mutants under the control of the *CaMV 35S* promoter.

**Supplementary Figure S21.** Analysis of transgenic *cepd* plants expressing *ROXY9* or *ROXY9* active-site variants under the control of the *ROXY9* promoter.

**Supplementary Figure S22.** Characterization of TGA1 complementation lines.

**Supplementary Figure S23.** Confirmation of the 6 mutant alleles in *cepd tga1 tga4*.

**Supplementary Figure S24.** Sequence of the *CaMV 35S:HA_3_-L-ROXY9* construct.

**Supplementary Figure S25.** Sequence of the *UBQ10:HA_2_-TurboID-ROXY9* construct.

**Supplementary Figure S26.** CRISPR/Cas9-based genome editing of *ROXY* genes.

**Supplementary Figure S27.** PCR products were used for CRISPR/Cas9-based genome editing of the *ROXY10* and *ROXY11-15* loci.

**Supplementary Data Set 1.** Differentially expressed genes (roots; Col-0 vs. *cepd*, LN).

**Supplementary Data Set 2.** Differentially expressed genes (roots; Col-0, *tga1 tga4, cepd*, *cepd tga1 tga4,* FN and LN).

**Supplementary Data Set 3.** Cluster analysis of differentially expressed genes (Col-0, *tga1 tga4*, *cepd*, *cepd tga1 tga4*, FN and LN).

**Supplementary Data Set 4.** Enriched proteins in TurboID-ROXY9 samples compared with TurboID controls.

**Supplementary Data Set 5.** Results of statistical analyses.

**Supplementary Table S1.** Primers for genotyping.

**Supplementary Table S2.** Primers for cloning.

**Supplementary Table S3.** Single-guide RNA (sgRNA) targeting sequences.

**Supplementary Table S4.** Primers for RT-qPCR.

**Supplementary File 1.** Promoter and primer sequences for ChIP analyses.

## Dive Curated Terms

The following phenotypic, genotypic, and functional terms are of significance to the work described in this paper:

LUH Gramene: AT2G32700
LUH Araport: AT2G32700

SLK1 Gramene: AT4G25520
SLK1 Araport: AT4G25520

WRKY38 Gramene: AT5G22570
WRKY38 Araport: AT5G22570

BIN2 Gramene: AT4G18710
BIN2 Araport: AT4G18710

UBQ10 Gramene: AT4G05320
UBQ10 Araport: AT4G05320

TGA4 Gramene: At5g10030
TGA4 Araport: At5g10030

ROXY9 Gramene: At2g47880
ROXY9 Araport: At2g47880

ROXY1 Gramene: At3g02000
ROXY1 Araport: At3g02000

TPR2 Gramene: AT3G16830
TPR2 Araport: AT3G16830

NPR1 Gramene: AT1G64280
NPR1 Araport: AT1G64280

TGA3 Gramene: AT1G22070
TGA3 Araport: AT1G22070

PAN Gramene: AT1G68640
PAN Araport: AT1G68640

PR1 Gramene: At2g14610
PR1 Araport: At2g14610

TGA1 Gramene: At5g62510
TGA1 Araport: At5g62510

CEPR1 Gramene: AT5G49660
CEPR1 Araport: AT5G49660

nitrate CHEBI: CHEBI:17632

RHD6 Gramene: ROOT HAIR DEFECTIVE SIX
RHD6 Araport: ROOT HAIR DEFECTIVE SIX

CEPR2 Gramene: AT2G35612
CEPR2 Araport: AT2G35612
