# Supplementary material for: Redox-inactive CC-type glutaredoxins interfere with TGA transcription factor–dependent repression of target promoters in roots
Source: Plant Cell. 2025 Mar 7;37(3):koaf038. doi: 10.1093/plcell/koaf038 (PMC11887855; doi:10.1093/plcell/koaf038)
Supplement: koaf038_Supplementary_Data [file koaf038_supplementary_data.zip › TPC2024RA11307DR1_Supplementary_Figures_Tables_and_Files.pdf]

Supplementary Fig. S1

Class I

	active site
GRXC1	...CGYC Q...
GRXC2	...CPYC V...
GRXC3	...CPYC L...
GRXC4	...CPYC K...
GRXC5	...CSYC T...
GRXS12	...CSYS S...

Class II

	active site
GRXS14	...CGFS N...
GRXS15	...CGFS S...
GRXS16	...CGFS Q...
GRXS17.1	...CGFS R...
GRXS17.2	...CGFS G...
GRXS17.3	...CGFS S...

Class III (CC-type)

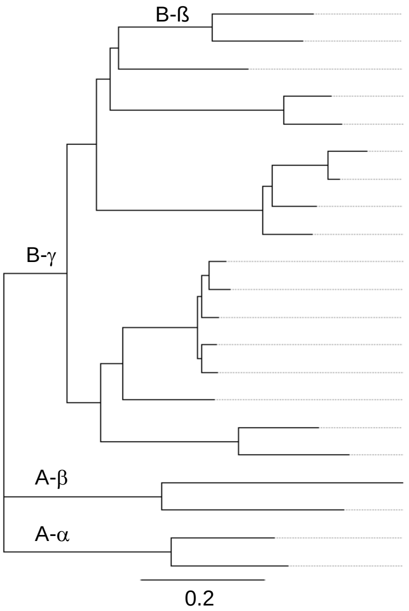

	active site	C-terminus	
ROXY1	...CCMC H...	ALWL	
ROXY2	...CCMC H...	ALWL	
ROXY3	...CCMS H...	ALWL	
ROXY4	...CCMC H...	AIWL	
ROXY5	...CCMC H...	AIWL	
ROXY8	...CCLC Y...	SFHN	CEPDs
ROXY9	...CCLC Y...	SILY	CEPDs
ROXY6	...CCLS Y...	--LC	CEPDs
ROXY7	...CCMS Y...	ANLC	CEPDs
ROXY14	...CCMS H...	ALWL	
ROXY15	...CCMS H...	ALWL	
ROXY13	...CCMS H...	ALWL	
ROXY11	...CCMS H...	ALWL	
ROXY12	...CCMS H...	ALWL	
ROXY10	...CCMS H...	ALWV	
ROXY16	...CCMS H...	AIWI	
ROXY17	...CCMS H...	AIWV	
ROXY18	...CCLG H...	ALWL	
ROXY19	...CCMC H...	ALWL	
ROXY20	...CCMC H...	ALWA	
ROXY21	...CCMC H...	ALWV	

Fig. S1 Alignment of active site motifs in glutaredoxin and glutaredoxin-like proteins in *Arabidopsis thaliana*. ROXY sequences are arranged according to the neighbor-joining tree based on the Jukes-Cantor genetic distance model. The scale bar indicates the number of amino acid substitutions per site. The classification into subfamilies (A-α, A-β, B-β, B-γ) is from El Baidouri *et al*., 2024.

Fig. S1 supports the Introduction.

Supplementary Fig. S2

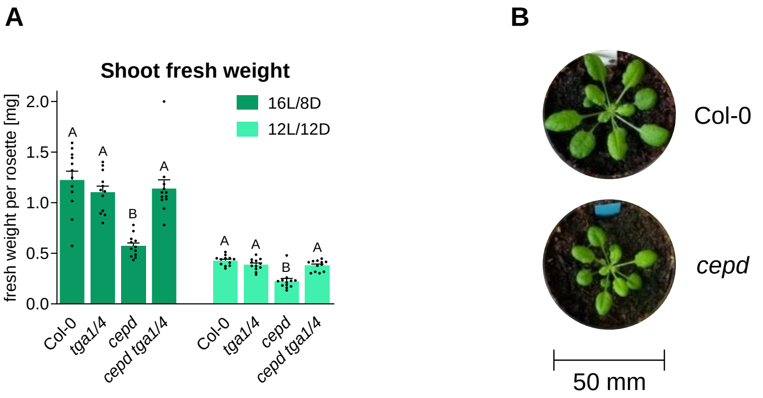

Fig. S2 Shoot fresh weight of soil-grown Col-0, *tga1 tga4*, *cepd* and *cepd tga1 tga4* plants. A) Plants were grown for 4 weeks under a 16 h light (L)/8 h dark D or a 12L/12D regime. Mean values of 12 biological replicates per genotype are shown. Error bars represent the standard error of the mean. Letters indicate statistically significant differences between the genotypes. Statistical analysis was performed by one-way ANOVA and Tukey's multiple comparisons test (p adj. < 0.05). **B)** Picture of representative plants grown for four weeks under a 12L/12D regime.

Fig. S2 supports Fig. 1.

Supplementary Fig. S3

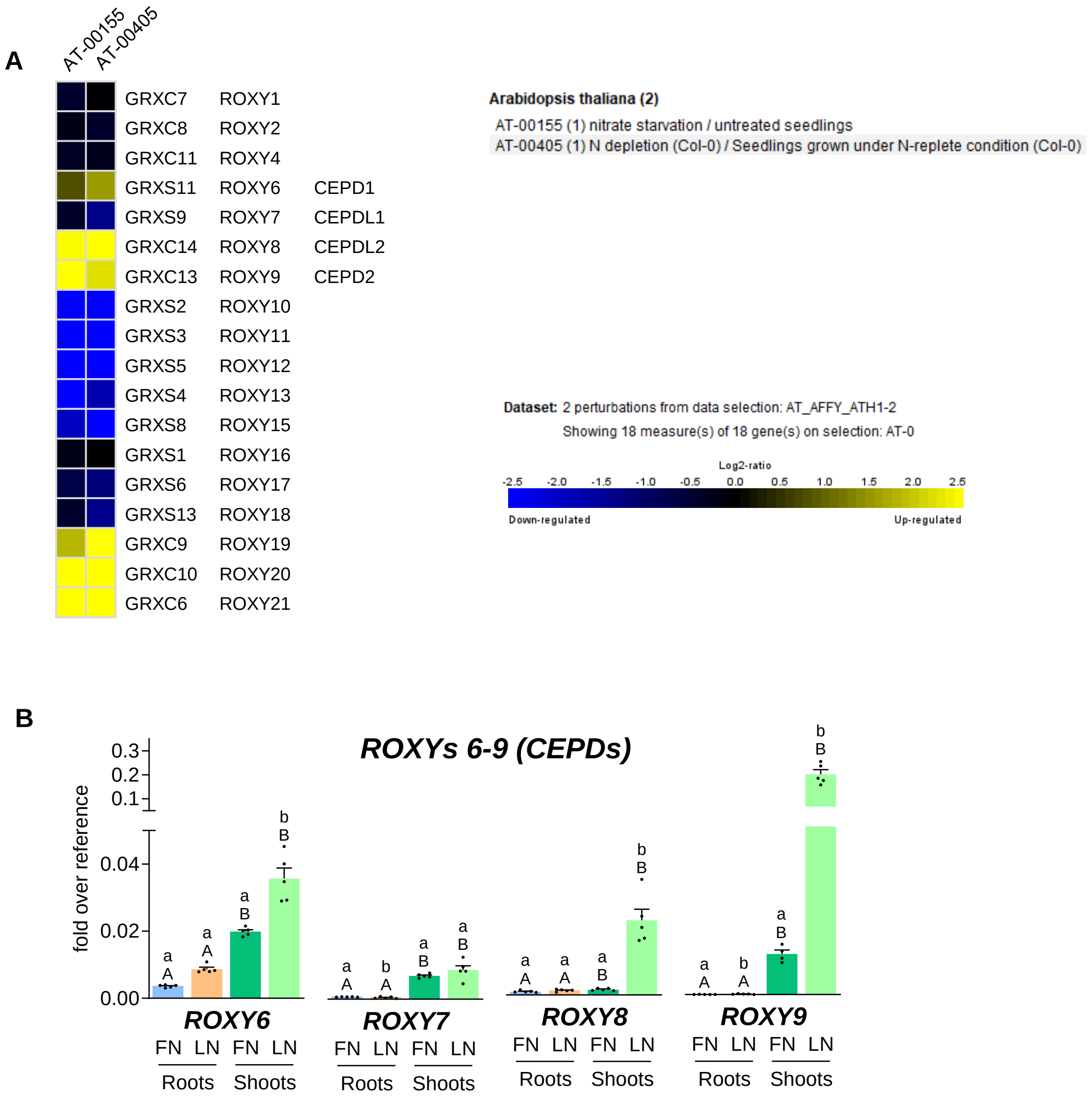

Fig. S3 Transcript levels of *CEPD*s in shoots and roots of Col-0 grown under full and limiting N supply. A) Expression levels of *Arabidopsis thaliana* (Col-0) CC-type glutaredoxins according to www.genevestigator.de. Seven-day-old seedlings grown in liquid full nutrition (FN) medium under constant light were transferred to either FN or low nitrogen (LN) medium. Two days later, seedlings were collected for transcriptome analysis (Scheible *et al*., 2004). **B)** Seedlings were grown as in **A),** but on agar-solidified medium. Shoots and roots were collected separately for RNA isolation. Expression of the indicated genes was analyzed by RT-qPCR, *UBQ5* was used as a reference gene. Mean values of four to five biological replicates are shown, with one replicate originating from one plate with 10 seedlings. Error bars represent the standard error of the mean. Lowercase letters indicate statistically significant differences within the tissue between the conditions, uppercase letters indicate significant differences within treatment between the tissues. Statistical analyses were performed with the logarithmic values by using two-way ANOVA and Fisher's Least Significant Difference test ($p < 0.05$). Fig. S3 supports Fig. 2.

Supplementary Fig. S4

A

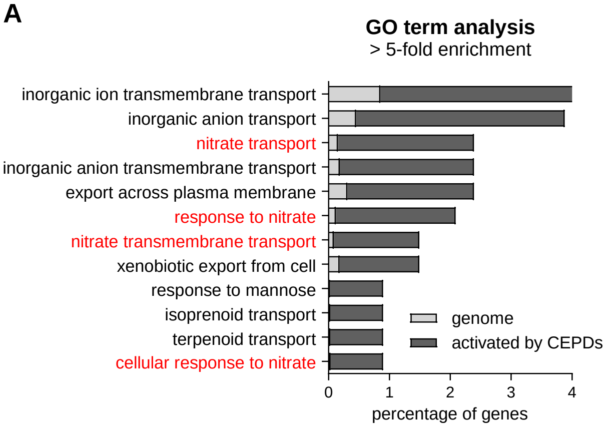

B

	TPM Col-0	TPM *cepd*	log_2 FC	TGA binding
NRT2.2	127.66	3.31	4.59	**5**
UMAMIT35	19.19	0.57	4.47	**4**
NRT2.4	121.67	4.59	4.26	**2**
ATL7	5.83	0.15	3.55	**-**
AMT1-5	9.87	0.34	3.51	**3**
RL3	15.23	0.92	3.35	**1**
NLP3	22.47	1.89	3.34	**-**
OCT1	11.02	0.82	3.32	**3**
CEPH	140.96	10.42	3.22	**4**
At4G37220	19.44	1.57	3.19	**-**

C

	350 *cepd* down	350 genome	*p*-value	enrichment	212 *cepd* up	212 genome	*p*-value	enrichment
**TGACG**	335	298.2	0.0192	**1.12**	159	183.7	-0.0510	**0.87**
**TGACGTA**	9	4.7	0.0294	**1.93**	1	2.8	-0.1539	**0.36**
**TACGTA**	78	62.4	0.0359	**1.25**	48	38.0	0.0643	**1.26**

Fig. S4 GO term and motif mapper analysis of CEPD-regulated genes. A) Gene Ontology (GO) term analysis (biological processes) of 350 genes, which are higher expressed (log_2 FC > 1, *p* adj. < 0.05) in roots of 9-day-old Col-0 seedlings compared to *cepd* after two days of cultivation on LN (low nitrogen) medium. GO terms related to nitrogen are displayed in red. Bars represent the percentage of genes found per GO term in the group of 350 genes (dark grey) and the percentage of genes representing the respective GO term found within the Arabidopsis genome (light grey). GO terms with > 5-fold enrichment against the genome are shown. Statistical analysis was performed using Fisher's Exact test and False Discovery Rate (FDR) < 0.05. **B)** List of the ten most highly differentially expressed genes. The number of TGA binding sites (TGACG and TACGTA) was counted in the region 2 kb upstream of the transcriptional start site. TPM: transcripts per million. **C)** Motif Mapper *cis*-element analysis. Counts of motifs in sequences 1 kb upstream of the transcriptional start site in 350 or 212 genes higher or lower expressed in Col-0 as compared to *cepd,* respectively, and in 350 or 212 genes randomly picked from the genome are indicated.
Fig. S4 supports Fig. 2.

Supplementary Fig. S5

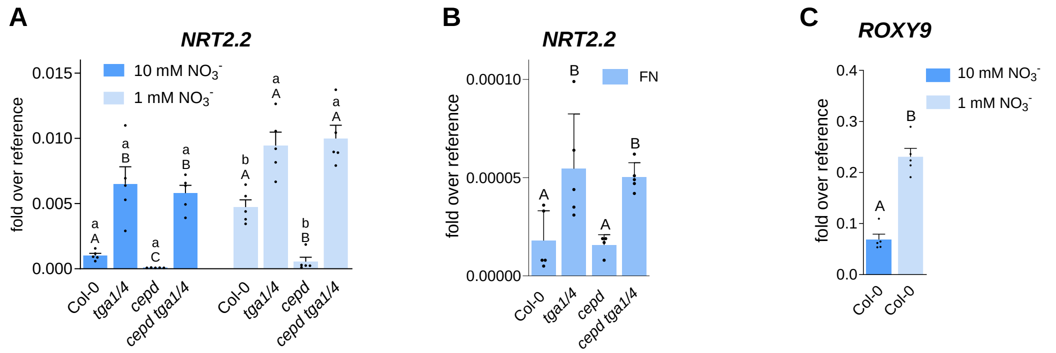

Fig. S5 Effect of ammonium and glutamine on the expression pattern of *NRT2.2*. A) and **C)** Wild-type (Col-0), *tga1 tga4, cepd* and *cepd tga1 tga4* plants were grown on medium containing 1 or 10 mM NO_3^- under constant light (70 µmol photons s^{-1} m^{-2}). After 21 days, shoots and roots were collected separately for determination of transcript levels by RT-qPCR. **B)** 7-day-old seedlings of the indicated genotypes grown on full nitrogen (FN) medium were transferred to FN for 2d and roots were collected for determination of transcript levels by RT-qPCR. **A)** to **C)** Mean values of five biological replicates are shown, with one replicate originating from one plate with 10 seedlings. Error bars represent the standard error of the mean. Statistical analyses were performed with logarithmic values by using two-way ANOVA and Bonferroni's multiple comparisons test (*p* adj. < 0.05) with lowercase letters indicating statistically significant differences within the genotype between the treatments and uppercase letters indicating significant differences within treatment between the genotypes in **A)**, one-way ANOVA and Tukey's multiple comparisons test (*p* adj. < 0.05) with uppercase letters indicating significant differences between genotypes in **B)**, or unpaired t-test (*p* < 0.05) with uppercase letters indicating significant differences between the treatments in **C)**.
Fig. S5 supports Fig. 2.
Fig. S5B contains data of Fig. 2A.

Supplementary Fig. S6

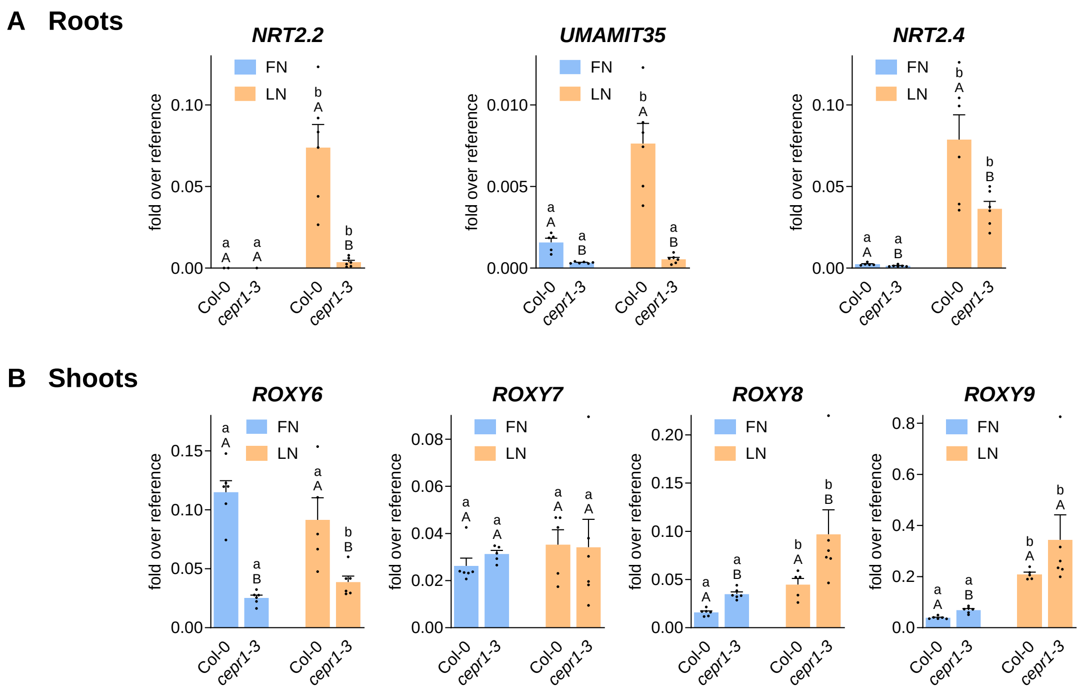

Fig. S6 Reduced expression of N starvation-induced genes in *cepr1-3* is not associated with reduced expression of *CEPDs*. 7-day-old seedlings grown on full nitrogen (FN) medium were transferred to either FN or low nitrogen (LN) plates. Two days later, **A)** roots and **B)** shoots were collected for RNA isolation. Expression of the indicated genes was analyzed by RT-qPCR, *UBQ5* was used as a reference gene. Mean values of four to five biological replicates are shown with one replicate originating from one plate with 10 seedlings. Error bars represent the standard error of the mean. Lowercase letters indicate statistically significant differences within the genotype between the treatments, uppercase letters indicate significant differences within treatment between the genotypes. Statistical analyses were performed with logarithmic values by using two-way ANOVA and Fisher's Least Significant Difference test ($p < 0.05$).
Fig. S6 supports Fig. 2.

Supplementary Fig. S7

A

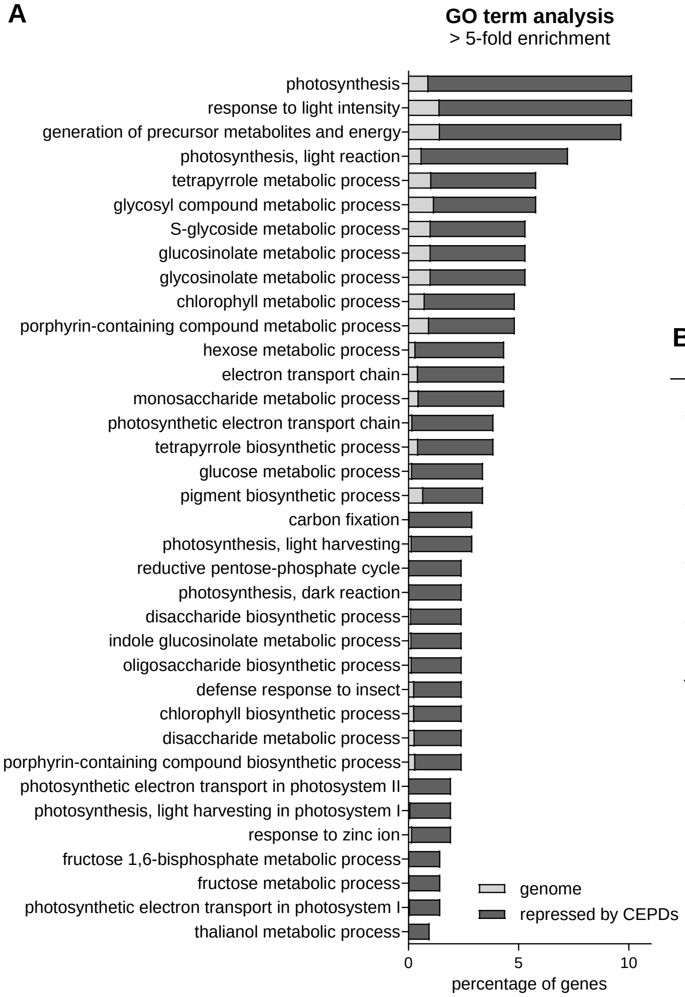

B

	TPM Col-0	TPM *cepd*	log_2 FC	TGA binding
AT4G39675	1.98	38.79	-3.16	2
SWEET11	0.18	6.03	-2.70	3
TFL1	2.10	17.87	-2.49	2
AT1G52100	0.21	2.02	-2.22	2
GDPD3	0.07	1.19	-2.22	1
AT4G29905	39.61	222.61	-2.19	2
IGMT1	4.50	23.42	-2.18	3
AT1G49500	7.34	38.52	-2.14	3
PER71	31.68	179.93	-2.09	2
AIR1	26.69	152.68	-2.04	1

Fig. S7 GO term analysis of 212 genes that are higher expressed in *cepd*. A) Gene Ontology (GO) term analysis (biological processes) of 212 differentially expressed genes (log_2 FC < -1, *p* adj. < 0.05) in roots of 9-day-old *cepd* seedlings compared to Col-0 after two days of cultivation on LN (low nitrogen) medium. Bars represent the percentage of genes found per GO term in the group of 212 genes (dark grey) and the percentage of genes representing the respective GO term found within the Arabidopsis genome (light grey). GO terms with > 5-fold enrichment against the genome are shown. Statistical analysis was performed using Fisher's Exact test and False Discovery Rate (FDR) < 0.05. **B)** List of the ten most highly differentially expressed genes. The number of TGA binding sites (TGACG and TACGTA) was counted in the region 2 kb upstream of the transcriptional start site. TPM: transcripts per million.

Fig. S7 supports Fig. 4.

Supplementary Fig. S8

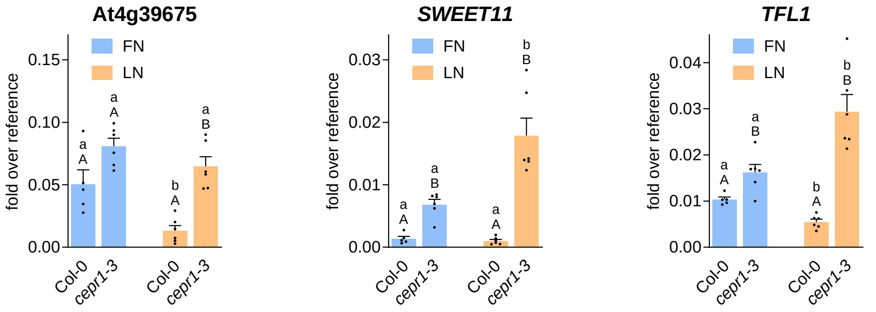

Fig. S8 Transcript levels of CEPD-repressed genes in roots of Col-0 and *cepr1-3* grown under full and limiting N supply. 7-day-old seedlings grown on full nitrogen (FN) medium were transferred to either FN or low nitrogen (LN) plates. Two days later, roots were collected for RNA isolation. Expression of the indicated genes was analyzed by RT-qPCR, *UBQ5* was used as a reference gene. Mean values of four to five biological replicates are shown with one replicate originating from one plate with 10 seedlings. Error bars represent the standard error of the mean. Lowercase letters indicate statistically significant differences within the genotype between the treatments, uppercase letters indicate significant differences within treatment between the genotypes. Statistical analyses were performed with logarithmic values by using two-way ANOVA and Fisher's Least Significant Difference test ($p < 0.05$).

Fig. S8 supports Fig. 4.

Supplementary Fig. S9

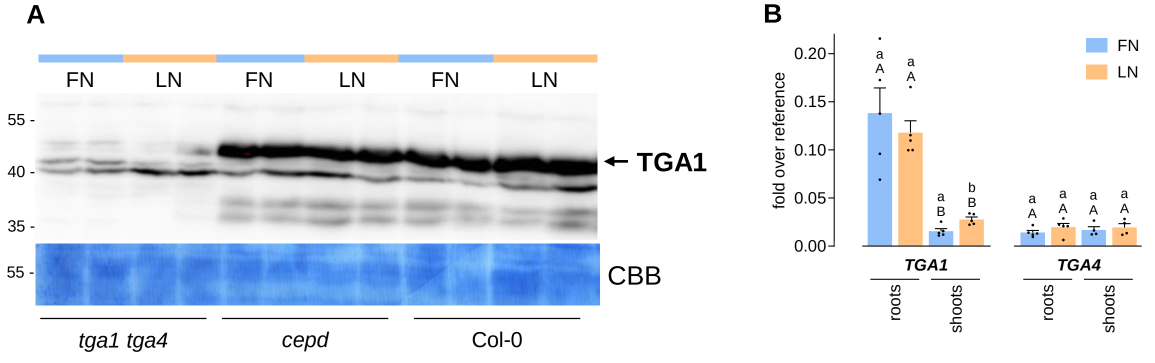

Fig. S9 TGA1 protein and *TGA1* and *TGA4* transcript levels in plants grown under full and limiting N supply. 7-day-old seedlings grown on full nitrogen (FN) medium were transferred to either FN or low nitrogen (LN) plates. Two days later, shoots and roots were collected for protein or RNA extraction. **A)** Western blot analysis of two independent protein extracts obtained from roots of Col-0, *cepd* and *tga1 tga4* plants, respectively. A commercially available antibody against a peptide of TGA1 was used. Molecular mass of marker proteins loaded on the gel are indicated in kDa. The PVDF membrane stained with Coomassie Brilliant Blue (CBB) was used as a loading control. **B)** Expression of *TGA1* and *TGA4* in Col-0 was analyzed by RT-qPCR, *UBQ5* was used as a reference gene. Mean values of four to five biological replicates are shown with one replicate originating from one plate with 10 seedlings. Error bars represent the standard error of the mean. Lowercase letters indicate statistically significant differences within the tissue between the conditions, uppercase letters indicate significant differences within treatment between the tissues. Statistical analyses were performed with logarithmic values by using two-way ANOVA and Fisher's Least Significant Difference test (p-value < 0.05).

Fig. S9 supports Fig. 6.

Supplementary Fig. S10

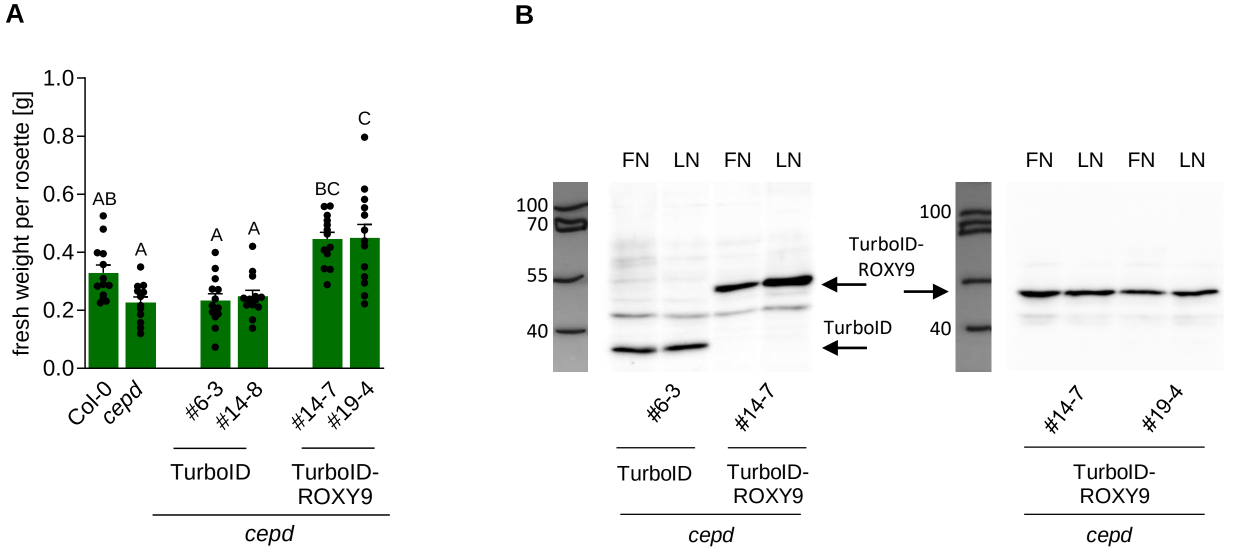

Fig. S10 Characterization of TurboID-ROXY9 lines. A) Fresh weight of the rosettes of *cepd* plants ectopically expressing either TurboID or TurboID-ROXY9. Plants were grown for 4 weeks in 12/12 h light regime. Mean values of 12 biological replicates per line are shown. Error bars represent the standard error of the mean. Letters indicate statistically significant differences between the genotypes. Statistical analysis was performed by one-way ANOVA and Tukey's multiple comparisons test (p adj. < 0.05). **B)** Western blot analysis of urea extracts from TurboID- or TurboID-ROXY9-expressing plant lines using an antibody against the HA tag. Extracts are from roots of axenically grown plants cultivated under the FN/FN and FN/LN regimes. FN, full nitrogen; LN, low nitrogen. Molecular masses of marker proteins are in kDa.

Fig. S10 supports Fig. 7.

Supplementary Fig. S11

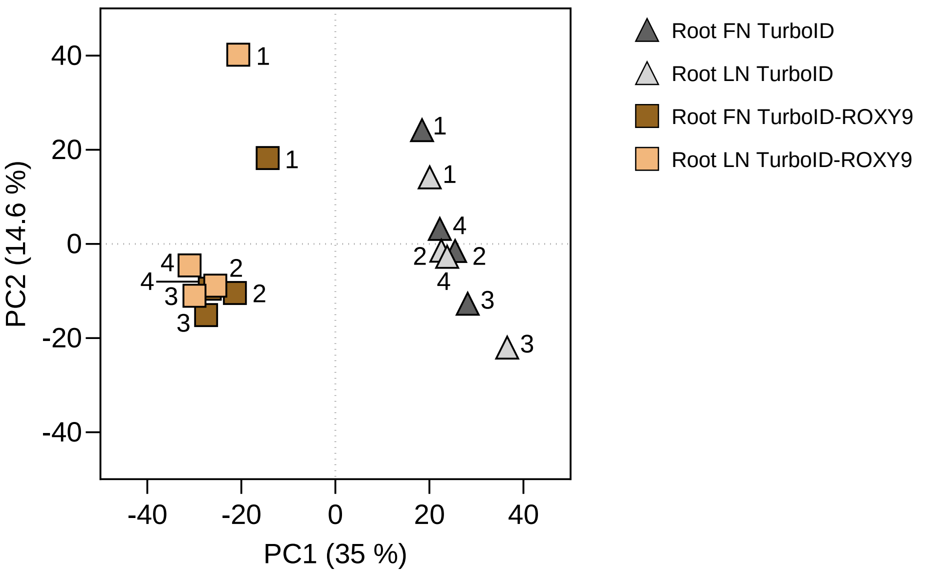

Fig. S11 Principal component analysis of proteins in TurboID-ROXY9 samples compared to TurboID controls. Symbols represent four independent experiments performed with plants grown under the FN/FN and FN/LN regimes. FN, full nitrogen; LN low nitrogen.
Fig. S11 supports Fig. 7.

Supplementary Fig. S12

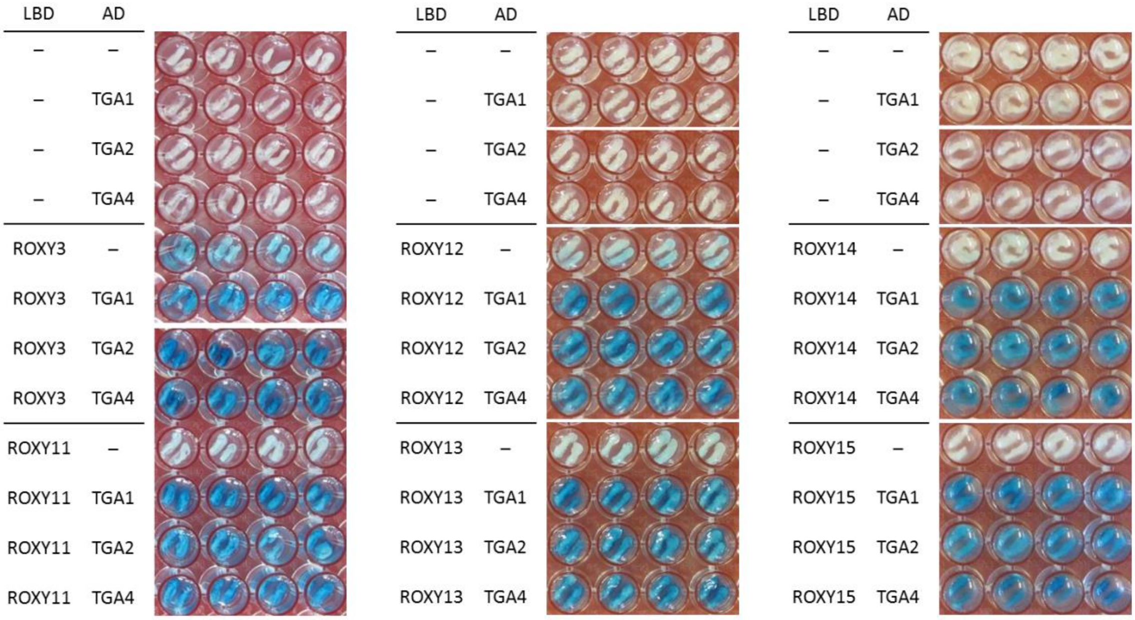

Fig. S12 Yeast two hybrid analysis with TGAs and ROXYs. *Saccharomyces cerevisiae* EGY48 (p8op-lacZ) strains expressing different combinations of hybrid proteins composed of the LexA DNA binding domain (LBD) fused to ROXYs and the B42 activation domain (AD) fused to *Arabidopsis thaliana* TGAs were streaked on agar medium placed in wells of a microtiter plate. Pictures were taken one day later. Blue colony color indicates positive interaction due to activation of the *Escherichia coli* ß-galactosidase (*lacZ*) reporter gene, whereas white colony color indicates no interaction. Eight independent yeast transformants (two per well) were analyzed.

Fig. S12 supports Fig. 8.

Supplementary Fig. S13

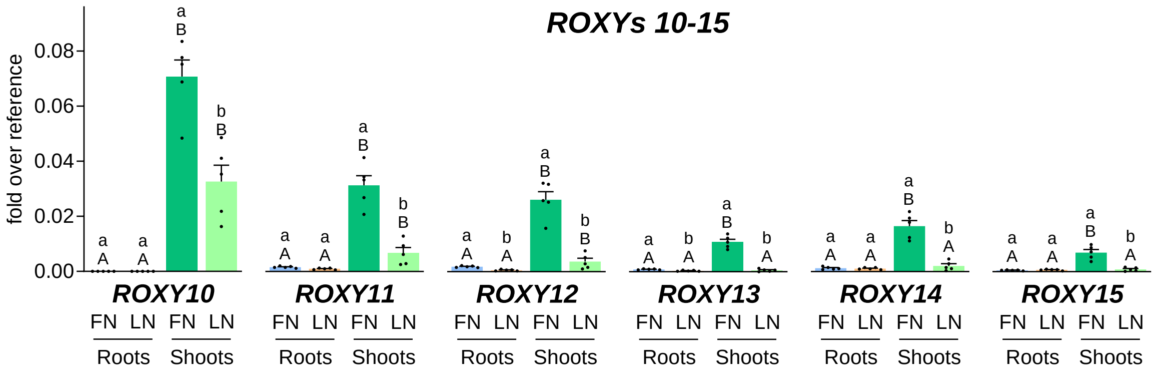

Fig. S13 Transcript levels of *ROXYs 10-15* in roots and shoots of Col-0 grown under full and limiting N supply. 7-day-old seedlings grown on full nitrogen (FN) medium were transferred to either FN or low nitrogen (LN) plates. Two days later, shoots and roots were collected for RNA isolation. Expression of the indicated genes was analyzed by RT-qPCR, *UBQ5* was used as a reference gene. Mean values of four to five biological replicates are shown, with one replicate being represented by material from one plate with 10 seedlings. Error bars represent the standard error of the mean. Lowercase letters indicate statistically significant differences within the tissue between the conditions, uppercase letters indicate significant differences within treatment between the tissues. Statistical analyses were performed with the logarithmic values by using two-way ANOVA and Fisher's Least Significant Difference test (p-value < 0.05).

Fig. S13 supports Fig. 8.

Supplementary Fig. S14

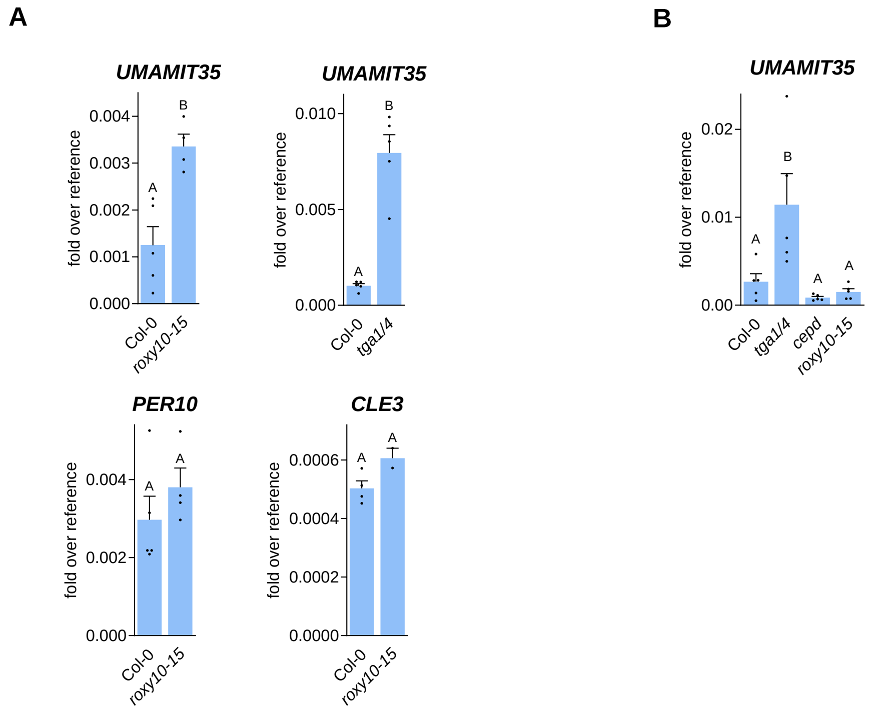

Fig. S14 ***UMAMIT35, PER10,* and *CLE3* transcript levels of in roots of Col-0 and *roxy10-15* plants grown under full nitrogen supply. A)** 7-day-old seedlings grown on full nitrogen (FN) medium were transferred to FN plates. Two days later, roots were collected for RNA isolation. **B)** Seedlings were grown for 12 days on FN. Expression of the indicated genes was analyzed by RT-qPCR, *UBQ5* was used as a reference gene. **A)** and **B)** Mean values of five biological replicates are shown with one replicate originating from one plate with 10 seedlings. Error bars represent the standard error of the mean. Letters indicate significant differences between the genotypes. Statistical analyses were performed with logarithmic values by unpaired t-test in **A)** (p-value > 0.05) and by one-way ANOVA and Tukey's multiple comparisons test in **B)** (p adj. < 0.05). Data from Col-0 and the *tga1 tga4* mutant in **A)** are the same as in Fig. 2B.
Fig. S14 supports Fig. 8.

Supplementary Fig. S15

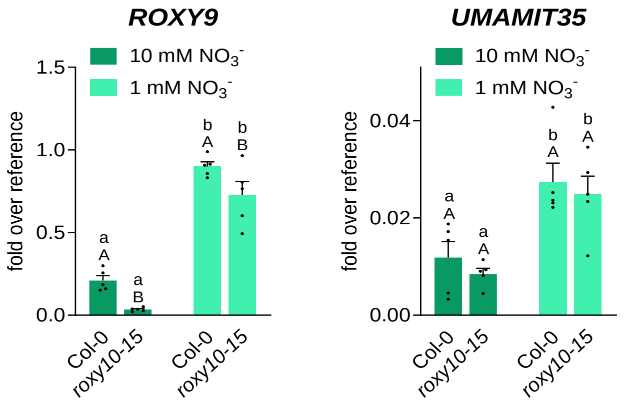

Fig. S15 Transcript levels of *ROXY9* and *UMAMIT35* in shoots and roots of plants cultivated under sufficient or limiting N conditions. RNA was isolated from roots and shoots of 9-day-old seedlings grown on 10 mM NO_3^- or 1 mM NO_3^-. Expression of the indicated genes was analyzed by RT-qPCR (shoots: *ROXY9*; roots: *UMAMIT35*), *UBQ5* was used as a reference gene. Mean values of five biological replicates are shown with one replicate originating from one plate with 10 seedlings. Error bars represent the standard error of the mean. Lowercase letters indicate statistically significant differences within the genotype between the treatments, uppercase letters indicate significant differences within treatment between the genotypes. Statistical analysis was performed with logarithmic values by two-way ANOVA and Fisher's Least Significant Difference test ($p < 0.05$).
Fig. S15 supports Fig. 8.

Supplementary Fig. S16

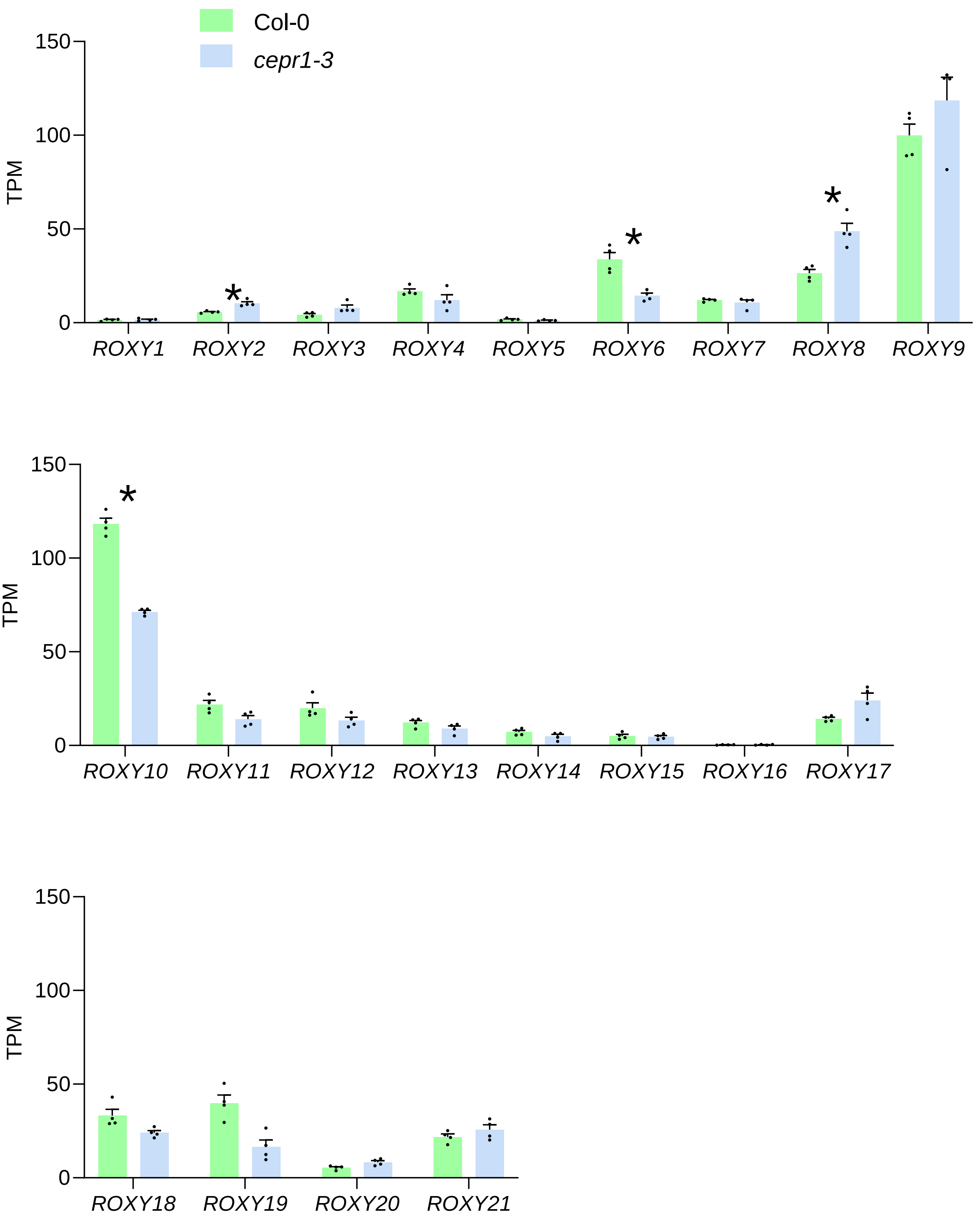

Fig. S16 Expression of *ROXYs 1-21* in shoots of Col-0 and *cepr1-3* seedlings after transfer from FN to LN medium. 7-day-old seedlings grown on full nitrogen (FN) medium were transferred to low nitrogen (LN) plates. Two days later, shoots were collected for RNA isolation. Expression of the indicated genes was analyzed by RNA-seq. TPM: transcript per million. Mean values of four biological replicates are shown with one replicate originating from one plate with 10 seedlings. Error bars represent the standard error of the mean. Statistical analyses were performed by using multiple unpaired t-tests. The star denotes significant differences between Col-0 and *cepr1-3* (p-value < 0.05).
Fig. S16 supports Fig. 8.

Supplementary Fig. S17

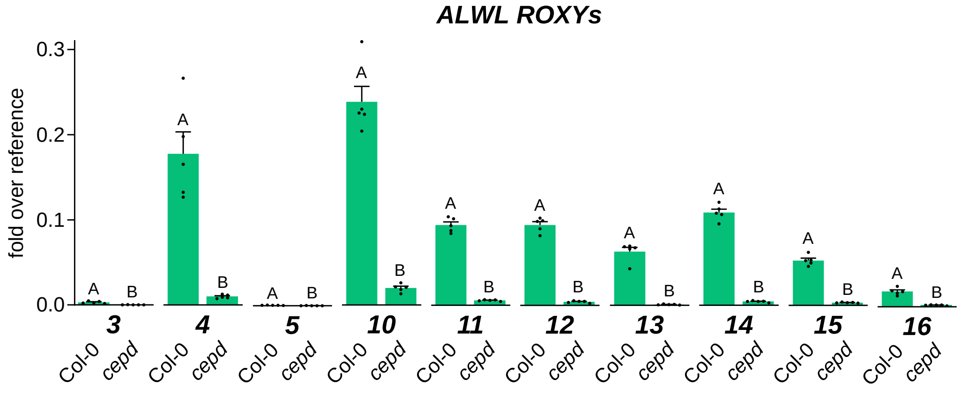

Fig. S17 *B-γ ALWL ROXY* expression in *cepd.* RNA from Col-0 and *cepd* shoot material belonging to the experiment shown in Fig. 8 was used. Plants were grown on 10 mM NO_3^-. Mean values of five biological replicates are shown with one replicate originating from one plate with 10 seedlings. Error bars represent the standard error of the mean. Letters indicate statistically significant differences between the genotypes. Statistical analyses were performed with the logarithmic values by using an unpaired t-test (*p*-value < 0.05).

Fig. S17 supports Fig. 8.

.

Supplementary Fig. S18

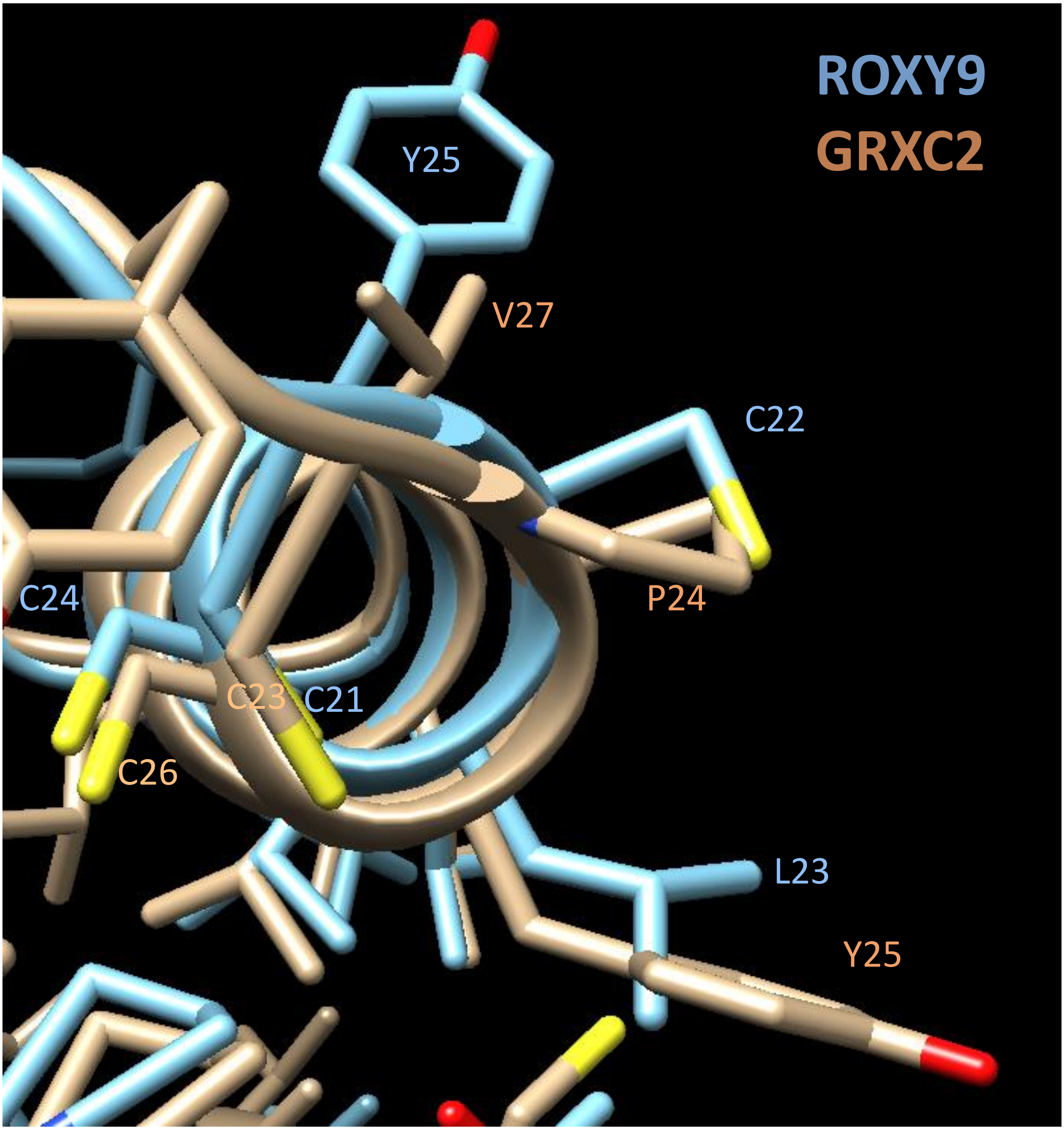

Fig. S18 Structural analysis of ROXY9 and GRXC2. Super-positioning of the structures of alpha helix 2 with the active site motifs of ROXY9 ($C_{21}C_{22}L_{23}C_{24}Y_{25}$ cyan blue) and GRXC2 ($C_{23}P_{24}Y_{25}C_{26}V_{27}$ brown). The backbones of the structures are found on https://alphafold.ebi.ac.uk/entry/O82255 (ROXY9) and https://alphafold.ebi.ac.uk/entry/B3H604 (GRXC2).
Fig. S18 supports Fig. 9A.

Supplementary Fig. S19

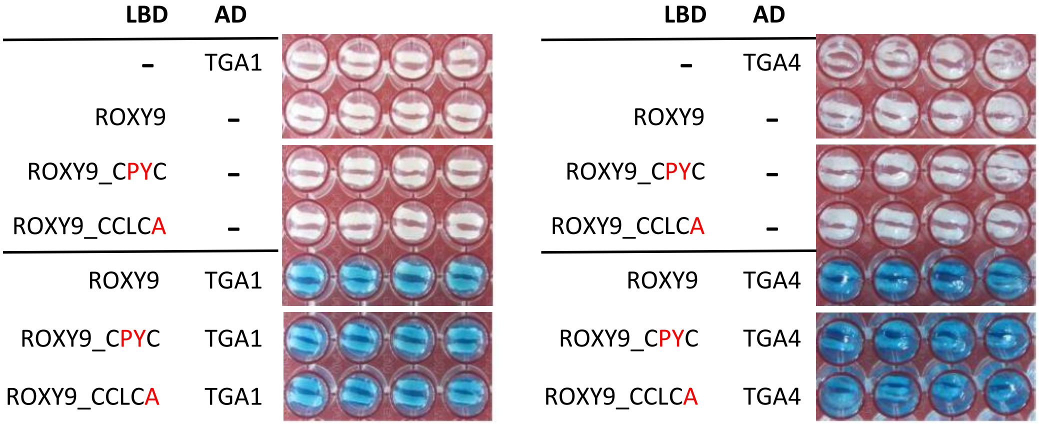

Fig. S19 Yeast two hybrid analysis with TGA1/4 and ROXY9 active site mutants. *Saccharomyces cerevisiae* EGY48 (p8op-lacZ) strains expressing different combinations of hybrid proteins composed of the LexA DNA binding domain (LBD) fused to ROXYs and the B42 activation domain (AD) fused to *Arabidopsis thaliana* TGA1 or TGA4 were streaked on agar medium placed in wells of a microtiter plate. Pictures were taken one day later. Blue colony color indicates positive interaction due to activation of the *Escherichia coli* ß-galactosidase (*lacZ*) reporter gene, whereas white colony color indicates no interaction. Eight independent yeast transformants (two per well) were analyzed.
Fig. S19 supports Fig. 9A.

Supplementary Fig. S20

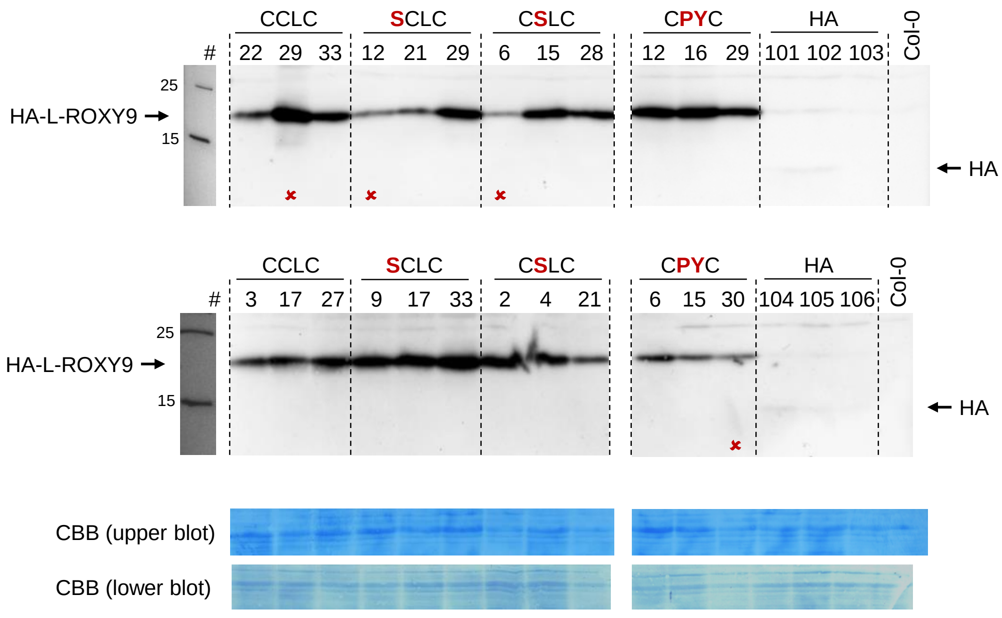

Fig. S20 Western blot analysis of transgenic *cepd* plants expressing *ROXY9* or *ROXY9* active site mutants under the control of the *CaMV 35S* promoter. Seedlings of six independent lines (T2 generation) per construct grown for seven days on full nitrogen (FN) plates were transferred to low nitrogen (LN) plates. Two days later, proteins were extracted from roots. The αHA antibody was used as a primary antibody. Lanes marked with the red cross were excluded for further analysis as they had too high or too low HA-L-ROXY9 levels. PVDF membranes stained with Coomassie Brilliant Blue (CBB) were used as a loading control. Numbers on the left denote molecular masses (kDa) of marker proteins loaded on the gel. The L in HA-L-ROXY9 stands for the peptide linker inserted between the tag and the coding region.

Fig. S20 supports Fig. 9A.

Supplementary Fig. S21

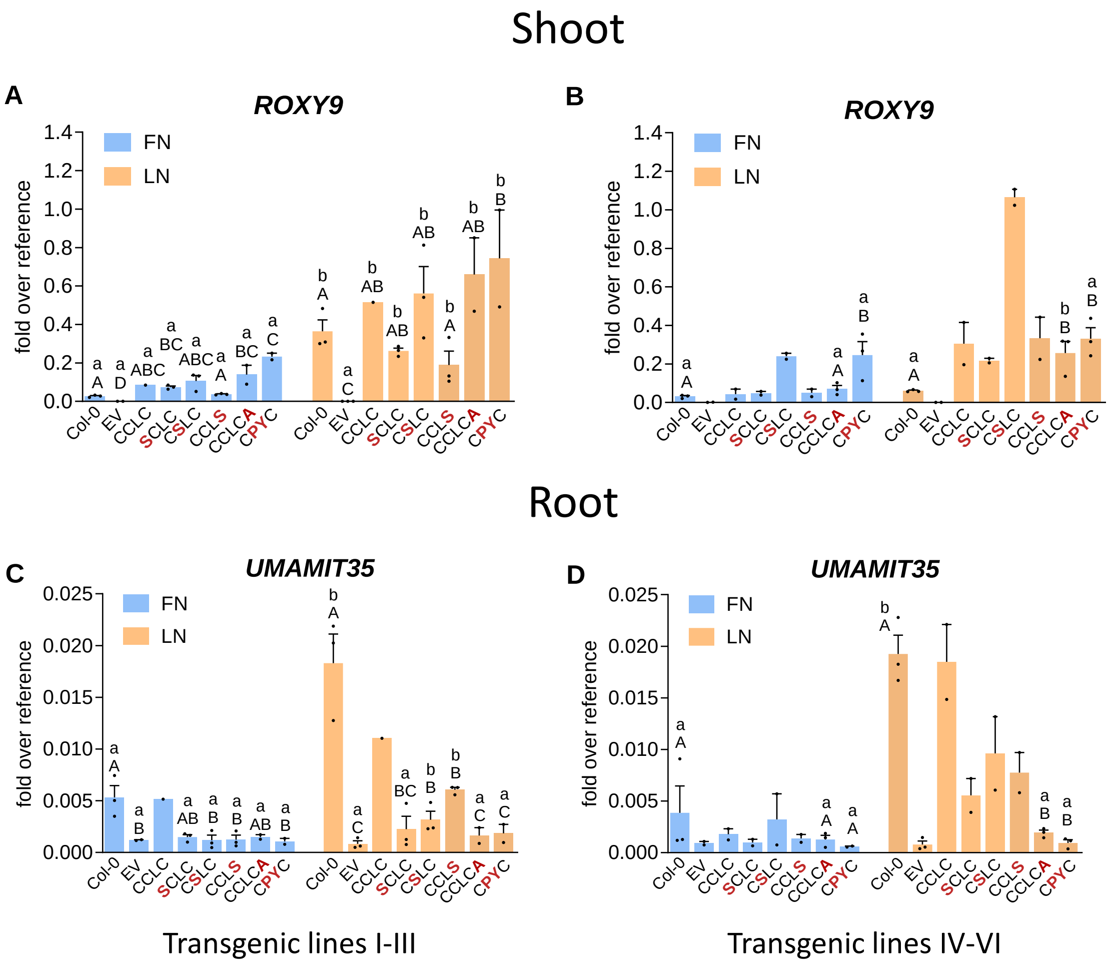

Fig. S21 Analysis of transgenic *cepd* plants expressing *ROXY9* or *ROXY9* active site variants under the control of the *ROXY9* promoter. Seedlings of three to six independent lines (T2 generation) per construct were first cultivated for seven days on full nitrogen (FN) plates and were subsequently transferred either to FN or to low nitrogen (LN) plates. Two days later, shoots and roots were collected for RNA isolation. Gene expression was analyzed by RT-qPCR, *UBQ5* was used as a reference gene. **A)** and **C)** Results from three independent transgenic lines expressing similar levels of *ROXY9* active site variants as Col-0. Since only one transgenic line expressing wild-type-like levels of ROXY9_CCLC was obtained, it was excluded from statistical analysis. **B)** and **D)** Results from two or three independent transgenic lines expressing approximately 4-fold higher levels of *ROXY9* active site variants as Col-0 under LN conditions, which might be similar to transcript levels of all four *CEPDs* in Col-0. Data with only two values were excluded from statistical analysis. **A)** to **D)** One replicate originates from one plate with 10 seedlings each. Error bars represent the standard error of the mean. Lowercase letters indicate statistically significant differences within the genotype between the treatments, uppercase letters indicate significant differences within treatment between the genotypes. Statistical analyses were performed with logarithmic values by using two-way ANOVA and Bonferroni's multiple comparisons test (p adj. < 0.05). EV, empty vector control. **A)** and **B)** Note that the primer does not recognize the mutated *ROXY9* transcript in the *cepd* background.
Fig. S21 supports Fig. 9A.

Supplementary Fig. S22

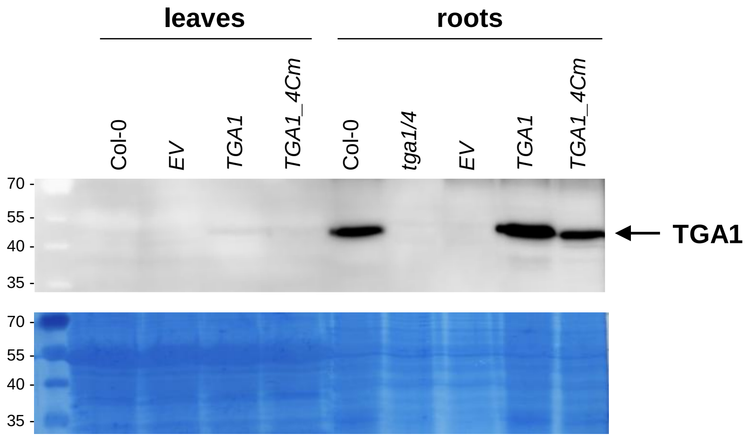

Fig. S22 Characterization of TGA1 complementation lines. Western blot analysis of protein extracts obtained from leaves and roots of soil-grown wild-type *Arabidopsis thaliana* (Col-0) and *tga1 tga4* plants complemented either with the empty vector (EV), a wild-type *TGA1* genomic construct (*TGA1*) or a mutated *TGA1* genomic construct carrying mutations in four cysteine residues (*TGA1_4Cm*). TGA1 protein levels were detected using an αTGA1 antibody. Coomassie blue staining served as a loading control. Numbers on the left denote molecular masses (kDa) of marker proteins loaded on the gel.

Fig. S22 supports Fig. 9B.

Supplementary Fig. S23

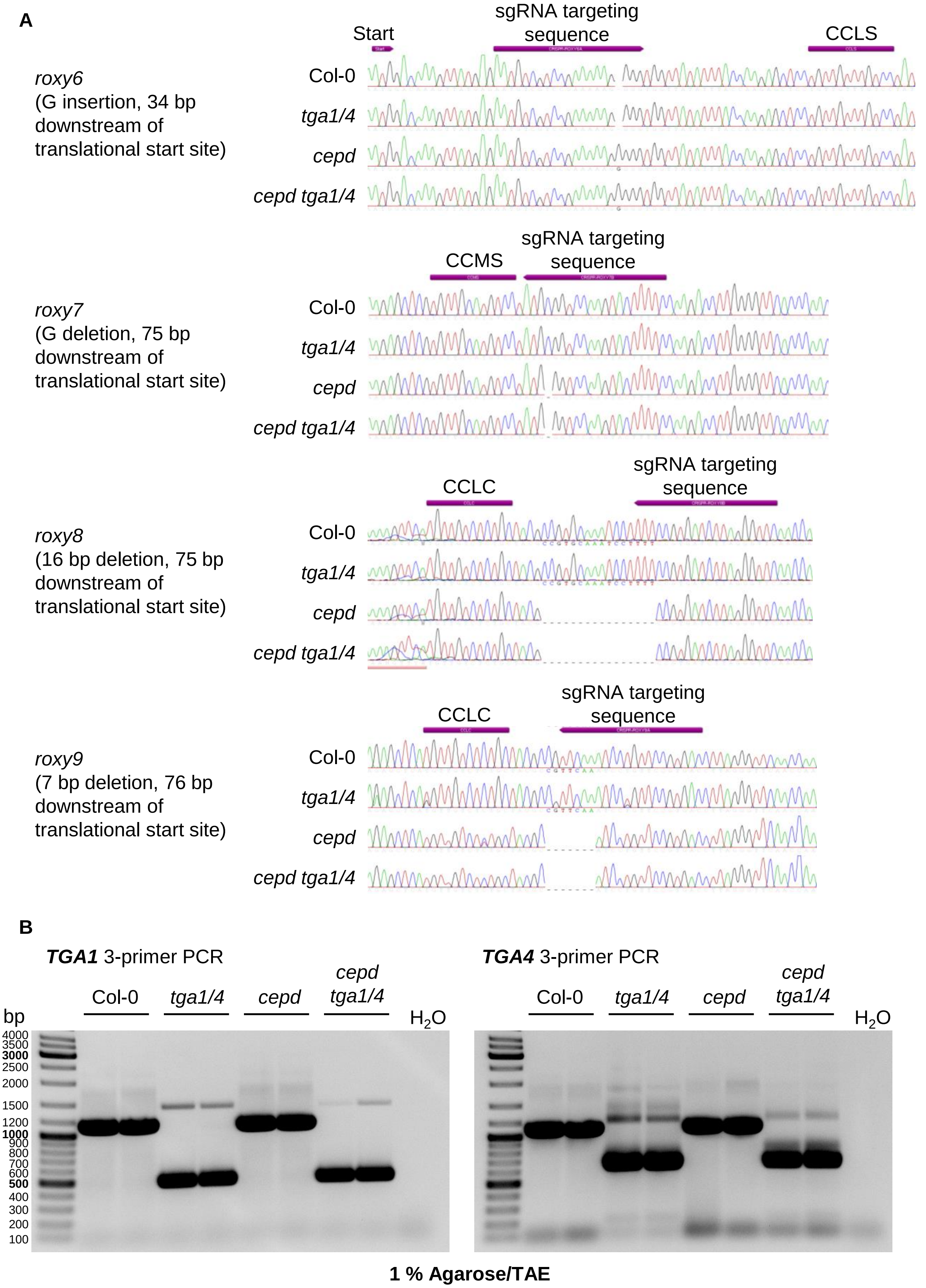

Fig. S23 Confirmation of the six mutant alleles in *cepd tga1 tga4*. Specific PCR products were amplified with primers listed in Supplementary Table 1. **A)** PCR products obtained for *ROXY6*, *7*, *8* or *9* alleles were subjected to Sanger sequencing to verify the homozygous presence of wild-type DNA or mutations, respectively. Colour code of the DNA sequence: red, T; green, A; black, G; blue, C. **B)** For *TGA1* and *TGA4*, homozygous wild-type or T-DNA insertion alleles were confirmed by 3-primer PCR. For each genotype, two independent DNA preparations were analyzed.
Fig. S23 supports Materials and Methods.

Supplementary Fig. S24

Plasmid backbone: pB2GW7 (Karimi *et al*., 2002)

CaMV 35S promoter
Fusion protein start codon
HA tag
attB1, attB2
linker
ROXY9
CaMV 35S terminator

Sequence:

ACTAGAGCCAAGCTGATCTCCTTTGCCCCGGAGATCACCATGGACGACTTTCTCTATCTCTACGATCTAGGAA
GAAAGTTCGACGGAGAAGGTGACGATACCATGTTCACCACCGATAATGAGAAGATTAGCCTCTTCAATTTCAG
AAAGAATGCTGACCCACAGATGGTTAGAGAGGCCTACGCGGCAGGTCTCATCAAGACGATCTACCCGAGTAAT
AATCTCCAGGAGATCAAATACCTTCCCAAGAAGGTTAAAGATGCAGTCAAAAGATTCAGGACTAACTGCATCA
AGAACACAGAGAAAGATATATTTCTCAAGATCAGAAGTACTATTCCAGTATGGACGATTCAAGGCTTGCTTCA
TAAACCAAGGCAAGTAATAGAGATTGGAGTCTCTAAGAAAGTAGTTCCTACTGAATCAAAGGCCATGGAGTCA
AAAATTCAGATCGAGGATCTAACAGAACTCGCCGTGAAGACTGGCGAACAGTTCATACAGAGTCTTTTACGAC
TCAATGACAAGAAGAAAATCTTCGTCAACATGGTGGAGCACGACACTCTCGTCTACTCCAAGAATATCAAAGA
TACAGTCTCAGAAGACCAAAGGGCTATTGAGACTTTTCAACAAAGGGTAATATCGGGAAACCTCCTCGGATTC
CATTGCCCAGCTATCTGTCACTTCATCAAAAGGACAGTAGAAAAGGAAGGTGGCACCTACAAATGCCATCATT
GCGATAAAGGAAAGGCTATCGTTCAAGATGCCTCTGCCGACAGTGGTCCCAAAGATGGACCCCCACCCACGAG
GAGCATCGTGGAAAAAGAAGACGTTCCAACCACGTCTTCAAAGCAAGTGGATTGATGTGATATCTCCACTGAC
GTAAGGGATGACGCACAATCCCACTATCCTTCGCAAGACCCTTCCTCTATATAAGGAAGTTCATTTCATTTGG
AGAGGACTCCGGTATTTTTACAACAATTACCACAACAAAACAAACAACAAACAACATTACAATTTACTATTCT
AGTCGAGAACA**ATG**GCATACCCATACGACGTTCCGGACTACGCTTCTTTGGGTGGTTCTAGCCCAAGCTCAGA
GCTCCACCGCGGTGGCGGCCGCATCTTTTACCCATACGATGTTCCTGACTATGCGGGCTATCCCTATGACGTC
CCGGACTATGCAGATATCTCTAGGCAGATCACAAGTTTGTACAAAAAAGCAGGCTCCGGAGGAGGAGGTTCAG
GTGGTGGTGGATCCGGAGGAGGTGGTTCAAATGCAATGATGGACAAAGTGATGAGAATGTCTTCAGAGAAAGG
AGTGGTGATCTTCACGAAGAGCTCATGTTGTCTCTGCTACGCCGTTCAAATCCTGTTCCGTGACCTTAGGGTT
CAACCAACCATCCACGAGATCGACAACGACCCGGACTGCCGTGAGATCGAGAAGGCTCTTCTCCGGCTCGGCT
GTTCCACGGCGGTTCCAGCTGTCTTTGTCGGAGGCAAGCTTGTTGGCTCCACCAATGAAGTCATGTCCCTTCA
CCTTAGTGGCTCTCTTGTCCCATTGATCAAACCCTATCAGTCCATCCTTTACTAGCAAGACCCAGCTTTCTTG
TACAAAGTGGTGATATCCCGCGGCCATGCTAGAGTCCGCAAAAATCACCAGTCTCTCTCTACAAATCTATCTC
TCTCTATTTTTCTCCAGAATAATGTGTGAGTAGTTCCCAGATAAGGGAATTAGGGTTCTTATAGGGTTTCGCT
CATGTGTTGAGCATATAAGAAACCCTTAGTATGTATTTGTATTTGTAAAATACTTCTATCAATAAAATTTCTA
ATTCCTAAAACCAAAATCCAGTGACC

Encoded fusion protein:

MAYPYDVPDYASLGGSSPSSELHRGGGRIFYPYDVPDYAGYPYDVPDYADISRQITSLYKKAGSGGGGSGGGG
SGGGGSNAMDKVMRMSSEKGVVIFTKSSCCLCYAVQILFRDLRVQPTIHEIDNDPCREIEKALLRLGCSTAV
PAVFVGGKLVGSTNEVMSLHLSGSLVPLIKPYQSILY

Fig. S24 Sequence of the *CaMV 35S:HA$_3$-L-ROXY9* construct.
Fig. S24 supports Materials and Methods.

Supplementary Fig. S25

Plasmid backbone: pUBQ10GW7-HA (Uhrig *et al.*, 2017)

UBQ10 promoter with 5'-UTR and 1. intron
Fusion protein start codon
HA tag
TurboID
ROXY9
CaMV 35S terminator

Sequence:
GTGGTTGCAGCCGGCACACACGAGTCGTGTTTATCAACTCAAAGCACAAATACTTTTCCTCAACCTAAAAATA
AGGCAATTAGCCAAAAACAACTTTGCGTGTAAACAACGCTCAATACACGTGTCATTTTATTATTAGCTATTGC
TTCACCGCCTTAGCTTTCTCGTGACCTAGTCGTCCTCGTCTTTTCTTCTTCTTCTTCTATAAAACAATACCCA
AAGAGCTCTTCTTCTTCACAATTCAGATTTCAATTTCTCAAAATCTTAAAAACTTTCTCTCAATTCTCTCTAC
CGTGATCAAGGTAAATTTCTGTGTTCCTTATTCTCTCAAAATCTTCGATTTTGTTTTCGTTCGATCCCAATTT
CGTATATGTTCTTTGGTTTAGATTCTGTTAATCTTAGATCGAAGACGATTTTCTGGGTTTGATCGTTAGATAT
CATCTTAATTCTCGATTAGGGTTTCATAGATATCATCCGATTTGTTCAAATAATTTGAGTTTTGTCGAATAAT
TACTCTTCGATTTGTGATTTCTATCTAGATCTGGTGTTAGTTTCTAGTTTGTGCGATCGAATTTGTCGATTAA
TCTGAGTTTTTCTGATTAACAGCTCGAGAACA**ATG**TACCCATACGATGTTCCTGACTATGCGGGCTATCCCTA
TGACGTCCCAGACTACGCAGCTAGCAAAGACAATACTGTGCCTCTGAAGCTGATCGCTCTCCTGGCTAATGGC
GAGTTCCATAGTGGCGAACAGCTGGGAGAAACCCTGGGCATGTCCAGGGCCGCTATCAACAAGCACATTCAGA
CTCTGCGCGACTGGGGCGTGGACGTGTTCACCGTGCCCGGAAAGGGCTACTCTCTGCCCGAGCCTATCCCGCT
GCTGAACGCTAAACAGATTCTGGGACAGCTGGACGGCGGGAGCGTGGCAGTCCTGCCTGTGGTCGACTCCACC
AATCAGTACCTGCTGGATCGAATCGGCGAGCTGAAGAGTGGGGATGCTTGCATTGCAGAATATCAGCAGGCAG
GGAGAGGAAGCAGAGGGAGGAAATGGTTCTCTCCTTTTGGAGCTAACCTGTACCTGAGTATGTTTTGGCGCCT
GAAGCGGGGACCAGCAGCAATCGGCCTGGGCCCGGTCATCGGAATTGTCATGGCAGAAGCGCTGCGAAAGCTG
GGAGCAGACAAGGTGCGAGTCAAATGGCCCAATGACCTGTATCTGCAGGATAGAAAGCTGGCAGGCATCCTGG
TGGAGCTGGCCGGAATAACAGGCGATGCTGCACAGATCGTCATTGGCGCCGGGATTAACGTGGCTATGAGGCG
CGTGGAGGAAAGCGTGGTCAATCAGGGCTGGATCACACTGCAGGAAGCAGGGATTAACCTGGACAGGAATACT
CTGGCCGCTACGCTGATCCGAGAGCTGCGGGCAGCCCTGGAACTGTTCGAGCAGGAAGGCCTGGCTCCATATC
TGCCACGGTGGGAGAAGCTGGATAACTTCATCAATAGACCCGTGAAGCTGATCATTGGGGACAAAGAGATTTT
CGGGATTAGCCGGGGGATTGATAAACAGGGAGCCCTGCTGCTGGAACAGGACGGAGTTATCAAACCCTGGATG
GGCGGAGAAATCAGTCTGCGGTCTGCCGAAAAGGGTACCATGGACAAAGTGATGAGAATGTCTTCAGAGAAAG
GAGTGGTGATCTTCACGAAGAGCTCATGTTGTCTCTGCTACGCCGTTCAAATCCTGTTCCGTGACCTTAGGGT
TCAACCAACCATCCACGAGATCGACAACGACCCGGACTGCCGTGAGATCGAGAAGGCTCTTCTCCGGCTCGGC
TGTTCCACGGCGGTTCCAGCTGTCTTTGTCGGAGGCAAGCTTGTTGGCTCCACCAATGAAGTCATGTCCCTTC
ACCTTAGTGGCTCTCTTGTCCCATTGATCAAACCCTATCAGTCCATCCTTTACTAGCAAGACCCAGCTTTCTT
GTACAAAGTGGTGATATCCCGCGGCCATGCTAGAGTCCGCAAAAATCACCAGTCTCTCTCTACAAATCTATCT
CTCTCTATTTTTCTCCAGAATAATGTGTGAGTAGTTCCCAGATAAGGGAATTAGGGTTCTTATAGGGTTTCGC
TCATGTGTTGAGCATATAAGAAACCCTTAGTATGTATTTGTATTTGTAAAATACTTCTATCAATAAAATTTCT
AATTCCTAAAACCAAAATCCAGTGACC

Encoded fusion protein:

MYPYDVPDYAGYPYDVPDYAASKDNTVPLKLIALLANGEFHSGEQLGETLGMSRAAINKHIQTLRDWGVDVFT
VPGKGYSLPEPIPLLNAKQILGQLDGGSVAVLPVVDSTNQYLLDRIGELKSGDACIAEYQQAGRGSRGRKWFS
PFGANLYLSMFWRLKRGPAAIGLGPVIGIVMAEALRKLGADKVRVKWPNDLYLQDRKLAGILVELAGITGDAA
QIVIGAGINVAMRRVEESVVNQGWITLQEAGINLDRNTLAATLIRELRAALELFEQEGLAPYLPRWEKLDNFI
NRPVKLIIGDKEIFGISRGIDKQGALLLEQDGVIKPWMGGEISLRSAEKGTMDKVMRMSSEKGVVIFTKSSCC
LCYAVQILFRDLRVQPTIHEIDNDPDCREIEKALLRLGCSTAVPAVFVGGKLVGSTNEVMSLHLSGSLVPLIK
PYQSILY

Fig. S25 Sequence of the *UBQ10:HA$_2$-TurboID-ROXY9* construct.
Fig. S25 supports Materials and Methods.

Supplementary Fig. S26

roxy6 (*cepd1*)

Start **G insertion** (C C L S)
ATG GAC AAA GTT ATG AGA ATG TCG TCC GAA AAA G**G**G GGT GGT TAT ATT TAC CAA GAG CTC CTG TTG TTT GTC CTA TGC GGT TCA A
PAM

roxy7 (*cepdl1*)

Start C C M S **1 bp deletion**
ATG GAC AAA GTT GTG AGA ATG TCG TCA GAG AAA GGA GTG GTT ATT TTC AGC AAG AGC TCG TGT TGC ATG TCC TAT G-GG TCC AAG TAC TTT TC
PAM

roxy8 (*cepdl2*)

Start C C L C **16 bp deletion**
ATG GAC AAG GTT ATG AGA ATG TCA TCG GAG AAA GGA GTC GTG ATC TTC ACC AAA AGT TCA TGT TGT CTC TGC TAC G----------------CC GTG
ATC TTA GGG TTC
PAM

roxy9 (*cepd2*)

Start C C L C **7 bp deletion**
ATG GAC AAA GTG ATG AGA ATG TCT TCA GAG AAA GGA GTG GTG ATC TTC ACG AAG AGC TCA TGT TGT CTC TGC TAC GC-------A TCC TGT TCC
GTG AC
PAM

roxy10

Start (C C M S) **78 bp deletion and A insertion**
ATG GAT AT GAT AAC GAA GAT GGT GAT GGA GAG AC---**A**
CAG CGG TTT ACG
PAM
PAM

roxy11-15

Start *ROXY15* **11617 bp deletion and TCTTT insertion** *ROXY11* (C C M S)
ATG GAG AAG ATA CAA AAG ATG ATC TCC GAG A--------//--------**TC TTT** AGT CGG *TAG TGA* TCT TTA GCA AGA ACT CTT GCT GCA TGT CTC ACA
PAM PAM *StopStop*

Fig. S26 CRISPR/Cas9-based genome editing of *ROXY* genes. The active site-encoding sequence is highlighted in yellow. Mutations leading to frameshifts and deletions are indicated in red. PAM, protospacer adjacent motif.
Fig. S26 supports Materials and Methods.

Supplementary Fig. S27

A

target 1-sgRNA scaffold-U6-26 terminator-U6-29 promoter-target 2

GNNNNNNNNNNNNNNNNNNNNGTTTTAGAGCTAGAAATAGCAAGTTAAAATAAGGCTAGTCCGTTATCAACT
TGAAAAAGTGGCACCGAGTCGGTGCTTTTTTTTGCAAAATTTTCCAGATCGATTTCTTCTTCCTCTGTTCTTC
GGCGTTCAATTTCTGGGGTTTTCTCTTCGTTTTCTGTAACTGAAACCTAAAATTTGACCTAAAAAAAATCTCAA
ATAATATGATTCAGTGGTTTTGTACTTTTCAGTTAGTTGAGTTTTGCAGTTCCGATGAGATAAACCAATATTAA
TCCAAACTACTGCAGCCTGACAGACAAATGAGGATGCAAACAATTTTAAAGTTTATCTAACGCTAGCTGTTTT
GTTTCTTCTCTCTGGTGCACCAACGACGGCGTTTTCTCAATCATAAAGAGGCTTGTTTTACTTAAGGCCAATA
ATGTTGATGGATCGAAAGAAGAGGGCTTTTAATAAACGAGCCCGTTTAAGCTGTAAACGATGTCAAAAACAT
CCCACATCGTTCAGTTGAAAATAGAAGCTCTGTTTATATATTGGTAGAGTCGACTAAGAGATTGNNNNNNNN
NNNNNNNNNNNN

Transcription initiation by RNA polymerase III

B

Primers:

ROXY10-sgRNA-BpiI-fwd
GAGAGAGAAGACATGATTGCGATTTCGGAGCAAATCCAGGTTTTAGAGCTAGAAATAGCAAG

29p-ROXY10-BpiI-rev
GAGAGAGAAGACATAAACCCGGTCTCTCCATCACCATCAATCTCTTAGTCGACTCTACC

ROXY11-15-sgRNA-BsaI-fwd
ATATATGGTCTCTGATTGAAAGATGATCTCCGAGAAGTGTTTTAGAGCTAGAAATAGCAAG

29p-ROXY11-BsaI-rev
ATTATTGGTCTCTAAACATCAAGTCATGAGTCTCCATCAATCTCTTAGTCGACTCTACC

Fig. S27 PCR products used for CRISPR/Cas9-based genome editing of the *ROXY10* and *ROXY11-15* loci. A) Scheme and sequence of the PCR product generated to clone two sgRNA expression cassettes (Xing *et al*., 2014) into the pBCsGFPEE vector (Nair *et al*., 2021). **B)** Primers used to introduce the *roxy10* and *roxy11-15* mutations shown in Supplementary Fig. 26, respectively. Fig. S27 supports Materials and Methods.

Supplementary Table S1: Primers for genotyping

	Primer ID	Sequence 5'-3'
roxy6 T7E1 assay	ROXY6 fwd	TTTCTTGTTGCATAGTTTGGGTCAC
	ROXY6 rev	TAAATATGGCTTCACTAGGGGAACG
roxy7 T7E1 assay	ROXY7fwd	ACCCTCTTTTCTTCAAACAGGAACC
	ROXY7 rev	AGACAAGAAGACAAATCGTTGCCTG
roxy8 T7E1 assay	ROXY8 downstream fwd	GCCGCTTTAATTCTTCGGAGGGAATCTG
	ROXY8 downstream rev	CATCAGTACATCCACCGATTAGTTAAGCTGG
roxy9 3 Primer PCR	ROXY9 genom-71 fwd	GCAAGAAAAAAACACACTCGAAAGACTC
	ROXY9 genom568 rev	GTTGATTAACTAAACTGAAACACGAGAGAAGC
	roxy9A28 fwd	GCTCATGTTGTCTCTGCTACGCATC
roxy10 T7E1 assay	ROXY10genom-130fwd	TGAACTTGTTGTCGCTTTCGGAC
	ROXY10genom609rev	ACACACTGATTATTGATCCGACGTG
roxy11-15 3 Primer PCR	ROXY11genom 755 rev	TGCACGTGTATTCATTTCTAGATGC
	ROXY15genom-135 fwd	CATCCAACGCATAATGTCATAGC
	ROXY15genom 503 rev	CATCCTTGATTGGTTTCATGACATC
tga1 3 Primer PCR	tga1 SALK_028210 RP	CGTGTCCCCTCTGGTTTCTTTC
	tga1 SALK_028210 LP	AACCTGGATTCATGGTTTCCG
	LBb1	GCGTGGACCGCTTGCTGCAACT
tga4 3 Primer PCR	tga4 SALK_127923 RP	GACACATTTTGTTCCACCGAG
	tga4 SALK_127923 LP	GGTCTAAATCCGCCTATCCAC
	LBb1	GCGTGGACCGCTTGCTGCAACT

T7E1 assay: (Sentmanat et al., 2018)

Sentmanat, M.F., Peters, S.T., Florian, C.P., Connelly, J.P., and Pruett-Miller, S.M. (2018). A Survey of Validation Strategies for CRISPR-Cas9 Editing. Sci Rep-Uk **8**.

Supplementary Table S2: Primers for cloning

Primer	Sequence 5'-3'
ROXY9 promoter fwd (-1135)	GGGGACAAGTTTGTACAAAAAAGCAGGCTCATCTGGATTTAGCATAATATCTTCCG
ROXY9 promoter rev (+2340)	GGGGACCACTTTGTACAAGAAAGCTGGGTGATGGGGAAAAAGAGACAGGATGG
ROXY9 CPYC fwd	GAAGAGCTCATGTCCATATTGCTACGCCGTTCAAATCCTGTTCCG
ROXY9 CPYC rev	GGCGTAGCAATATGGACATGAGCTCTTCGTGAAGATCACCACTCC
ROXY9 CCLCA fwd	GTTGTCTCTGCGCCGCCGTTCAAATCCTGTTCCGTGAC
ROXY9 CCLCA rev	GATTTGAACGGCGGCGCAGAGACAACATGAGCTCTTCGTGAAG

Supplementary Table S3: Single guide RNA (sgRNA) targeting sequences

	Sequence 5'-3'
ROXY6	AATGTCGTCCGAAAAAGGGG
ROXY7	GAAAAGTACTTGGACCGCAT
ROXY8	GAACCCTAAGATCACGGAAA
ROXY9	GTCACGGAACAGGATTTGAA
ROXY10	GATGGTGATGGAGAGACCGG
	CGATTTCGGAGCAAATCCAG
ROXY11-15	AAAGATGATCTCCGAGAAGT
	ATGGAGACTCATGACTTGAT

Supplementary Table S4: Primers for RT-qPCR

Primer	Sequence 5'-3'	Reference
At4G39675	Quantitect QT00729456	Qiagen, Netherlands
CEPH	TTGGGCTCTGTCTCTTCTTTA<br>AACGCTCCTTCTTCTGTTCC	
CLE3	CCTGCTTCTAGTACTCGAATTGAC<br>ATTTCCAAGGATCGTCTCTTCGCC	Araya et al., 2014
NRT2.2	GCAGCAGATTGGCATGCATTT<br>AAGCATTGTTGGTTGCGTTCC	Ruffel *et al.,* 2021
NRT2.4	GAACAAGGGCTGACATGGAT<br>GCTTCTCGGTCTCTGTCCAC	Ruffel *et al.,* 2021
PER10	CTCTCTAAGCTAAAGGACACGTG<br>GTTCGCGTAGTAAGCATTGTCAAAC	
PER71	AGCATTAGCCGCTCGTGACACAG<br>GGACGACGAGATCACGAGTATTGAGT	Shigeto *et al.,* 2015
ROXY3	QuantiTect QT01713607	Qiagen, Netherlands
ROXY4	QuantiTect QT00797622	Qiagen, Netherlands
ROXY5	QuantiTect QT00725788	Qiagen, Netherlands
ROXY6	Quantitect QT00852516	Qiagen, Netherlands
ROXY7	Quantitect QT00760144	Qiagen, Netherlands
ROXY8	Quantitect QT00797629	Qiagen, Netherlands
ROXY9	TTCACCTTAGTGGCTCTCTTGTC<br>CTAGCTCACACTCTAGCTGTAACTGTG	
ROXY9 endogenous in Col-0 and all variants of transgenes	CGCCGTTCAAATCCTGTTC<br>GGACATGACTTCATTGGTGGAG	comment: yellow sequence missing in *ROXY9* transcript in *cepd*
ROXY10	AGCCAACGAGGTCATGAGTCTAC<br>AGCCCGCTTAAGCATGGGAATC	
ROXY11	GCGTGAACCCGACGATCTATGAAC<br>CCTATGAACACCACTGGCACTGTC	
ROXY12	ACTTTGGCGTGAACCCGACTATC<br>CCAATGCTTGCTCTATCTCCCTTC	
ROXY13	TCCATCTCAATCGCTCTCTGGTTC<br>ATCAAAGCCATAGTGCTCCAACCC	
ROXY14	TTCATAGGAGGGCAGCTTGTCG<br>AGCATTGGAATGAGAGAACGGTTG	
ROXY15	TTGGCGTGAACCCGACAATC	

Primer	Sequence 5'-3'	Reference
	GCCAAGCTGAGCCAATGCATAC	
ROXY16	QuantiTect QT00868077	Qiagen, Netherlands
SWEET11	GCCAATCTCAGTGGTTCGTCAAG<br>GAAGAGGACTGCTTGCCATGT	Rouina *et al*., 2021
TFL1	GCACAACAGATGCTACGTTTG<br>GTTGAAGTGATCTCTCGAAGG	Chen *et al.,* 2021
TGA1	ACGAACCTGTCCATCAATTCGG<br>CCATGGGAAGTATCCTCTGACACG	Li *et al.,* 2019
TGA4	AAAGTCGTTTGCGCAAGAAAGC<br>AGCATTGGTATCTACTCCGTTCCC	Li *et al.,* 2019
UBQ5	GACGCTTCATCTCGTCC<br>GTAAACGTAGGTGAGTCCA	Kesarwani *et al.,* 2007
UMAMIT35	Quantitect QT00883463	Qiagen, Netherlands

References

Alvarez, J.M., Riveras, E., Vidal, E.A., Gras, D.E., Contreras-Lopez, O., Tamayo, K.P., Aceituno, F., Gomez, I., Ruffel, S., Lejay, L., Jordana, X., and Gutierrez, R.A. (2014). Systems approach identifies TGA1 and TGA4 transcription factors as important regulatory components of the nitrate response of Arabidopsis thaliana roots. Plant J **80,** 1-13.

Sentmanat, M.F., Peters, S.T., Florian, C.P., Connelly, J.P., and Pruett-Miller, S.M. (2018). A Survey of Validation Strategies for CRISPR-Cas9 Editing. Sci Rep-Uk **8**.

Weiste, C., and Droge-Laser, W. (2014). The Arabidopsis transcription factor bZIP11 activates auxin-mediated transcription by recruiting the histone acetylation machinery. Nat Commun **5,** 3883.

Supplementary File S1: Promoter and primer sequences for ChIP analyses

ACTIN8 (reference)

ACTChIP+832fwd (Weiste and Droge-Laser, 2014)
GGTTTTCCCCAGTGTTGTTG

ACTChIP+994rev (Weiste and Droge-Laser, 2014)
CTCCATGTCATCCCAGTTGC

Weiste, C., and Droge-Laser, W. (2014). The Arabidopsis transcription factor bZIP11 activates auxin-mediated transcription by recruiting the histone acetylation machinery. Nat Commun **5,** 3883.

CLE3

TGA binding motifs:

TGACG or TGACGT, reverse orientation CGTCA or ACGTCA

-821
ccaatgaaaatcggttaattcgtcaaacgtcactacttgtctcaaagatttgttgtgctttactattttcgtcta
tttcatgcttccataatcagcttttacgtt**gacgtgctctttacaaatcgaagtg**gacgtggcggcaaccactt
atacttattatacatagaataaagctacaaaatatatagttaaggccctggtggattaatatatacat**cgtttaa**
ggttttagattggtctcctccaccttattttatgtgcattgccaactctgaaaacgtcatgccttatcttcattg
gttatatccatttggctc**catcaaccaaacgacgataatgattttccacatatc**ctatatacatttgggttcggt
agaattactacaatactcaaattgcttttaatttatgttttagtttatggaatatgaattgtgagtttttaaaa
aataatctcccaattaaatattattagcttttaagtttctatttcaagt**gaaatcagttagctatgcatgtcatt**
caatatagacataattttgttttcttccattcgttttacaattttaactaacagaaattatacattacgtcact
tactacgttcccactaaaaatagaggcaaatgcaat**caagtgatgacgacctttcaaaa**ataaatattaagagat
atataagaagggactagctaggtagatatgactagattccaacttaattaacgtatttaatttctgcatagatgg
acgttaattaggactttagcttctctataaataccgtagctattgagcttatatatggagatcacccccttaaaa
tcttcaaagactatatatctcacctttaagaactaagaggaagttacatatatATG

CLE3ChIP-821fwd
CCAATGAAAATCGGTTAATTCGTC

CLE3ChIP-691rev
CACTTCGATTTGTAAAGAGCACGTC

CLE3ChIP-603fwd
CGTTTAAGGTTTTAGATTGGTCTCC

CLE3ChIP-482rev
CATTATCGTCGTTTGGTTGATG

CLE3ChIP-322fwd
GAAATCAGTTAGCTATGCATGTCATTC

CLE3ChIP-163rev
TTTTGAAAGGTCGTCATCACTTG

NRT2.2

TGA binding motifs:

TGACG or TGACGTCA, reverse orientation CGTCA or ACGTCA or ACGTCAG, A-Box TACGTA

-1836
tacgtagtttgagaagatacaaatacataaattatatgaggtaacaaatagatgtgatgaaggaaaatcaaattc
ttttt**ccattagacggacactttaaatctg**aaattttcttaataaaacaactcatacgttgtcattacgtttttt
tctttgttgacgtcattacgtttttt**cttcttataccgtcacgttgttattacg**ttaaataaatcaaataacatt
ggattgaaatggtatatttgacctatagataacttagcataatgtataactaaaccaagcactttaccaagaaat
ttaataaaccattaattcgatcgtgtaataggtttataaatcattccaagattctaaatctgccccggcgtttcc
agactttaaatgttgtttttgactcgtatttgatttgacaattatgctgtttaaaataaaataaaatttgattt
gacaagtatctgatagacacatttactatatttatactacaatggtgtgacatcatcgataagttgactttccaa
aatcggacagcaaaatttgtcttttctcaaaccccaacaggcctcaacagagggaacaccg**gccacgtcaccaa**
gattgatgtatatacgctagtggcacggaaatggtacggcataaaatatacgaatacaactacttttttcttgtta
tatttaagacgtcatctacaactacattgtaatatattttgggttataatttagaacaaga**cgtatagctctacc**
tatcaattcgcaggagtcgatttaatacctacctatttctttatgtaactattcttataaacacgtgattaggaa
gtttcttgttcattgatcagttttatgtcacatatttatatttatgattttttttggtctgagttgtttaatcaaa
tttccaaagcagcaaccattttttccagcaactgaaaccatttaaaacaaaatttacccaaaaaattaagaagatt
ttgaatttcgaaatcagaccacctcaaaagtcaaatcttatatattttatgtttatatccaagcataatgcatat
attttcatagtttgtgggaaatccaaagtttctaaatttattcaaattaaagtcattaattaatccatgtttaaa
taatcaaaggacatgtgaaattgctaatgccagtcaaattacattacaaccaactaaaatatcgaacaaattaag
ttatgtctatatgtgctatacaaaattatctaatttttacagtgttatatatggaaaatatatgataagtttgtt
gaagtaataagcttattgatttttttagagacaactaatgtgcagctaaggccacaacaaattaaatcgtccagga
gacagcagtttattcaccacaatcattcctattcttgatacgatgtgatatagaattacattgatacccttaaga
aattatgaagaatatatggtctaaaagagaatcacaatgacatcgcttaaaaattatttaaatatggattttaat
gagttcacgatgtggtgcttgatcctgttataagataatatgtattacagacatgtaaaaaaaaaatccacttct
gaaacacaaaaggagaaaaggatacagttgtcacttgtcatctgagaggtgaatatcggtaacctttggggatta
gcgagagagtgggaaagatgcttgcggcgaatatggattcctcgaaaaaaatgtattttaatattgtttagtta
gccgttgagaatctccaaagaatactttttcttttttttttttgaaattgtcaaagctatacgaatcctatataat
ccctcgtgtgactaatatctagtacatcaatcatataaacttgaattttctcaaaggaacttgatacgtttaaaa
tacATGGGTTCTACTGATGAGCCCGGAAGTTCCATGCATGGAGTTACCGGTAGAGAACAGAGCTATGCTTTCTCG
GTAGATGGTAGTGAGCCGACCAACACAAAGAAAAAGTACAATCTGCCGGTGGACGCGGAGGATAAGGCAACGGTT
TTCAAGCTCTTCTCCTTCGCCAAACCTCACATGAGAACGTTCCACCTCTCGTGGATCTCTTTCTCCACATGTTTT
GTTTCGACGTTCGCAGCTGCACCACTTATCCCGATCATCAGGGAGAATCTTAACCTCACCAAACATGACATTGGA
AACGCTGGAGTTGCCTCCGTCTCGGGGAGTATCTTCTCTAGGCTCGTGATGGGAGCCGTGTGTGATCTTTTGGGT
CCTCGTTACGGTTGTGCCTTCCTTGTGATGTTGTCTGCCCCAACGGTGTTCTCCATGAGCTTCGTGAGTGACGCA
GCAGGCTTCATAACGGTGAGGTTCATGATTGGTTTTTGCCTGGCGACGTTTGTGTCTTGTCAATACTGGATGAGC
ACTATGTTCAACAGTCAGATCATCGGTCTGGTGAACGGGACAGCAGCCGGATGGGGAAACATGGGTGGCGGCATA
ACGCAGTTGCTCATGCCCATTGTGTATGAAATCATTAGGCGCTGCGGATCAACAGCGTTCACGGCCTGGAGGATC
GCCTTCTTTGTCCCCGGTTGGTTGCACATCATCATGGGAATCTTGGTGCTCACGCTAGGTCAAGATCTGCCAGGT
GGAAACAGAGCTGCCATGGAGAAAGCGGGAGAAGTTGCCAAAGACAAATTCGGAAAGgtatatctctatctacat
gaatatgtcaaaacaagttaggctaactatgtcatagatggatcggatataataggctaactcgtgatatcgtat
aaatcgtgtagATTCTATGGTACGCCGTTACAAATTACAGGACTTGGATTTTCGTTCTTCTGTATGGATATTCCA
TGGGAGTTGAGTTAAGCACAGACAATGTTATCGCCGAGTACTTCTTTGATAGgtttgttttctgtctcgattgga
aattgacaacttcacatatattcaggaataacataagagtaaaatttatattccctttatttttattttgtcaa
tcgttccctttatttaaaataaaaaaatggtatgttcagGTTTCACTTGAAGCTTCACACAGCGGGATTATA**G**
CAGCATGTTTCGGAATGGCCAATTTCTTTGCTCGTCCAGCAGGAGGCTGGGCATCTGACATTGCAGCCAAGCGCT
TCGGAATGCGAGGGAGGTTGTGGACT**TTGTGGATCATTCAGACGTCCG**GTGGTCTCTTTTGTGTGTGGCTCGGAC
GTGCCAACACCCTCGTCACTGCCGTTGTATCTATGGTCCTCTTCTCTTTAGGAGCACAAGCCGCTTGCGGAGCCA
CCTTTGCTATCGTGCCCTTTGTCTCCCGGCGAGCTCTAGGCATTATCTCGGGTTTAACCGGGGCTGGAGGGAACT
TTGGGTCAGGACTCACACAGCTCGTCTTTTTCTCGACTTCGCGCTTCACAACTGAAGAAGGGCTAACGTGGATGG
GAGTGATGATAGTTGCTTGCACGTTGCCTGTTACCTTAATCCACTTTCCTCAGTGGGGAAGCATGTTCTTCCCTC
CTTCCAACGATTCGGTCGACGCTACGGAGCACTATTATGTTGGCGAATATAGTAAGGAGGAGCAGCAGATTGGCA
TGCATTTAAAAAGCAAACTGTTTGCTGATGGAGCCAAGACCGAGGGAGGCAGCAGCGTCCACAAAGGAACGCAA
CCAACAATGCTTGAtcatgtgtcattgatatcaagaaattaataatttcacttatgtgaaatggacataaactgt
tggaaaataaagaaccatttctttcatcatttgcttt

NRT2.2ChIP-1756fwd
CCATTAGACGGACACTTTAAATCTG

NRT2.2ChIP-1633rev
CGTAATAACAACGTGACGGTATAAGAAG

NRT2.2ChIP-1249fwd
GCCACGTCACCAAGATTGATG

NRT2.2ChIP-1073rev
CCTGCGAATTGATAGGTAGAGCTATACG

NRT2.2CDS+1239fwd (Alvarez et al., 2014)
GCAGCATGTTTCGGAATGGC

NRT2.2CDS+1362rev (Alvarez et al., 2014)
CGGACGTCTGAATGATCCACAA

PER10

TGA binding motifs:

TGACGT, A-Box TACGTA

-1472
cttccaacatgggcacgtagtggaatatgttgcgcaatcctcttgatgtcataaacataatggacaaaaataatt
taaaatttacgtacacatgtttcttaaaactccaaattttatccggaaaaatgaatgttttgaaaa**gtctaagaa**
caagtgcgatattatgtgacaaacagaacaaatacaagaacaagagttagatcgtaagtataagttacattttcc
atcatttggttgtggtttggatgaaatgttctaccgagcataatggcagcgcttgtctaaaggtttcccccaaaa
tccaaatatcattttaaatcgagagggatcggagtatcatgatcacagttgaatactccttcttttatcaattag
acctcgagtaaattataaattccaccatccataagaaggtgataccatctttgggatcgatattatcgacctttta
gaatccgtttcaagtttggcatatatgatatatagttaaattccaaagtatatttgcaacggaccatctaatgaa
atcctcgtttatactatgaagaagttggaaacattacttaacatatgtatgtaga**cgagagcaataaatttcatc**
aaagcctacttttctattttggagacaaatttggctgataatctattaatttggcctttgaagtgtttctacttt
ctacgtataagcaaatttcttttaacctccctaaactttctggaagacttaaagtaactaatatc**ccattcctcc**
ttctttggatatttacaaaaatttctttagttcgtctaatttaggattctagacttgtatttgaacaaacccgta
gataatttattatttagga**tttgaatattgatccgaagccac**cccctataaatgatgagtcttgcatgagaacta
gttcacattgactttgatcagcttggtacgtacctaataacttaaaaagcttgctaattaccttataactta*cat*
*ccat*tgaagagataaag**ggagattgatggcggaatttag**accgatattttctccaatagatcattataaaccgga
ttacattttgctgtacataggctgcgctaaacaccccaaatgcgcacaacgttatcaataaaaatgaaaaagta
agggcatgagatgattagtttagatta**tttacatgcactaattctcccactattcaaaactta**tgagtatacatt
taacatttactaggttttggtttttatttgaatgtttgtatgacgtttacaaggaaaagttcgtatgacttggta
taatatatggcattttaatt**ctagataaaatcacatgctcacatgg**gtaataacaaatcatttattttgccaagt
gactgaactatctgacaaaaacctaatattacaaaaacctcaaaatttatcccactatattatcttatttatagt
ggcattcccactttcttaattatgcatgcatctccgaggcgcaccct tgttaaggcaaatatctatttttttaaaa
atacaccttgtttttggtgtataaatacaagctaaaatcatcatagcaatacactatcaatctacgatcatatat
ctttcatttcatcatcATG

PER10ChIP-1472fwd
CTTCCAACATGGGCACGTAGTG

PER10ChIP-1303rev
GTCACATAATATCGCACTTGTTCTTAGAC

PER10ChIP-892fwd
CGAGAGCAATAAATTTCATCAAAGC

PER10ChIP-706rev
TGTAAATATCCAAAGAAGGAGGAATGG

PER10ChIP-628fwd
TTTGAATATTGATCCGAAGCCAC

PER10ChIP-459rev
CTAAATTCCGCCATCAATCTCC

PER10ChIP-320fwd
TTTACATGCACTAATTCTCCCACTATTC

PER10ChIP-152rev
CCATGTGAGCATGTGATTTTATCTAG

PER71

TGA binding motifs:

reverse orientation ACGTCA, A-Box TACGTA

-349
atttgctatacatcaattaggcatgaacctaaattcaaaactcctatacgtaaacgatatttccatcattcgtta
cataatt**caatcaatttcacaccaagtaactatcaa**aacgaaaaaaaaaaaaaaaagtatcaaaacgaacaaatc
caaaagaaaatgcttttaaagtcaaccagtcgaccagctcacaacgcactgaccaacaccacgcttttgaatc
tcccacaaacggtgtcgtttttcatctatcccccacaaacaccaa**agccggttttgttgactcacac**gtcattct
ttgactaatcaatctcctctataaatagtactctataacgaactaactctactcactataa**cttaaacacacact**
tcatcttctctaaaaccctaaaatttaaacacaagATG

PER71ChIP-349fwd
ATTTGCTATACATCAATTAGGCATGAAC

PER71ChIP-239rev
TTGATAGTTACTTGGTGTGAAATTGATTG

PER71ChIP-79fwd
AGCCGGTTTTGTTGACTCACAC

PER71ChIP43rev
GGTTTTAGAGAAGATGAAGTGTGTGTTTAAG

SWEET11

TGA binding motifs:

TGACGTCA, reverse orientation CGTCA and ACGTCA, A-Box TACGTA

-1507
tgtcgtcatattttaatgataga**gaactagtggaattctcaattcattacatcatattta**tatggtgtatcgatt
tatattacttgtatatctatatataactaaacgcacaataacttaaaatataatttttcgctacacatgcaaatt
gattttgcaaggtatatagtcattca**gattgatcatttgtagttacttggttgg**ttttaaattagcaacgaaatc
acgtaagcgtcaacataaggaaacatgtagaataaaagtgaatgaactaatcactaattaagaaaaaatattagt
ttaaaccagtgttttcacgcccgacccgaaccgtccggtccgaccggttaacccgtaatccgaacatttttccgg
tttgggtttagtgctaaaacccaacaagttcaaaaccgaaaaaacccacaaaaactcgcaattaacccgtgaccc
ggtgaaccagttgaaccggtcgggtgtgggttttaaaaagttctcttgattttacaataaaatatgggattttag
actttattcaattatttttagaagtttttttcagttcttaaattcatttgatgttttagattttgttgatgacttt
acaataccgccttaaaattgaatcgaatggtgagagagacaaccgagattagttgttttaattcacgatgtttta
ttaatttatttctgtcattgatgatctcctaatatatttgttacggtgttagacatttattggatttcaaatttt
taaccactttattattattgtttgatttttctatatatatatatatatatatatatatatttgggtgtaaat
gattttatattttgttaaatataatataattttatatttgattaaatatgatatgattattttgttttatattt
ataaatactacaaattttttttattataaaatactctaaaatttctattagtattaaatttattaaataaatttt
aactaattaacccgtggtccaacccggttgacccgatgacccagtgacccagaagatagtccggttcatcgtccg
ggtcgggtttcaaaacattggtttaaaccgaagttggcaaatatacttattacgttgtgtaaataagttaaataa
taggattggtgaaaccctcaaaatcgtaaaatgattaggtcataattatgtcattgtatatatagagaatta**ga**
gatgcatacgtagaatctagacataatccttaagaaaatagcttgcctaaacagctgagattgacaagcattatt
aacataactaattatgttttatttcttaagccaaaacctaaaaaaggttatttggtaacagaattgttatttgta
cgtcaaaat**gtatatgatttggtaatggtttagcgtg**tgaattattgttgcgggtctttttttgttatacaacta
caatgacattaacgcttttaaaataaataagaaattcaaacattattcaaaatattattgttcacacaaacatt
tctcctttttccatctcccacacaaaccccattcctccctttagccctaaccccctatatatacgtgaagtccccg
aaacactaagctcgtgccattagcttttacattaagagaaaacacttgcaaaactaagtgctatctctcttcttc
ttccttctcctaacaacttatatacc ATG

SWEET11ChIP-1484fwd
GAACTAGTGGAATTCTCAATTCATTACATC

SWEET11ChIP-1304rev
CCAACCAAGTAACTACAAATGATCAATC

SWEET11ChIP-309fwd
GAGATGCATACGTAGAATCTAGACATAATCC

SWEET11ChIP-121rev
CACGCTAAACCATTACCAAATCATATAC

UMAMIT35

TGA binding motifs:

TGACGTCA, TGACGT, reverse orientation ACGTCA and ACGTCA, A-Box TACGTA

-1545
```
ctccacatttcttctgcctcaccatcgtgggaatatttcaaatgtcgtcaccattttaatcctaacattaatata
aaaggtgacgtttcattccccaactgctacgtcaaaggaaatgcgactcttaatcgattaagaaaaattcaagg
tcaaacattatatattcatttgtgtcttttaaaaaaacaatggttttgaaaacagttgttgagatttagggtttt
ccaattattttgttggtaccaacacagacataattgtcaatgaatagattgttcaatattgtcgtgatatataag
tggctaacaccttcttggaacgataaatcttttagctcattcatcttaaatcgaacaacattttcttgattgctt
atatccgtttgtatttactggactctagtactatatatgttaaaatattattggcttttaactaaaaatcagtta
ttcagtagattgacccagattttataaacgattgttggttctttcacacttttgatctgtgatcttaacacagc
ttttcaaagggatgaatgttgtcacgactctttcattttgaagttcagctcgtaaagacttatacacaaattatg
tttttttgatatataaaccacaacaataccgttttcggtgttaatttcaattgtcaatttgtcatcgtttatgaa
tccccttttctttaagcttggttttacaactgaaatttgacaacgaaaacaatattgttgtgagttatatattaa
aacagatatctgcgtatagagagactcgaactttggatctcttgcaatcaaataatattttgacttcttgcacta
aattgaagtacactatcaattttgcaatttaaaccacaacttatactaccaccaccattttgtggggatctg
ttttatttattataaaaatgatttttggagtttcaacaaatgggagttgatattgcatagaattagtaactcaaa
acgttttcagtttttcattatactaagcccaattcttccagaaacttggtattatatccgaacaagattaaacgt
aaatagttgcattaggatatttgtccactaattaatatttttatatacattaagaaaagcatcatattttggtta
tctatataaatgaaacataaatccaataaaaagtagtattaaacattttccatgtctattttataaatgatgt
tttaaaatattgtagaattaaatgaatattttttttgtagaaataattatgatacgtagaaaaaaaaaacgaaata
agtcttgtttaggattgtttccttgtttgtggaatgttgttgtgtaccaaaatttgatttattcttttcatattt
gcatatattttaaaatagaagggaatgttcttattttgtccaacagaaaactatttagatatattagtcataaga
gatgaggcgaaacaaattattagcttcatcacctctccaaaggctaactaatgactctctccacgtctagaaaaa
ccctcttcttcatttggccctccactcacatgcaaacacatataaatattaatgaagacacactaacattttaat
tgcaagacaagtagacaacctaacaaagttttgttgtggacttggtctctcatcttctccgagagagaaagagag
agagagacagagatcaacaaaatttcggctcatccaaggctgtgtattatcaacataagccgcacacaaacgaaA
TGATG
```

UMAMIT35ChIP-1545fwd
<u>CTCCACATTTCTTCTGCCTCACC</u>

UMAMIT35ChIP-1426rev
<u>GCATTTCCTTTGACGTAGCAGTTG</u>
