# Supplementary material for: Redox-inactive CC-type glutaredoxins interfere with TGA transcription factor–dependent repression of target promoters in roots
Source: Plant Cell. 2025 Mar 7;37(3):koaf038. doi: 10.1093/plcell/koaf038 (PMC11887855; doi:10.1093/plcell/koaf038)
Supplement: koaf038_Supplementary_Data [file tpc.11307.2024-s11.pdf › tpc.11307.2024-s11.pdf]

THE PLANT CELL

CEPD proteins activate N starvation response genes by interfering with the repressive activity of transcription factors TGA1 and TGA4

Corinna Thurow, Anja Pelizaeus, Pascal Mrozek, Ben Hossbach, Jelena Budimir, Kerstin Schmitt, Oliver Valerius, Gerhard Braus, and Christiane Gatz

Review Timeline:

Submission Date:	16-Sep-2024
Editorial Decision:	04-Nov-2024
Revision Received:	18-Dec-2024
Accepted:	16-Jan-2025

Prof. Dr. Christiane Gatz
Georg-August University Göttingen
Albrecht-von-Haller-Institut, Julia-Lermontowa-Weg 3
Göttingen 37077
Germany

Dear Dr. Gatz:

We have received reviews of your manuscript entitled "CEPD proteins activate N starvation response genes by interfering with the repressive activity of transcription factors TGA1 and TGA4." Thank you for submitting your best work to The Plant Cell. The editorial board agrees that the work you describe is substantive, falls within the scope of the journal, and may become acceptable for publication, pending revision and potential re-review.

We ask you to pay attention to the following points in preparing your revision: (1) All three reviewers emphasize the need for a thorough rewrite of the manuscript, particularly in the Results and Discussion sections. As noted by Reviewer 2, please temper conclusions that are not fully justified. The Discussion section, in its current form, mainly summarizes results. Instead, we felt that it should place the findings within a broader context, and this may result in a substantially shortening of this section, which would be beneficial in terms of readability. Reviewer 5 requests that all observations mentioned in the Results section be fully explained, but we would again encourage you to make the text shorter. (2) All three reviewers have also highlighted necessary revisions to the figures, particularly the final model. Based on the specific feedback from Reviewer 4 and Reviewer 5, please provide an updated model that connects the nitrogen (N) regimes with the interaction between CEPDs and TGAs. Ensure that sufficient details are provided in the legends as requested for all figures.

In addition, we find that the writing suffers from some problems with grammar and awkward phrasing, and in general lacks clarity and conciseness. We strongly encourage the use of a professional editing service to ensure that the work is presented in the best manner and reaches the broadest possible audience. Several editing services have agreed to extend a discount to authors coming to their website via this ASPB page https://aspb.org/aspb-journal-submission-editing-services/.

Please contact us if there are ambiguous comments or if you wish to discuss the revision.

Given the nature of the comments, we are offering you 60 days from when we have issued this decision to complete the revision. If a revision is not returned within this time frame, and if you have not been granted an extension, we will withdraw the manuscript, which will leave you free to submit the work elsewhere. If you need an extension, we encourage you to contact us at any point before the 60 days have passed.

When you are ready to submit the revised version, please upload a highlighted copy that indicates all changes made in response to the editor and reviewer recommendations. Include an itemized list of all changes made in response to each of the reviewer's suggestions in the "Response to Reviewers" section; please note that reviewers do not have access to your cover letter, nor was this decision letter shared with them.

Thank you for the privilege of reviewing your work. We look forward to receiving your revised manuscript.

On behalf of the editorial board,

Sonali Roy, Board of Reviewing Editors
Siobhán Brady, Senior Editor
Blake Meyers, Editor-in-Chief

The Plant Cell

Please note the following:

-The Plant Cell now requires authors to complete and submit an author revisions checklist upon submission of a revised manuscript. The aim of the checklist is to aid authors in preparing a high-quality manuscript, facilitate the review and assessment of revised manuscripts, and help to ensure that journal standards are maintained across the board. If your manuscript is accepted, the completed checklist will be published as supplemental material attached to the article online. Please download a copy of the checklist (pdf fillable form) at this link, for submission with your revised manuscript: https://tpc.msubmit.net/html/Author_Revisions_Checklist.pdf.

-Supplemental materials should be restricted to large datasets and tables, presentation of replicates, and validation of reagents, methods, or genotypes. Any data that are used to support the major claims must be in the main manuscript.

Supplemental figure legends must indicate what figure in the main manuscript is supported by the supplemental data presented. Please justify how each of the supplemental figures meet the criteria.

-Sampling methods and nature of "biological replicates" should be described precisely (i.e. different plants, parts of plants, pooled tissue, independent pools of tissue, sampled at different times, etc), along with a clear description of and rationale for any statistical analyses conducted. The reader should know exactly what was sampled; what forms the basis of the calculation of any means and statistical parameters reported. This is also necessary to ensure that proper statistical analysis was conducted.

-Want to add this revision deadline to your calendar? Click below!

--- Reviewer comments:

Reviewer #2 (Comments for the Author):

This resubmission by Thurow et al. adds a substantial amount of new data (at least 7 experiments), which address most of the comments of the three reviewers. As a result, the manuscript has changed quite significantly compared to the one submitted previously. On the one hand, the new data adds strength to the conclusions relating to the interaction between CEPDs/ROXYs and TGAs in controlling transcriptional responses under different N regimens. These findings are important especially in light of the fact that some experimental outcomes are not always easily reproduced in their entirety across different laboratories. This paper brings to light some of the reasons why precise reproducibility is not always achieved given the TGAs appear to be repressors in some conditions and activators in other conditions. Overall the experiments appear to be of high quality.
When it comes to clarity of presentation the paper as written in its current form is very complex and difficult to follow, which detracts significantly from its general accessibility. Whilst the experiments are generally clear the narrative is not. I suggest a thorough rewrite to increase the clarity of the message(s). There are also careless grammatical errors throughout.
The manuscript represents a significant body of work which is novel and important to understand the hierarchy of transcription networks that control plant responses to nutritional fluctuations by root to shoot and shoot to root communication but it needs to be written more clearly.
General points:
I find the Results section text unnecessarily long and complex throughout, and in sections are border-lining on 'Discussion' (e.g. lines 231-244). This tends to detract from the flow and digestibility of the paper. I recommend greatly simplifying the results text, and refining key messages to highlight core findings related to the interaction between CEPD/ROXYs and TGA in controlling low N transcriptional responses.
Specific points:
I find the writing style difficult to decipher and there are examples where the writing is contradictory. For example Lines 143 to 147. The root nitrate content was "nearly similar"...."with somewhat but significantly increased". How can something be nearly similar but significantly different ?? Should line 145 be "Unexpectedly, nitrate content was 7.6-fold higher in cepd roots when plant were grown on 1 mM No3" ???. Line 149 and elsewhere: do you mean "revealed" rather than "unravelled", which means worn, tattered, threadbare (not the intended meaning)? Lines 222-225 and 226-230 are also written in an unnecessarily complicated way. These are just some examples of the unclear writing style but there are many throughout the whole manuscript.
Figure 3
255-256 "Although ROXY 7, 8 and 9 do not act downstream of CEPR in Col-0 under our growth conditions, we still continue to call ROXYs 6-9 CEPDs". It is unfortunate that the ROXYs were renamed CEPD or CEPD-like, when they had been given prior names. Nevertheless, whilst it is fair to say CEPD transcript levels in cepr1-3 do not seem to correlate well with perturbations in NRT2.2/4 UMAMIT35 transcript levels, a stronger statement could be made if there was additional evidence like the analysis of a cepr1 cepd 'double' mutant. More broadly, I think that whilst the cepr1-3 data here is informative and interesting, it is a bit of a detour from the main narrative of the paper. One possible remedy is to move fig 3 to the supps and reduce the paragraph (ln 245-256) to a succinct summary of those results. On the other hand, I think this manuscript is hinting that the current model on how the CEPR1 receptor and its downstream outputs control responses is not as clean as we are led to believe. Perhaps this can be highlighted carefully (but briefly) in the discussion.
Fig 4. I think these results point to complexities and contradictions that have occurred in the literature and it offers a potential explanation for these complexities/contradictions. However, the message of this figure and the associated results text needs to be made more clear. It is a pity that some results are in Fig 2 and that the scales on Figure 4 are not uniform (especially for UMAMIT35 expression in (a) vs (b) (~10 fold difference).
Fig 6. Whilst it is fair to say based on the PCA that the large bulk of CEPD transcriptional activity depends on TGA1/4, I would caution against strong/absolute statements like "CEPDs cannot influence gene expression in the absence of TGA1/4" (Fig. 6 legend). If this were the case you would expect no genes to be differentially expressed between tga1/4 and cepd tga1/4. Was this the case in your experiments?

Fig 7 and lines 415-417 "In summary, our data suggest that regulation of promoter activity by CEPDs is due to alterations of the enhanceosome in the vicinity of TGA1/4 rather than to the control of TGA1/4 binding." - I feel this data is "consistent with" this notion, but was not tested directly (i.e. low N was used as a proxy for increased CEPD activity)
Fig 8 and 445-448. I found it confusing that CEPDs "do not interact with" TPL but "belong to the interactome of ROXY9". In general I found this paragraph was difficult to follow and requires simplification.
Fig. 9. The roxy10-15 data takes up a large section of results (ln 464-513) but is not mentioned in the Abstract.

Abstract: there are several results that are not mentioned in the Abstract, which focusses on CEPD proteins activating N starvation response genes by interfering with the repressive activity of transcription factors TGA1 and TGA4. There is no mention of TGA1 and TGA4 amplifying the expression of target promoters upon treatment of N-starved roots with nitrate or the role of CEPDs in interfering with this activation, the interactome studies, the competition of CEPDs with ROXY10-15 for control of a common regulatory network nor the overall physiological responses that are controlled by CEPDs/ROXYs in combination with TGA1 and 4. This detracts from the overall impact of the paper for a general audience.

Reviewer #4 (Comments for the Author):

Past studies have found that CEPD can participate in the nitrogen starvation response process by positively regulating genes such as NRT2.2, NRT2.4, but the components involved in the process remain unclear. This work found that TGA can activate or repress different target genes under different conditions. This is of reference significance for future researchers to explore the molecular regulatory network of plants in response to low nitrogen stress. However, there are also problems of insufficient experimental evidences and unclear logic.

Major comments
1, There is insufficient molecular experimental evidence for TGA1/4 positively or negatively regulates the downstream genes. Despite the authors submitting ChIP-qPCR experiments, more experimental evidences about the transcriptional activity of TAG1/4 are needed. The same problem arises for the transcriptional ability of the ROXY6/7/8/9. It is hoped that the results of the Dual Luciferase Reporter experiments for supplementation will be submitted.
2, The results of the phenotypic statistics covered in the article do not show the corresponding phenotypic maps (Fig1, FigS9), and although Fig. S2b shows a phenotype in soil conditions, the experiments for the phenotypic statistics of the article were all performed on vertically oriented MS plates.
3, Although the authors provide a working model, their presentation of the model is difficult to understand. The article investigates the different regulatory functions of CEPD and TGA under FN vs. LN, especially the repression/activation function of TGA on different downstream genes, which is an appealing part of the article, but unfortunately not reflected in the model.
4, The demonstration of the relationship between CEPDs and TGA1/4 is puzzling; cepd tga1/4 in Fig1 is consistent with tga1/4 implying that CEPDs are located upstream of and negatively regulate TGA, but the representation in the working model appears to be an activating effect.
5, It is not understood whether TGA1/4 functions as a transcription factor, whether TGA1/4 itself has transcriptional activation/repression activity, or whether it is transcriptionally regulated by binding to other proteins with transcriptional activation/repression activity (the working model demonstrates what appears to be other proteins exerting a transcriptional regulatory function).
6, The "antagonism" between CEPDs and ALWL ROXYS mentioned in the working model does not provide direct evidences that CEPDs inhibit ALWL ROXYS function at the transcriptional or protein level.
7, There is a lack of clarity in the authors' description of N imposition, using the 10 mM NO_3^- imposition model in the figure1, 4b, and 9 sections, while a mixed NO_3^-,NH_4^+,glutamine imposition model (FN/LN) is used in the other results sections.

Minor comments
1, The presentation of the graphs is confusing, for example line206-208 mentions fig2, but the correspondence between the text and the results in the graphs is not presented in a very clear way in the later sections. A specific identifier (e. g. Fig2a, b, c) should be added to make the article more detailed.
1, The Y-axis in Fig2 should use the form of two segments to clearly show the changes in expression under FN conditions.
2, Why does the expression of UMAMIT35 in Fig2 vs Fig4b show opposite changes in tag1/4 vs cepd tag1/4.
3, The expression of NRT2.2 and UMAMIT35 in cepd under NN conditions should be added in Fig4.
4, Fig5, " Two days later, shoots and roots were collected for RNA isolation", samples from roots and stems were used, but which part was used in experiment is unclear.
5, The labeling of significance in Fig7 is not intuitive enough to capture the difference in binding of TGA1 to fragments between FN and LN.

6, Fig. S2b, lacks scale and the image is not presented clearly.
7, Fig. S8a, lacks an internal reference protein for quantification.

Reviewer #5 (Comments for the Author):

Nitrogen starvation induces the release of mobile signals from root-to-shoot and vice versa. CEPDs have been identified as such a shoot-to-root signal leading to induction of genes coding for nitrate transporters involved in nitrate uptake. The exact molecular basis of this function, however, is unknown. By combining genetics with multiple individual and stacked mutants and analysis of the nitrogen content in the obtained plants, it is now shown that CEPDs act upstream of the transcription factors TGA1 and TGA4 to which they bind as a transcriptional activator. It is further shown that this function is redox-independent.
Analysis of the obtained data led to a working model that illustrates how CEPD and other ROXY-type glutaredoxins that contain an ALWL motif compete for the binding on TGA1/4 where they all compete against a hypothetical repressor. While CEPD binding leads to transcriptional activity, binding of ALWL-ROXYs causes repression due to the fact that these ROXYs bind additional co-repressors like Topless and thus recruit these to the promoter. While this illustrates the presumed interaction of CEPDs with TGA1/4, the model does not depict how these mechanisms might indeed be involved in the nitrogen starvation response. Would it be possible to depict at least that the abundance of CEPDs is linked to N starvation (which is currently mentioned in the figure legend)? Inj this context, it would be helpful for the reader to depict the situation for the wild-type for + and - N supply.
Some observations remain unexplained, especially the role of CEPDs and TGA1/4 in control of PER71 and other genes for which TGAs act as transcriptional activators that are repressed under participation of CEPDs. In the light of the large amount of data these remaining questions, however, do not damage the value of the manuscript, which in its current form does not leave any major gaps. There are a number of minor points though that need to be addressed:
l.37: 'Here show...': 'we' missing
l.40: The phrase is somewhat confusing. TGA are probably recruiting a 'repressor' and not a 'repression mechanism' as is stated here.
l.103-105: consider adding acronyms for the CEPD nomenclature to Fig. S1.
l.131-136: The description on how CRSPR mutagenesis was done is provided in the Methods part. It is not immediately intuitive though why a quadruple mutant is generated and the crossed with a double mutant? This necessarily implies quite a lot of breeding and selection. Maybe the authors can explain the rational of their approach a few words. Importantly, the molecular confirmation of the hexuple being clean for all six loci is missing.
l.143-144: 'nearly similar' and 'somewhat but significantly' are rather odd phrases that make the text vaguer than it should be. Consider replacing by 'approximately the same' and 'slightly' or something similar.
l.145-146: To avoid confusion, explicitly say what is compared to what. Here it should be stated that the refers to cepd roots compare to roots of all other lines grown on 1 mM nitrate.
l.146: 'The phenotype was...': Same point as before - always be explicit and specific to avoid confusion. Here it should be clearly stated which phenotype of which plant was reversed.
Fig. 1: consider using capital letters for labelling individual subpanels in a figure.
l.154: are the values for shoot:root ratio not provided in form of a figure (panel) with appropriate statistics? If so, why not?
l.183: Extended Data Set 1 is not available for evaluation.
l.229: 'partially sufficient'? Do you mean 'sufficient for partial activation'?
l.247: even if repetitive, it should be considered always mentioning the respective experimental condition under which gene expression was studied.
l.250-251: the compensating effect of ROXY8/9 induction for loss of ROXY6 is only an assumption at this point and should be marked as such. The current phrase 'roughly compensates', however, gives the impression that compensation is real, which cannot be deduced from the presented data.
l.280: delete 'are'
l.568: 'redox active'? Would 'redox responsive' be more appropriate to avoid the potentially misleading impression that an active redox switch is necessary?
l.601: 'on average' instead of 'in...'
l.630: 'a' should be 'are'
Fig. S1: The reference given here should correctly read 'El Baidouri et al., 2024'.
From the listed points it is obvious that some careful text editing is necessary before publication.

We thank all reviewers for their thorough reading the manuscript and for their insightful suggestions to enhance its quality. Below, we provide a detailed response to the concerns raised. We marked our responses in yellow to distinguish them from your comments.

Due to significant revisions made by us and the editing service, it is challenging to display all the track changes. However, in the manuscript with track changes, we have marked in red the areas, where the reviewers had pointed out deficiencies. In this version of the manuscript, please also find in yellow phrases containing background information and conclusions in the Results section and phrases that describe the results in the Discussion section. This should help illustrate that the text is not overly lengthy because of repetitive information in both sections. Line numbers in this response letter refer to the re-submitted manuscript.

-- Reviewer comments:

Reviewer #2 (Comments for the Author):

This resubmission by Thurow et al. adds a substantial amount of new data (at least 7 experiments), which address most of the comments of the three reviewers. As a result, the manuscript has changed quite significantly compared to the one submitted previously. On the one hand, the new data adds strength to the conclusions relating to the interaction between CEPDs/ROXYs and TGAs in controlling transcriptional responses under different N regimens. These findings are important especially in light of the fact that some experimental outcomes are not always easily reproduced in their entirety across different laboratories. This paper brings to light some of the reasons why precise reproducibility is not always achieved given the TGAs appear to be repressors in some conditions and activators in other conditions. Overall the experiments appear to be of high quality.

When it comes to clarity of presentation the paper as written in its current form is very complex and difficult to follow, which detracts significantly from its general accessibility. Whilst the experiments are generally clear the narrative is not. I suggest a thorough rewrite to increase the clarity of the message(s). There are also careless grammatical errors throughout.

The manuscript represents a significant body of work which is novel and important to understand the hierarchy of transcription networks that control plant responses to nutritional

fluctuations by root to shoot and shoot to root communication but it needs to be written more clearly.

General points:

I find the Results section text unnecessarily long and complex throughout, and in sections are border-lining on 'Discussion' (e.g. lines 231-244). This tends to detract from the flow and digestibility of the paper. I recommend greatly simplifying the results text, and refining key messages to highlight core findings related to the interaction between CEPD/ROXYs and TGA in controlling low N transcriptional responses.

After reading the manuscript again, we have to agree with the reviewer. We tried to streamline the text. Redundancy between Results and Discussion was mainly due to aspects of the Discussion being intermingled with the description of the results. We avoided this in several sections. Still, we felt that information serving to explain the rationale of the experiments had to be left. Moreover, we still mentioned important conclusions after description of the experiments.

Specific points:

I find the writing style difficult to decipher and there are examples where the writing is contradictory. For example Lines 143 to 147. The root nitrate content was "nearly similar"...."with somewhat but significantly increased". How can something be nearly similar but significantly different ?? Should line 145 be "Unexpectedly, nitrate content was 7.6-fold higher in cepd roots when plant were grown on 1 mM No3" ???. Line 149 and elsewhere: do you mean "revealed" rather than "unravelled", which means worn, tattered, threadbare (not the intended meaning)?

We rephrased the section describing Fig. 1 and replaced the term “unravelled” by other terms throughout the manuscript:

143ff To investigate whether CEPDs regulate TGA1 and/or TGA4 activity, we performed an epistasis analysis. Since defects in nitrate acquisition and growth of Arabidopsis (Nossen accession) seedlings are most pronounced when all four *CEPD* genes are mutated (Ota et al. 2020), we focused on the analysis of a *roxy6 roxy7 roxy8 roxy9* quadruple mutant, which we generated in the Col-0 background through clustered regularly interspaced short palindromic repeats (CRISPR)-associated nuclease 9 (Cas9)-based genome editing. We subsequently crossed this quadruple mutant, referred to hereafter as *cepd*, to the *tga1 tga4* double mutant (Shearer et al. 2012) to obtain the *cepd tga1 tga4* hextuple mutant.

The fresh weight of *cepd* shoots was lower than that of Col-0, regardless of nitrate supply, provided as 10 mM or 1 mM NO_3^- in Murashige and Skoog (MS) medium (Fig. 1A). It

was also lower when plants were grown on fertilized soil (Supplementary Fig. S2). Notably, the *cepd tga1 tga4* hextuple mutant produced the same amount of fresh biomass as Col-0, indicating that the poor growth of *cepd* is due to the repressive effect of TGA1 and/or TGA4 (from here on referred to as TGA1/4) on genes required for wild-type-like growth. The nitrate content was also lower in *cepd* shoots than in Col-0 regardless of the amount of nitrate in the medium. This phenotype also returned to Col-0 levels in *cepd tga1 tga4* (Fig. 1B). In the shoots of *tga1 tga4* seedlings grown on MS medium containing 10 mM NO_3^-, the nitrate content was slightly higher than that in Col-0 shoots, confirming the negative role of TGA1/4. In the roots of *cepd* seedlings grown in the presence of 10 mM NO_3^-, the nitrate content was similar to that of Col-0 while it was slightly higher in *tga1 tga4*. Unexpectedly, nitrate content was 7.6-fold higher in *cepd* roots than in wild-type roots in the presence of only 1 mM NO_3^-. Nitrate content also returned to Col-0 levels in *cepd tga1 tga4* (Fig. 1C).

The elevated nitrate level in the roots of *cepd* seedlings grown on 1 mM NO_3^- suggested that root-to-shoot allocation of N compounds was altered in this mutant. We confirmed this hypothesis when measuring total N per dry weight: the roots of *cepd* had a higher N content than Col-0, while the shoots of *cepd* had lower N levels than Col-0 shoots (Fig. 1D). However, this did not result in relatively better root growth of *cepd* (Fig. 1E). The N quantity per seedling was 2.5-fold lower in *cepd* than in Col-0, demonstrating that not only N allocation, but also nitrate uptake, was impaired (Fig. 1F).

Lines 222-225 and 226-230 are also written in an unnecessarily complicated way. These are just some examples of the unclear writing style but there are many throughout the whole manuscript.

We rephrased this section describing *NRT2.4* expression:

253ff The regulation of *NRT2.4* expression was similar to that of *NRT2.2*, showing the strongest differences among genotypes under LN conditions (Fig. 2C). *NRT2.4* expression was lower in *tga1 tga4* than in Col-0, indicating that TGA1/4 contributed to its transcriptional activation under LN conditions. TGA1/4 repressed *NRT2.4* expression in *cepd*, as revealed by the low *NRT2.4* expression levels in *cepd* and similarly high *NRT2.4* expression levels in *cepd tga1 tga4* and *tga1 tga4*. Constitutive expression of *ROYX9* under FN conditions was sufficient to activate the *NRT2.4* promoter, with a further induction under LN conditions (Fig. 2F).

Figure 3

255-256 "Although ROXY 7, 8 and 9 do not act downstream of CEPR in Col-0 under our growth conditions, we still continue to call ROXYs 6-9 CEPDs". It is unfortunate that the ROXYs were renamed CEPD or CEPD-like, when they had been given prior names. Nevertheless, whilst it is fair to say CEPD transcript levels in cepr1-3 do not seem to correlate well with perturbations in NRT2.2/4 UMAMIT35 transcript levels, a stronger statement could be made if there was additional evidence like the analysis of a cepr1 cepd 'double' mutant. More broadly, I think that whilst the cepr1-3 data here is informative and interesting, it is a bit of a detour from the main narrative of the paper. One possible remedy is to move fig 3 to the supps and reduce the paragraph (ln 245-256) to a succinct summary of those results. On the other hand, I think this manuscript is hinting that the current model on how the CEPR1 receptor and its downstream outputs control responses is not as clean as we are led to believe. Perhaps this can be highlighted carefully (but briefly) in the discussion.

We agree, that some confusion was raised by the nomenclature established by the Matsubayashi lab. Also, in their hands, CEPDL2 is not downstream of CEPR1. However, their findings were instrumental for our work, so we stayed with their nomenclature.

We have relocated the former Figure 3 to the Supplement, as suggested. This change also facilitates a more direct comparison of the data presented in Figures 2 and 4 (now Figure 3). We have condensed the corresponding description in the Results section and integrated the findings into the Discussion section.

Results: 272ff Since *ROXY6* (also named *CEPD1*) and *ROXY9* (also named *CEPD2*) act downstream of CEPR1 in the Nossen Arabidopsis accession (Ohkubo et al. 2017), we investigated the expression of *CEPD* genes and the target genes of their encoded peptides in *cepr1-3*, a mutant in the Col-0 background. Although the N starvation–induced expression of *NRT2.2*, *UMAMIT35*, and *NRT2.4* was compromised in *cepr1-3* (Supplementary Fig. S6A), relative *ROXY8* and *ROXY9* transcript levels were even slightly elevated in *cepr1-3* compared to Col-0. Only *ROXY6* expression levels were slightly reduced (Supplementary Fig. S6B). Although ROXY7, ROXY8, and ROXY9 do not appear to act downstream of CEPR1 in Col-0 under our growth conditions, we will continue to refer to ROXYs 6–9 as CEPDs.

Discussion 631f CEPDs act upstream of TGA1/4, but not necessarily downstream of CEPR1

Discussion 658 ff CEPDs (CEPD1 [also called ROXY6] and CEPD2 [also called ROXY9]) were originally described as acting downstream of CEPR1, since induction of *ROXY6* and *ROXY9* was severely compromised in the shoots of N-starved *cepr1-1* (in Nossen) (Ohkubo

et al. 2017). By contrast, N starvation–induced expression of *CEPD*s was either slightly elevated (*ROXY8*, *ROXY9*) or only slightly compromised (*ROXY6)* in *cepr1-3* (in Col-0) under our growth conditions. Since expression of CEPD target genes required the CEP–CEPR1 signaling cascade (Supplementary Fig. S6), we postulate that as yet unknown gene products downstream of CEPR1 are involved in N starvation–regulated gene expression. It remains unknown whether these are synthesized in shoots or roots and whether they positively or negatively affect gene expression.

Fig 4. I think these results point to complexities and contradictions that have occurred in the literature and it offers a potential explanation for these complexities/contradictions. However, the message of this figure and the associated results text needs to be made more clear. It is a pity that some results are in Fig 2 and that the scales on Figure 4 are not uniform (especially for UMAMIT35 expression in (a) vs (b) (~10 fold difference).

Since Fig. 3 has now been relocated to the Supplement, Fig. 2 and the former Fig. 4 might end up on the same page in the final version. We will try to make sure that this is realized.

The fold difference in the scale for *UMAMIT35* expression is due to the fact that expression of *UMAMIT35* is highly induced upon addition of nitrate.

Fig 6. Whilst it is fair to say based on the PCA that the large bulk of CEPD transcriptional activity depends on TGA1/4, I would caution against strong/absolute statements like "CEPDs cannot influence gene expression in the absence of TGA1/4" (Fig. 6 legend). If this were the case you would expect no genes to be differentially expressed between tga1/4 and cepd tga1/4. Was this the case in your experiments?

Only three genes are differentially expressed between *tga1 tga4* and *cepd tga1 tga4*. We mentioned this in the text

363ff In fact, only three genes were differentially expressed between *tga1 tga4* and *cepd tga1 tga4* ($\log_2$FC > 1 or < 1, *p* adj. < 0.05; Supplementary Data Set S2), strongly suggesting that CEPDs act mainly, if not exclusively, through TGA1/4.

Given that some unexpected mechanisms can merge on promoters in a hextuple mutant, I am convinced that CEPDs act exclusively through TGA1/4. However, since these absolute statements are frowned upon in the literature, we dampened the key massage in the legend and the heading of this paragraph.

356 The transcriptomes of *tga1 tga4*, *cepd* and *cepd tga1 tga4* indicate that CEPDs act mainly through TGA1/4

Legend former Fig. 6, now Fig. 5. CEPDs regulate target genes mainly through TGA1/4.

Fig 7 and lines 415-417 "In summary, our data suggest that regulation of promoter activity by CEPDs is due to alterations of the enhanceosome in the vicinity of TGA1/4 rather than to the control of TGA1/4 binding." - I feel this data is "consistent with" this notion, but was not tested directly (i.e. low N was used as a proxy for increased CEPD activity)

We rephrased to be more accurate.

451ff. In summary, our data suggest that differential expression of CEPD-TGA1/4-dependent genes under FN versus LN conditions is most likely not due to the control of TGA1/4 binding to their target promoters. Instead, we obtained evidence suggesting changes in the composition or topology of the enhanceosome in the vicinity of TGA1/4.

Fig 8 and 445-448. I found it confusing that CEPDs "do not interact with" TPL but "belong to the interactome of ROXY9". In general I found this paragraph was difficult to follow and requires simplification.

We rephrased and simplified this paragraph as suggested. We replaced the term “interactome” by “proxiome” throughout the manuscript in order to avoid the irritation. We moved parts of the paragraph from the Results to the Discussion.

478ff Volcano plots of the fold changes in protein abundance between the two sets of samples highlighted the most highly enriched proteins (Fig. 7). In addition to the expected enrichment of peptides derived from TGA1 (clade I), we also detected the enrichment of peptides from TGA2 (clade II), TGA3 (clade III) and TGA9 (clade IV) in the *TurboID-ROXY9* lines. Moreover, the Groucho/Tup1-type co-repressors TOPLESS (TPL) and TPL-RELATED proteins TPR2, TPR4, LEUNIG (LUG), and LEUNIG-HOMOLOG (LUH) (Liu and Karmarkar 2008) were part of the ROXY9 proxiome. SEUSS (SEU) and SEU-LIKE (SLK1), which interact with LEU and LUH (Stahle et al. 2009; Shrestha et al. 2014), were detected as well. In addition to transcriptional repressors, we identified conserved components of Switch defective/sucrose nonfermentable (SWI/SNF) chromatin remodeling complexes, namely ACTIN-RELATED PROTEIN 4 (ARP4) and ARP7 (Olave et al. 2002). Conspicuously, the DNA-binding proteins AT-HOOK MOTIF CONTAINING NUCLEAR LOCALIZED 20 (AHL20), AHL21, AHL23, and AHL27 were more than 16-fold more abundant in the *TurboID-ROXY9* lines compared to the control line. This analysis confirms the hypothesis that ROXY9 is recruited to chromatin through DNA-bound TGAs. There, it resides in the vicinity of transcriptional repressors and protein complexes required for chromatin opening or re-organization.

697ff Constitutive expression of TurboID-ROXY9 confirmed that ROXY9 is localized in close proximity to TGA-type transcription factors. Experimental evidence for an interaction between TGA1 and ROXY9 was previously provided in yeast two-hybrid assays and co-

immunoprecipitation experiments in protoplasts (Li et al. 2019). Thus, TGA1 most likely recruits ROXY9 to target promoters. Indeed, previous super-resolution microscopy experiments have detected the strong co-localization of ROXY1 with the active form of RNA polymerase II at euchromatin (Gutsche et al. 2017; Mass et al. 2020). In contrast to the TurboID control protein, TurboID-ROXY9 presumably resides in specific subnuclear compartments, where the relative concentration of transcriptional core components like chromatin remodeling complexes is higher than in the remaining nucleoplasm. In agreement with this idea, conserved members of the SWI/SNF chromatin remodeling complex (Huang et al. 2021; Fu et al. 2023) were significantly enriched among the peptides obtained from *TurboID-ROXY9* root extracts (Fig. 7, Supplementary Data Set S4). Proteins of the SWI/SNF complex have also been found in other proximity labeling experiments with the transcription factor BRASSINOSTEROID-INSENSITIVE 2 (BIN2) (Kim et al. 2023) and the transcriptional co-activator NONEXPRESSER OF PR GENES 1 (NPR1) (Powers et al. 2024). In contrast to these earlier proxiome datasets, four AT-hook DNA-binding proteins were enriched in *TurboID-ROXY9* root extracts. AHL family proteins bind to the minor groove of AT-rich regions, resulting in altered positioning of nucleosomes and bring distal DNA sequences into closer proximity by forming heterotrimers and homotrimers (Zhao et al. 2013). Hence, they might contribute to CEPD-dependent gene expression.

A striking observation was the high enrichment of peptides from transcriptional co-repressors of the Groucho/Tup1 category in the proximity labeling experiment (Fig. 7, Supplementary Data Set S4). This result was unexpected, since ROXY9 is a positive regulator of the expression of 350 genes that are activated upon N starvation. TPL and related proteins have also been identified in the proximity of transcriptional activators such a BIN2 (Kim et al. 2023), FAMA (Mair et al. 2019), and NPR1 (Powers et al. 2024). These findings can be explained by the assumption that transcriptional regulation is not necessarily due to the dissociation and association of co-repressors and co-activators from and to transcription factors at promoters, but rather to dynamic processes that alter the topology of the enhanceosome. This hypothesis is supported by the observation that *Drosophila melanogaster* Groucho interacts with components of chromatin remodeling complexes (Kwong et al. 2015). In our study, co-repressors that are recruited to the promoter in a TGA1/4-dependent manner would stay within the enhanceosome even after ROXY9-TurboID-mediated transcriptional activation. An alternative explanation is that ROXY9 resides in the vicinity of transcriptional co-repressors at promoters that are repressed by CEPDs (Fig. 4), albeit ROXY9 does not contain the TPL-interacting ALWL motif (Uhrig et al. 2017).

Fig. 9. The roxy10-15 data takes up a large section of results (ln 464-513) but is not mentioned in the Abstract.

Abstract: there are several results that are not mentioned in the Abstract, which focusses on CEPD proteins activating N starvation response genes by interfering with the repressive activity of transcription factors TGA1 and TGA4. There is no mention of TGA1 and TGA4 amplifying the expression of target promoters upon treatment of N-starved roots with nitrate or the role of CEPDs in interfering with this activation, the interactome studies, the competition of CEPDs with ROXY10-15 for control of a common regulatory network nor the overall physiological responses that are controlled by CEPDs/ROXYs in combination with TGA1 and 4. This detracts from the overall impact of the paper for a general audience.

We rephrased the Abstract. Still, it is impossible to mention all the Results. We included ROXYs 10-15.

40 ff In wild-type plants, this repressive complex is nonfunctional and gene expression may be regulated by the N supply-regulated ratio of CEPDs over opposing ROXYs containing the TOPLESS-interacting ALWL motif.

Reviewer #4 (Comments for the Author):

Past studies have found that CEPD can participate in the nitrogen starvation response process by positively regulating genes such as NRT2.2, NRT2.4, but the components involved in the process remain unclear. This work found that TGA can activate or repress different target genes under different conditions. This is of reference significance for future researchers to explore the molecular regulatory network of plants in response to low nitrogen stress. However, there are also problems of insufficient experimental evidences and unclear logic.

Major comments

1, There is insufficient molecular experimental evidence for TGA1/4 positively or negatively regulates the downstream genes. Despite the authors submitting ChIP-qPCR experiments, more experimental evidences about the transcriptional activity of TAG1/4 are needed. The same problem arises for the transcriptional ability of the ROXY6/7/8/9. It is hoped that the results of the Dual Luciferase Reporter experiments for supplementation will be submitted.

We have provided genetic evidence for the positive and negative regulatory roles of TGA1/4 and ROXY6/7/8/9 on target gene expression. While it is established that the N terminus of TGA1 possesses transcriptional activation capacity, this activity appears to be masked, depending on the specific enhanceosome that TGA1/4 is part of. Given that the composition of an enhanceosome can differ between transiently transformed protoplasts and differentiated root cells, we have chosen not to address the reviewer's questions in a transient assay system.

2, The results of the phenotypic statistics covered in the article do not show the corresponding phenotypic maps (Fig1, FigS9), and although Fig. S2b shows a phenotype in soil conditions, the experiments for the phenotypic statistics of the article were all performed on vertically oriented MS plates.

The primary focus of our analysis was on plants grown on MS medium, as this system allows for precise control of N supply. The inclusion of data from soil-grown plants serves to demonstrate that the cepd and cepd tga1 tga4 phenotypes are not limited to the specific experimental conditions of plants grown on synthetic substrate, but are also observed in plants grown in soil."

3, Although the authors provide a working model, their presentation of the model is difficult to understand. The article investigates the different regulatory functions of CEPD and TGA under FN vs. LN, especially the repression/activation function of TGA on different downstream genes, which is an appealing part of the article, but unfortunately not reflected in the model.

Our main scientific question was whether CEPDs act by modulating TGA1/4 activity and whether this involves redox modifications. The different roles of TGA1/4 on different promoters turned out to complicate the story and it is beyond the scope of this manuscript to decipher interacting proteins on the different promoters under different conditions. Although transcriptome analysis displays patterns of gene expression, the combinatorial action of transcription factors allows individual transcription factors playing different roles even at one promoter, as shown for UMAMIT35 (Figs 2 and 3). It is almost impossible to display all the different possible ways how TGA1/4 influence gene in a unifying model, especially since we do not have enough data on other interacting proteins. Therefore, we focussed on the likely mode of action of CEPDs and on the function of TGA1/4 as anchor proteins for CEPDs.

As requested, we updated the model to explain the role of CEPDs in plants under FN versus LN conditions.

4, The demonstration of the relationship between CEPDs and TGA1/4 is puzzling; cepd tga1/4 in Fig1 is consistent with tga1/4 implying that CEPDs are located upstream of and negatively regulate TGA, but the representation in the working model appears to be an activating effect.

As illustrated in the model, CEPDs activate gene expression by negatively acting on the negative activity of TGA1/4, i.e. most likely by displacing the strong repressive complex. As soon as this repressive complex is displaced, other not yet identified mechanisms determine the expression pattern observed in *tga1 tga4*.

5, It is not understood whether TGA1/4 functions as a transcription factor, whether TGA1/4 itself has transcriptional activation/repression activity, or whether it is transcriptionally regulated by binding to other proteins with transcriptional activation/repression activity (the working model demonstrates what appears to be other proteins exerting a transcriptional regulatory function).

This remark relates to point 3. We do not have the technology to define the components and the topology of the enhanceosome at a specific promoter. The TurboID approach is, unfortunately, not promoter specific. As pointed out by the reviewer, we do not know whether TGA1/4 act as activators by recruiting appropriate mediator subcomplexes or by repressing a repressor. Our analysis focussed on CEPDs, especially in the context of their potential redox activity.

6, The "antagonism" between CEPDs and ALWL ROXYS mentioned in the working model does not provide direct evidences that CEPDs inhibit ALWL ROXYS function at the transcriptional or protein level.

We provide in fact only indirect experimental evidence that the regulation of gene expression on FN vs LN is due to the antagonistic action of CEPDs and ALWL ROXYs. Given the high similarity between these proteins, it is very likely that they can compete for binding to TGAs.

7, There is a lack of clarity in the authors' description of N imposition, using the 10 mM NO3- imposition model in the figure1, 4b, and 9 sections, while a mixed NO3-,NH4+,glutamine imposition model (FN/LN) is used in the other results sections.

The Genevestigator results (Supplementary Fig. S3) on the expression pattern of ROXYs implied that loss of function phenotypes might be most apparent under the growth conditions described in the data base. Thus, we grew plants on the medium with ammonium and

glutamine in most of our experiments. When measuring nitrate content, we switched to medium with nitrate only (Fig. 1). The experiments for Fig. 3 were done in the absence of these compounds because we wanted to come closer to the conditions of Alvarez et al, in order to study the role of TGAs and CEPDs upon nitrate repletion. For Fig. 8, we removed the glutamine and ammonium, since they mask the repressive effect of TGA1/4 at some promoters and thus might have masked the effect of ROXYs 10-15.

Minor comments

1, The presentation of the graphs is confusing, for example line206-208 mentions fig2, but the correspondence between the text and the results in the graphs is not presented in a very clear way in the later sections. A specific identifier (e. g. Fig2a, b, c) should be added to make the article more detailed.

We added the specific identifiers to all of the figures.

1, The Y-axis in Fig2 should use the form of two segments to clearly show the changes in expression under FN conditions.

We prefer to leave it as it is to document the big differences in transcript levels between FN and LN. We added the expression pattern of *NRT2.2* in plants transferred from FN to FN in an altered Supplementary Fig. S5.

2, Why does the expression of UMAMIT35 in Fig2 vs Fig4b show opposite changes in tag1/4 vs cepd tag1/4.

We do not understand the question. Both mutants are not significantly different from each other in both Figures.

3, The expression of NRT2.2 and UMAMIT35 in cepd under NN conditions should be added in Fig4.

NN + KCl is the proper control for NN + KNO3. Inclusion of the NN control would demonstrate a potential influence of additional KCl, which would add even more information to this paper. Still, expression levels in Col-0 under NN (Former Fig. 4a) and NN + KCl (Former Fig 4b), now Fig. 3A/B and C/D, are similar and a reduced expression in *tga1 tga4* is also documented.

4, Fig5, "Two days later, shoots and roots were collected for RNA isolation", samples from roots and stems were used, but which part was used in experiment is unclear.

We are sorry for the mistake, we corrected this, only results from root RNA are shown.

5, The labeling of significance in Fig7 is not intuitive enough to capture the difference in binding of TGA1 to fragments between FN and LN.

The significance does not refer to the difference between FN and LN, but to the difference compared to the *ACTIN8* control. Since there is no difference between FN and LN, we summed up the all four values (2 samples from plants grown on FN and 2 samples from plants grown on LN) for statistical analysis. This was written in the legend:

"Data shown in (A) are from two biological replicates…….Asterisks denote significant differences compared to *ACTIN8* (unpaired *t* test *p*-value < 0.01 (A)……..). The mean of all four values (FN and LN) was used for the statistics…."

6, Fig. S2b, lacks scale and the image is not presented clearly.

We included the scale bar.

7, Fig. S8a, lacks an internal reference protein for quantification.

We added a photo of the Coommassie-stained PVDF membrane.

Reviewer #5 (Comments for the Author):

Nitrogen starvation induces the release of mobile signals from root-to-shoot and vice versa. CEPDs have been identified as such a shoot-to-root signal leading to induction of genes coding for nitrate transporters involved in nitrate uptake. The exact molecular basis of this function, however, is unknown. By combining genetics with multiple individual and stacked mutants and analysis of the nitrogen content in the obtained plants, it is now shown that CEPDs act upstream of the transcription factors TGA1 and TGA4 to which they bind as a transcriptional activator. It is further shown that this function is redox-independent. Analysis of the obtained data led to a working model that illustrates how CEPD and other ROXY-type glutaredoxins that contain an ALWL motif compete for the binding on TGA1/4 where they all compete against a hypothetical repressor. While CEPD binding leads to transcriptional activity, binding of ALWL-ROXYs causes repression due to the fact that these ROXYs bind additional co-repressors like Topless and thus recruit these to the promoter. While this illustrates the presumed interaction of CEPDs with TGA1/4, the model does not depict how these mechanisms might indeed be involved in the nitrogen starvation response.

Would it be possible to depict at least that the abundance of CEPDs is linked to N starvation (which is currently mentioned in the figure legend)? Inj this context, it would be helpful for the reader to depict the situation for the wild-type for + and - N supply.

We updated the model to explain the role of CEPDs in plants under high N versus low N conditions. Now Fig. 10, illustrating that higher CEPD levels are present when plants are grown on low N conditions.

Some observations remain unexplained, especially the role of CEPDs and TGA1/4 in control of PER71 and other genes for which TGAs act as transcriptional activators that are repressed under participation of CEPDs. In the light of the large amount of data these remaining questions, however, do not damage the value of the manuscript, which in its current form does not leave any major gaps. There are a number of minor points though that need to be addressed:

l.37: 'Here show...': 'we' missing

corrected.

l.40: The phrase is somewhat confusing. TGA are probably recruiting a 'repressor' and not a 'repression mechanism' as is stated here.

Repressive mechanism was replaced by repressive complex

l.103-105: consider adding acronyms for the CEPD nomenclature to Fig. S1.

41 We added the acronyms for CEPDs.

l.131-136: The description on how CRSPR mutagenesis was done is provided in the Methods part. It is not immediately intuitive though why a quadruple mutant is generated and the crossed with a double mutant? This necessarily implies quite a lot of breeding and selection. Maybe the authors can explain the rational of their approach a few words. Importantly, the molecular confirmation of the hexuple being clean for all six loci is missing.

We generated the *cepd* (quadruple mutant) and crossed it with *tga1 tga4*. We could have also generated the *cepd* alleles in *tga1 tga4*, and subsequently selected for *cepd* in the wt background, but we generated the *cepd* mutant before we asked the question whether CEPDs act upstream of TGA1/4. We added information in the methods section (Line 842) that all six loci were missing in the hextuple mutant (Supplementary Fig. S23).

l.143-144: 'nearly similar' and 'somewhat but significantly' are rather odd phrases that make the text vaguer than it should be. Consider replacing by 'approximately the same' and 'slightly' or something similar.

l.145-146: To avoid confusion, explicitly say what is compared to what. Here it should be stated that the refers to cepd roots compare to roots of all other lines grown on 1 mM nitrate.
l.146: 'The phenotype was...': Same point as before - always be explicit and specific to avoid confusion. Here it should be clearly stated which phenotype of which plant was reversed.
Fig. 1: consider using capital letters for labelling individual subpanels in a figure.
l.154: are the values for shoot:root ratio not provided in form of a figure (panel) with appropriate statistics? If so, why not?

We rephrased the whole paragraph and added the shoot:root ratio with error bars as an insert in Fig. 1E. We also labelled the subpanels of each figure throughout the manuscript.

152ff To investigate whether CEPDs regulate TGA1 and/or TGA4 activity, we performed an epistasis analysis. Since defects in nitrate acquisition and growth of Arabidopsis (Nossen accession) seedlings are most pronounced when all four *CEPD* genes are mutated (Ota et al. 2020), we focused on the analysis of a *roxy6 roxy7 roxy8 roxy9* quadruple mutant, which we generated in the Col-0 background through clustered regularly interspaced short palindromic repeats (CRISPR)-associated nuclease 9 (Cas9)-based genome editing. We subsequently crossed this quadruple mutant, referred to hereafter as *cepd*, to the *tga1 tga4* double mutant (Shearer et al. 2012) to obtain the *cepd tga1 tga4* hextuple mutant.

The fresh weight of *cepd* shoots was lower than that of Col-0, regardless of nitrate supply, provided as 10 mM or 1 mM NO_3^- in Murashige and Skoog (MS) medium (Fig. 1A). It was also lower when plants were grown on fertilized soil (Supplementary Fig. S2). Notably, the *cepd tga1 tga4* hextuple mutant produced the same amount of fresh biomass as Col-0, indicating that the poor growth of *cepd* is due to the repressive effect of TGA1 and/or TGA4 (from here on referred to as TGA1/4) on genes required for wild-type-like growth. The nitrate content was also lower in *cepd* shoots than in Col-0 regardless of the amount of nitrate in the medium. This phenotype also returned to Col-0 levels in *cepd tga1 tga4* (Fig. 1B). In the shoots of *tga1 tga4* seedlings grown on MS medium containing 10 mM NO_3^-, the nitrate content was slightly higher than that in Col-0 shoots, confirming the negative role of TGA1/4. In the roots of *cepd* seedlings grown in the presence of 10 mM NO_3^-, the nitrate content was similar to that of Col-0 while it was slightly higher in *tga1 tga4*. Unexpectedly, nitrate content was 7.6-fold higher in *cepd* roots than in wild-type roots in the presence of only 1 mM NO_3^-. Nitrate content also returned to Col-0 levels in *cepd tga1 tga4* (Fig. 1C).

The elevated nitrate level in the roots of *cepd* seedlings grown on 1 mM NO_3^- suggested that root-to-shoot allocation of N compounds was altered in this mutant. We

confirmed this hypothesis when measuring total N per dry weight: the roots of *cepd* had a higher N content than Col-0, while the shoots of *cepd* had lower N levels than Col-0 shoots (Fig. 1D). However, this did not result in relatively better root growth of *cepd* (Fig. 1E). The N quantity per seedling was 2.5-fold lower in *cepd* than in Col-0, demonstrating that not only N allocation, but also nitrate uptake, was impaired (Fig. 1F).

l.183: Extended Data Set 1 is not available for evaluation.

l.229: 'partially sufficient'? Do you mean 'sufficient for partial activation'?

We rephrased:

258f: Constitutive expression of *ROYX9* under FN conditions was sufficient to activate the *NRT2.4* promoter, with a further induction under LN conditions (Fig. 2F).

l.247: even if repetitive, it should be considered always mentioning the respective experimental condition under which gene expression was studied.

We and also the editing service (Plant editors) payed attention to this point.

l.250-251: the compensating effect of ROXY8/9 induction for loss of ROXY6 is only an assumption at this point and should be marked as such. The current phrase 'roughly compensates', however, gives the impression that compensation is real, which cannot be deduced from the presented data.

We rephrased:

275ff: Although the N starvation–induced expression of *NRT2.2*, *UMAMIT35*, and *NRT2.4* was compromised in *cepr1-3* (Supplementary Fig. S6A), relative *ROXY8* and *ROXY9* transcript levels were even slightly elevated in *cepr1-3* compared to Col-0. Only *ROXY6* expression levels were slightly reduced (Supplementary Fig. S6B).

l.280: delete 'are'

This paragraph was rephrased

l.568: 'redox active'? Would 'redox responsive' be more appropriate to avoid the potentially misleading impression that an active redox switch is necessary?

The term redox active seems to be an often used technical term that describes that cysteines are prone to being readily oxidized or reduced, depending on the redox potential of their environment. Redox responsive is correct, but not often used.

l.601: 'on average' instead of 'in...'

corrected

l.630: 'a' should be 'are'

corrected

Fig. S1: The reference given here should correctly read 'El Baidouri et al., 2024'.

corrected

From the listed points it is obvious that some careful text editing is necessary before publication.

We streamlined the text and submitted the latest version to the editing service Plant Editors.

Dear Dr. Christiane Gatz:

We are pleased to inform you that your paper entitled "CEPD proteins activate N starvation response genes by interfering with the repressive activity of transcription factors TGA1 and TGA4" has been accepted for publication in The Plant Cell, pending a final editorial review by a science editor. At this stage, your manuscript will be evaluated by a science editor with respect to its presentation of scientific content, compliance with journal policies, and presentation for a broad readership. The Plant Cell has contracted with Plant Editors (planteditors.com) to provide this service to our authors, and you will soon receive additional information on this process.

Please note that each author needs to link their ORCID identifier to their account in the system before your manuscript can be published. If any authors do not have an ORCID linked to their account, they will receive a message with a link to complete this task. Please ensure that ALL of your coauthors have completed this task as soon as possible.

LICENSE INFORMATION Shortly after your paper is sent to production, you will receive an email from our publisher's author support team, **SciPris**, with information on article processing charges, as well as the types of Open Access license you would be able to purchase. The accepted version of your manuscript will not appear on our Advance Articles page until the license is selected and signed, so please look out for an email from SciPris. For more information see the Article Publication Fees section of our author guidelines: https://academic.oup.com/plcell/pages/General-Instructions

The Plant Cell and The Arabidopsis Information Resource (TAIR) are collaborating to collect functional annotation data about Arabidopsis genes from authors. This includes information about the gene's molecular function (e.g., kinase activity, ATP synthetase activity), the biological process/es it is involved in (e.g., endosperm development, threonine biosynthesis), its subcellular location (e.g., nucleus, ER), anatomical or developmental expression pattern (e.g., leaf, ovule, flower stage 10, seedling stage), or its partner in a protein-protein interaction (e.g., AT1G01010 interacts with AT1G01020).

If your paper contains results falling into one or more of these categories for Arabidopsis genes, we request that you now submit these data for inclusion in TAIR by filling in the form provided at the following URL: https://www.arabidopsis.org/doc/submit/functional_annotation/123. If you need further clarification on what types of data can be submitted please contact curator@arabidopsis.org.

Finally, we encourage your submission of artwork for the journal cover. If there is an image or illustration related to your paper that you would like to have considered, please email it in .pdf or .tif format to clowe@aspb.org. An image title, brief, 2-3 sentence description of the image, and the name of the person credited for the image are also encouraged. If your image is selected to appear on the online cover of the journal, you will be contacted with the scheduled issue date and further information.

We look forward to seeing your paper published.

On behalf of the editorial board,

Sonali Roy, Board of Reviewing Editors
Siobhan Brady, Senior Editor
Pablo Manavella, Editor in Chief
The Plant Cell

=====
IMPORTANT REMINDER: PEER REVIEW REPORTS
=====

If you opted to publish a peer review report along with your article during the original submission process, it will be prepared by the editorial staff and publicly posted with your manuscript, inside the zip file that contains any other supplemental material. As a reminder, the peer review report is a public record of all comments from editors and reviewers, as well as your prior responses, as you received them in the decision letters for each draft of your manuscript. If you agreed to publish this report and have changed your mind, or are not sure if you selected this option, please contact the editorial office as soon as possible before signing the license agreement from our publisher.

=====
REVIEWER COMMENTS
=====

Minor Comments

Make sure font size is a minimum of 6-8 in all figures. Some text is not legible in Figure 10 and Figure 7.

Line 455- 456 Sentence incomplete. "Instead, we obtained evidence suggesting changes in the composition or topology of the enhanceosome in the vicinity of TGA1/4... are responsible for..."

----FOR ASPB OFFICE USE ONLY (DO NOT EDIT)----
MSID: 41267
Corresponding Author: Prof. Dr. Gatz
